# Precision Thermostability Predictions: Leveraging Machine Learning for Examining Laccases and Their Associated Genes

**DOI:** 10.3390/ijms252313035

**Published:** 2024-12-04

**Authors:** Ashutosh Tiwari, Dyah Ika Krisnawati, Widodo, Tsai-Mu Cheng, Tsung-Rong Kuo

**Affiliations:** 1 International Ph.D. Program in Biomedical Engineering, College of Biomedical Engineering, Taipei Medical University, Taipei 11031, Taiwan; d845112008@tmu.edu.tw; 2 Department of Nursing, Faculty of Nursing and Midwifery, Universitas Nahdlatul Ulama Surabaya, Surabaya 60237, East Java, Indonesia; dyahkrisna77@gmail.com; 3 Sekolah Tinggi Teknologi Pomosda, Nganjuk 64483, East Java, Indonesia; widodoido7@gmail.com; 4 Graduate Institute for Translational Medicine, College of Medical Science and Technology, Taipei Medical University, Taipei 11031, Taiwan; 5 Taipei Heart Institute, Taipei Medical University, Taipei 11031, Taiwan; 6 Cardiovascular Research Center, Taipei Medical University Hospital, Taipei Medical University, Taipei 11031, Taiwan; 7 Graduate Institute of Nanomedicine and Medical Engineering, College of Biomedical Engineering, Taipei Medical University, Taipei 11031, Taiwan

**Keywords:** laccase thermostability, computational genomics, machine learning, random forest regression, convolutional neural network, enzyme engineering

## Abstract

Laccases, multi-copper oxidases, play pivotal roles in the oxidation of a variety of substrates, impacting numerous biological functions and industrial processes. However, their industrial adoption has been limited by challenges in thermostability. This study employed advanced computational models, including random forest (RF) regressors and convolutional neural networks (CNNs), to predict and enhance the thermostability of laccases. Initially, the RF model estimated melting temperatures with a training mean squared error (MSE) of 13.98, and while it demonstrated high training accuracy (93.01%), the test and validation MSEs of 48.81 and 58.42, respectively, indicated areas for model optimization. The CNN model further refined these predictions, achieving lower training and validation MSEs, thus demonstrating enhanced capability in discerning complex patterns within genomic sequences indicative of thermostability. The integration of these models not only improved prediction accuracy but also provided insights into the critical determinants of enzyme stability, thereby supporting their broader industrial application. Our findings underscore the potential of machine learning in advancing enzyme engineering, with implications for enhancing industrial enzyme stability.

## 1. Introduction

**Figure 1:**
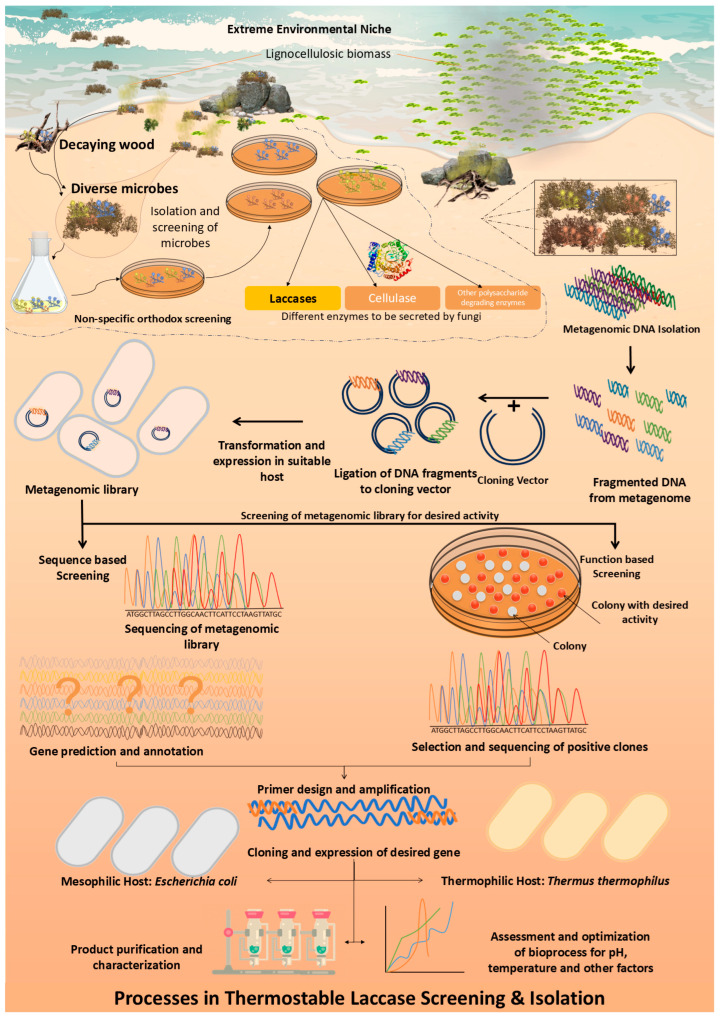
The flowchart of general steps and experimental advances of culture-dependent methods in thermostable enzyme discovery.

In the realm of biotechnology and industrial applications, the quest for robust enzymes capable of operating under harsh conditions has spotlighted laccases—versatile, multi-copper oxidase enzymes that catalyze the oxidation of a myriad of substrates from phenols to anilines [1]. These enzymes, belonging to the blue copper protein family, exhibit remarkable substrate specificity across various pH levels, thanks to their unique copper centers. Produced by a diverse array of microorganisms—including fungi, bacteria, and algae—fungal laccases, in particular, have garnered significant attention due to their high redox potential [2]. However, the practical application of these enzymes is often hampered by their sensitivity to environmental extremes—be it temperature, pH, or the presence of inhibitory substances, limiting their industrial utility [3]. By addressing these challenges, recent strides in protein engineering, directed evolution, and enzyme immobilization have attempted to bolster the thermostability and efficiency of laccases, broadening their application horizon. Moreover, exploring metagenomics, especially from uncultivated organisms in extreme environments, has emerged as fertile ground for unearthing novel laccase genes, pushing the boundaries of biocatalysis [4]. We illustrate these procedures in Figure 1. Thermostability, or an enzyme’s resilience against denaturation at elevated temperatures, is a cornerstone for enhancing industrial enzyme efficiencies. High-temperature processes not only increase reaction rates but also reduce the need for cooling, cutting down costs and spurring productivity [5]. Nature’s repertoire of thermophilic organisms, exemplified by the *Thermus* genus, thrives in hot environments, offering a blueprint for the development of thermostable enzymes [6]. These organisms exhibit metabolic, and protein structures adapted to high temperatures, presenting an invaluable resource for biotechnological exploitation. However, the quest to decode the determinants of protein thermostability has unveiled a complex landscape, where factors such as amino acid compositions, ionic interactions, and structural attributes intertwine, challenging the generalization of thermal stability mechanisms [7]. This complexity underscores the necessity for species-specific investigations and highlights the potential of in silico methods. These computational approaches, leveraging codon and amino acid analytics, provide a promising avenue for comprehensive thermostability analysis, circumventing the limitations inherent in traditional experimental methodologies.

Moreover, this introductory discourse set the stage for our investigation, which merged the potency of machine learning with deep learning techniques to delve into the predictive modeling of laccase thermostability. Through an intricate analysis of genomic data and the application of advanced computational models, we endeavored to enhance our understanding of laccase properties, paving the way for the development of enzymes with superior thermostability for industrial applications. Our study represents a convergence of biotechnology and computational innovation, aiming to contribute to the sustainable advancement of industrial biocatalysis.

## 2. Results

### 2.1. Model Performance

**Figure 2:**
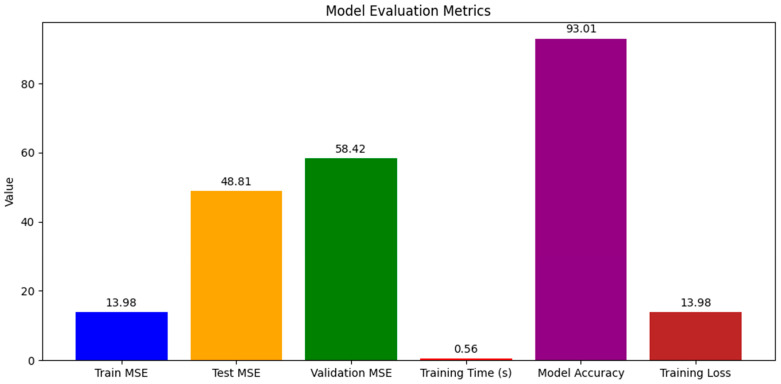
Model evaluation metrics for the recurrent neural network (RNN). Blue Bar: Training the mean squared error (MSE)—the average squared difference between estimated values and actual values for the training dataset. Orange bar: Test MSE—the average squared difference between predicted values and actual values for the test dataset. Green Bar: Validation MSE—the average squared difference between predicted values and actual values for the validation dataset. Red Bar: Training time(s)—the time in seconds that the model took to train. Purple Bar: Model accuracy—the percentage of accuracy derived from the model’s predictions. Brown Bar: Training loss—the loss calculated from the training dataset, which should correspond to the training MSE.

**Figure 3:**
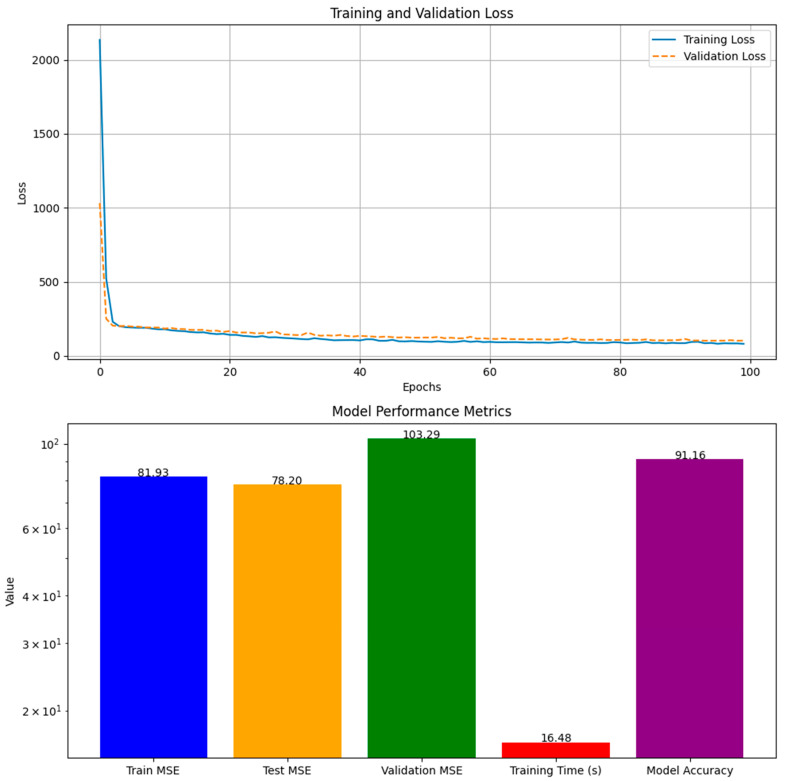
Training vs. validation loss and model performance metrics for the convolutional neural network (CNN). The solid blue line in the upper plot indicates the training loss across epochs, and the dashed orange line shows the validation loss, representing how the model’s performance improved with each epoch during the training and validation phases, respectively. In the lower bar chart, the blue bar represents the training mean squared error (MSE), the orange bar indicates the test MSE and the green bar shows the validation MSE, which are MSE values for the respective datasets, providing a measure of the model’s prediction error. The red bar denotes the training time in seconds, indicating the computational time taken for the model to train. The purple bar shows model accuracy, offering an approximation of the model’s performance in percentage terms based on the test MSE.

**Figure 4:**
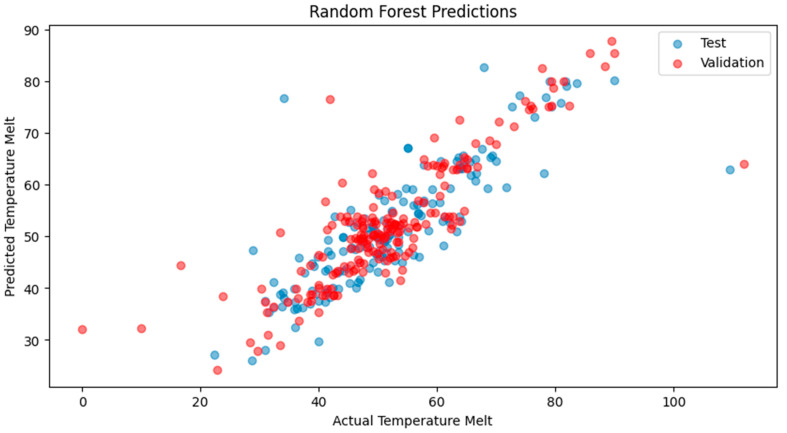
Scatterplot of predicted versus actual temperature melt values for the recurrent neural network (RNN) model, with blue dots representing test data predictions and red dots denoting validation data predictions.

Our explorations yielded nuanced results, indicative of the complex interplay between model architectures and the intricate nature of biological data. The RNN demonstrated a strong training fit with the RF regressor, with a training MSE of 13.98 and a high model accuracy of 93.01%, (Figure 2), yet the test and validation MSEs suggested overfitting, highlighting the need for model optimization. Despite the exceptional computational efficiency exhibited during training, the challenge remains to ensure that such efficiency translates into robust predictions across various datasets. The derived model accuracy, though ostensibly high, requires a circumspect interpretation in light of disparities with validation results. Turning to CNN, the training MSE suggested that the model might not be capturing the dataset’s intricacies to its full potential, perhaps calling for a reevaluation of the network’s depth or the introduction of a more diverse range of data points (Figure 3). Interestingly, the CNN showcased a marginally better performance on the test set, an indication that it may be adept at discerning broader patterns. The increase in the validation MSE, however, underscores a common thread in the field of machine learning—the quest for balance between model complexity and dataset comprehensiveness. These initial outcomes, particularly elevated MSE values of the validation sets, are reflective of the multifaceted nature of protein thermostability predictions. A scatterplot (Figure 4) further illustrates these points, where the alignment of the predicted temperature melt values from the RNN model with the actual values reveals the model’s learning effectiveness on the training set, but also highlights variance in predictions on the test and validation sets, underscoring the necessity for ongoing model refinement. They also underline potential constraints posed by the scale of the data at our disposal. In response to these challenges, we are poised to extend our dataset and enhance the sophistication of our model designs. Our commitment to this iterative process is driven by the recognition that each refinement brings us closer to a more accurate portrayal of the phenomena under investigation. With a continued focus on dataset expansion and model optimization, we maintain an optimistic outlook toward achieving more accurate and generalizable models for enzyme thermostability, contributing valuable insights to the field.

The x-axis represents the “Importance” of each feature as determined by the random forest model, quantifying the relative contribution of each feature to the model’s predictive accuracy. The importance is scaled between 0 and 1, where higher values indicate a greater impact on model performance. The y-axis lists the “Features” analyzed in the study, such as pH, sulfur content (S), and molecular weight, among others. Each feature is assessed for its role in influencing the thermostability of laccases. Dark blue bars: These bars represent features with the highest importance scores in the random forest model, indicating that these features have the strongest relationship with the target variable. Light blue to gray bars: These bars show features with moderate importance scores. Beige bars: These bars indicate features with lower importance scores, suggesting a weaker relationship with the target variable.

### 2.2. Feature Importance

**Figure 5:**
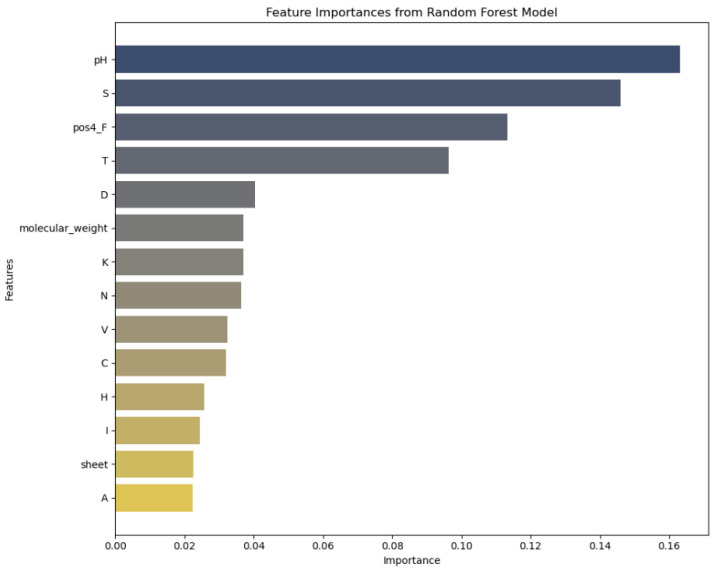
Relative importance of features in predicting laccase thermostability.

In analyzing feature importances of the RF model (Figure 5), it was evident that certain features had a more pronounced impact on predicting the thermostability of laccases. With the highest importance score, pH plays a pivotal role, which is biologically plausible given that enzyme stability is highly sensitive to pH levels. The 3D structures of proteins and, hence, their stability, can be affected by pH-induced changes in charge distributions and hydrogen bonding.

### 2.3. Determination of Structural Features of Laccases—The Catalytic Domain Architecture

**Figure 6:**
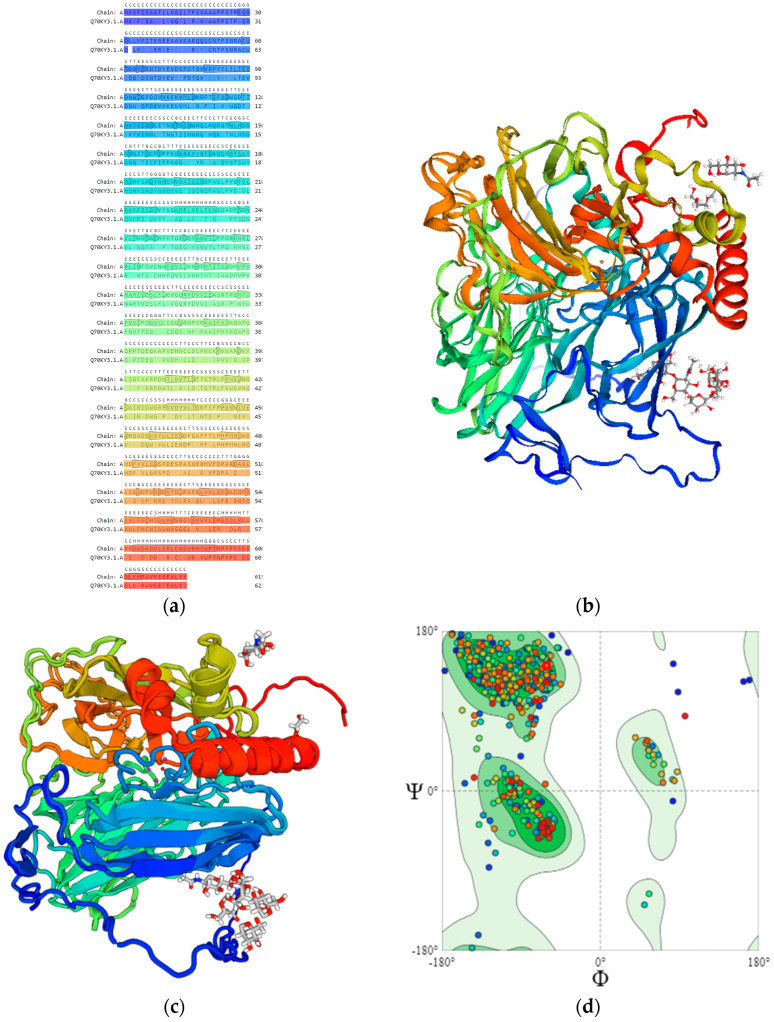
Amino acid profile, structure, and the Ramachandran plot of the laccase enzyme from Thermus thermophilus. The protein structure, visualized and edited using the software PyMOL (Version: 4.6.0 - Build 26.20.100.6911), showcases the enzyme’s domains in distinctive colors, with the catalytic domain highlighted, indicating areas of structural and functional significance. Homology modeling was employed, using SWISS-MODEL (https://swissmodel.expasy.org/ accessed on 14 April 2024) with a high sequence identity template, to elucidate the enzyme’s conformation. (**a**) Amino acid profile alignment: This panel displays the alignment of amino acid sequences from various thermostable laccase enzymes, highlighting the conserved residues. The alignment is color-coded to show amino acid properties, enhancing visualization of sequence conservation and variability across different species. (**b**) Three-dimensional (3D) structure of laccase enzyme: The three-dimensional structure of a laccase enzyme from Thermus thermophilus is shown, with distinct domains color-coded. The active site, crucial for the enzyme’s catalytic function, is highlighted in red, illustrating the spatial arrangement of key residues involved in substrate binding and electron transfer. (**c**) Structural model and domain organization: This diagram illustrates the domain organization within the laccase molecule. Each domain is represented in a different color, corresponding to the 3D structure in (**b**), to facilitate understanding of the structural layout and the functional segmentation of the enzyme. (**d**) Ramachandran plot: This plot presents the dihedral angles ψ (psi) and φ (phi) of the amino acid residues within the laccase enzyme. The plot identifies regions representing the most favored, allowed, and outlier conformations. Specific residues falling outside the favored regions are noted, which means thermophilic proteins constitute with higher number of beta sheets, left or right-handed helix.

Fungal laccases feature four catalytic copper atoms, particularly the T1 Cu and tri-nuclear Cu cluster (T2 Cu, T3α Cu, and T3β Cu) positioned at the T2/T3 site. Substrates undergo sequential one-electron oxidation at the T1 site proximal to the protein surface [8]. Meanwhile, the four-electron reduction of O_2_ to water takes place at the T2/T3 site (ligand-binding pocket), which is deeply embedded within the protein. These laccases are characterized as extracellular and monomeric glycoproteins, comprising approximately 520–550 amino acids with a typical weight of around 60–70 kDa in their glycosylated form [9]. They harbor an N-terminal signal peptide sequence spanning 20–22 residues. Structurally, they exhibit three tightly arranged cupredoxin-like domains, each featuring β-barrel symmetry. The T1 Cu is situated in domain 3, close to the protein surface, while T2 and T3 (α and β) Cu atoms reside at the domain interface [10] (Figure 6). The ligands depicted in (Figure 6b,c) represent the substrates or cofactors associated with the laccase enzyme. Specifically, these include oxygen molecules and other electron donors involved in the catalytic cycle of laccases. The ligands are integral to the enzymatic reaction, facilitating the transfer of electrons at the T1 copper site and subsequent reduction of oxygen to water at the T2/T3 trinuclear copper cluster [1,11,12].

Laccases belong to the multi-domain cupredoxin family, sharing ancestry with nitrite reductase, ascorbate oxidase, CueO, Fet3p, CotA, bilirubin oxidase, phenoxazinone synthase, ceruloplasmin, and coagulation factor V [13]. Multidomain cupredoxins evolved from a common ancestor, involving gene duplication or insertions for their structural complexity [14]. Common laccases typically have three cupredoxin domains forming a β-barrel structure. Recently, two-domain laccases with a homotrimeric arrangement were discovered, highlighting an evolutionary link between nitrite oxidases and multi-copper oxidases, supporting the existence of a common ancestor [15].

### 2.4. Evolutionary Aspects of Laccase Stability

Laccases are ancient enzymes that evolved early in evolutionary history and are now widespread across bacteria, fungi, plants, and animals. Primitive bacterial laccases appear to have evolved as highly stable enzymes [16] adapted to withstand extreme conditions, as evidenced by the thermostable bacterial laccases from *Thermus thermophilus* being able to retain activity at temperatures of up to 92 °C [17]. In contrast, laccases in eukaryotes later diversified and specialized, sacrificing some stability in exchange for regulatory control and novel functions. The high stability of bacterial laccases may be an evolutionary adaptation to harsh environments, while the lower stability of fungal and plant laccases reflects specialization for narrower pH and temperature ranges. Gene duplication events likely led to the sub-functionalization of paralogous laccase genes and the diversification of biochemical properties in different organisms. Differences in domain architectures between bacterial and eukaryotic laccases, with bacterial laccases having simpler two-domain structures (Table 1), also contribute to the higher stability of bacterial enzymes [18].

**Figure 7:**
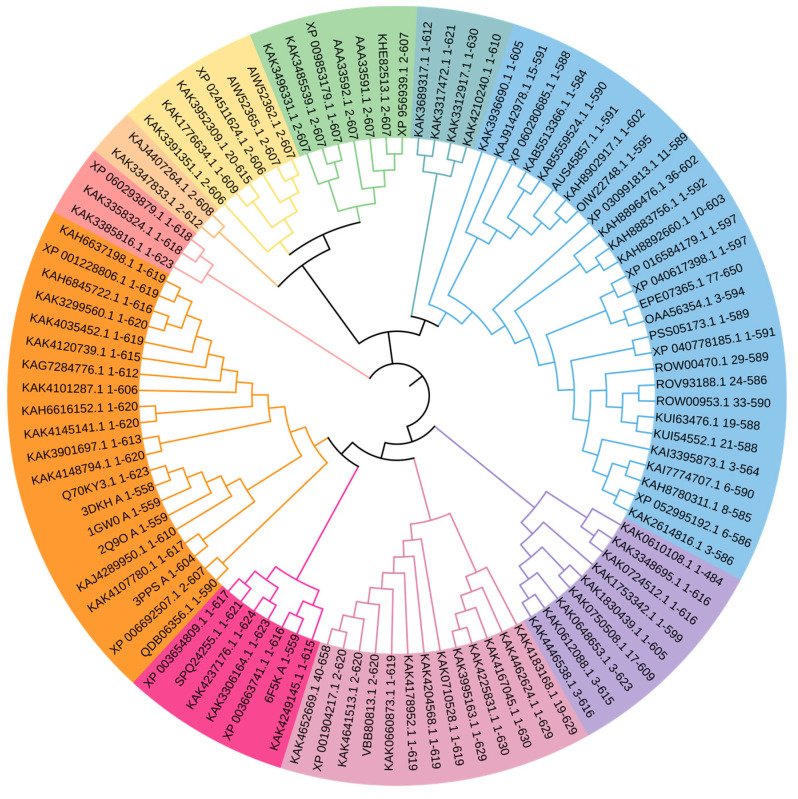

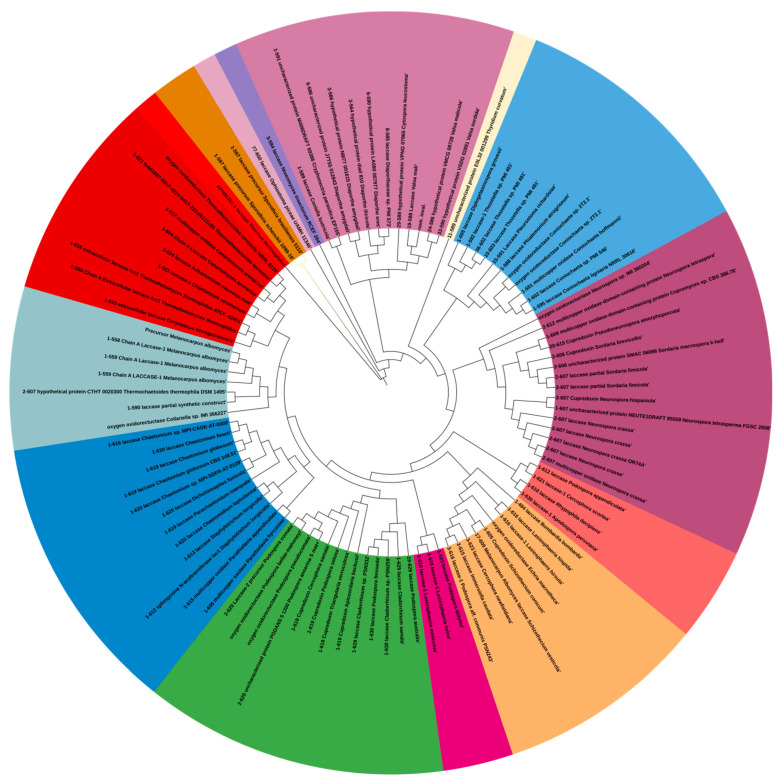
Phylogenetic tree of laccases and related genes from different domains (Supplementary Material File S2).

It appears that ancient bacterial laccases largely evolved as stable enzymes adapted to extreme conditions, while eukaryotic laccases later specialized, trading off some stability for regulatory control and novel functions (Figure 7).

### 2.5. Amino Acid Profiling

Research on a single amino acid bionanozyme designed as a mimic of laccase showed that the presence of acidic amino acids can contribute to stability under extreme conditions, including high temperatures [19]. Higher percentages of acidic amino acids were shown to significantly impact the thermostability of enzymes, influencing both their catalytic efficiency and stability. This effect was observed in studies on tetramer L-asparaginase II, for which modifications in the acidic amino acid composition were linked to enhanced enzyme performances [20]. Additionally, machine learning analyses underscored the importance of specific amino acid attributes, such as the Gln content and the presence of hydrophilic residues, including acidic amino acids, for the thermostability of laccase enzymes (Table 2), highlighting the complex interplay between the amino acid composition and enzyme stability [21]. Also, directed evolution and rational design approaches have demonstrated the potential for specific amino acid alterations, such as increasing acidic amino acids, to improve enzyme thermostability, offering pathways for the targeted enhancement of laccase stability for various applications.

**Table 1:** Domain analysis of laccases from various organisms as predicted by InterProScan [22] (https://www.ebi.ac.uk/interpro/about/interproscan accessed on 14 April 2024). The InterPro ID for each domain is provided.

Organism	Family	Domain	Homologous Superfamily	Conserved Site	Binding Site
*Thermothelomces thermophilus*	Multi-copper oxidase F Cu-oxidase_fam-IPR045087 multi-copper oxidase-PTHR11709	Cu-oxidase_CCu-oxidase_2 D Cu-oxidase_C-IPR011706 Cu-oxidase_2-PF07731	Cupredoxin H Cupredoxin-IPR008972 Cupredoxins-SSF49503	S Cu_oxidase_CS-IPR033138 MULTICOPPER_OXIDASE1-PS00079	S Cu_oxidase_Cu_BS-IPR002355 multicopper_oxidase2-PS00080
*Parachaetomium inaequale*	Multi-copper oxidase F Cu-oxidase_fam-IPR045087 multi-copper oxidase-PTHR11709	Cu-oxidase_2ndCu-oxidase D Cu-oxidase_2nd-IPR001117 Cu-oxidase-PF00394	Cupredoxin H Cupredoxin-IPR008972 Cupredoxins-SSF49503	S Cu_oxidase_CS-IPR033138 multicopper_oxidase1-PS00079	S Cu_oxidase_Cu_BS-IPR002355 multicopper_oxidase2-PS00080
*Parathielavia appendiculata*	Multi-copper oxidase F Cu-oxidase_fam-IPR045087 multi-copper oxidase-PTHR11709	Cu-oxidase_CCu-oxidase_2 D Cu-oxidase_C-IPR011706 Cu-oxidase_2-PF07731	Cupredoxin H Cupredoxin-IPR008972 Cupredoxins-SSF49503	S Cu_oxidase_CS-IPR033138 multicopper_oxidase1-PS00079	S Cu_oxidase_Cu_BS-IPR002355 multicopper_oxidase2-PS00080
*Chaetomium globosum* CBS 148.51	Multi-copper oxidase F Cu-oxidase_fam-IPR045087 multi-copper oxidase-PTHR11709	Cu-oxidase_2ndCu-oxidase D Cu-oxidase_2nd-IPR001117 Cu-oxidase-PF00394	Cupredoxin H Cupredoxin-IPR008972 Cupredoxins-SSF49503	S Cu_oxidase_CS-IPR033138 multicopper_oxidase1-PS00079	S Cu_oxidase_Cu_BS-IPR002355 multicopper_oxidase2-PS00080
*Corynascus novoguineensis*	Multi-copper oxidase F Cu-oxidase_fam-IPR045087 multi-copper oxidase-PTHR11709	Cu-oxidase_CCu-oxidase_2 D Cu-oxidase_C-IPR011706 Cu-oxidase_2-PF07731	Cupredoxin H Cupredoxin-IPR008972 Cupredoxins-SSF49503	S Cu_oxidase_CS-IPR033138 multicopper_oxidase1-PS00079	S Cu_oxidase_Cu_BS-IPR002355 multicopper_oxidase2-PS00080
*Chaetomidium leptoderma*	Multi-copper oxidase F Cu-oxidase_fam-IPR045087 multi-copper oxidase-PTHR11709	Cu-oxidase-like…Cu-oxidase_3 D Cu-oxidase-like_N-IPR011707 Cu-oxidase_3-PF07732	Cupredoxin H Cupredoxin-IPR008972 Cupredoxins-SSF49503	S Cu_oxidase_CS-IPR033138 multicopper_oxidase1-PS00079	S Cu_oxidase_Cu_BS-IPR002355 multicopper_oxidase2-PS00080
*Cladorrhinum samala*	Multi-copper oxidase F Cu-oxidase_fam-IPR045087 multi-copper oxidase-PTHR11709	Cu-oxidase_2ndCu-oxidase D Cu-oxidase_2nd-IPR001117 Cu-oxidase-PF00394	Cupredoxin H Cupredoxin-IPR008972 Cupredoxins-SSF49503	S Cu_oxidase_CS-IPR033138 multicopper_oxidase1-PS00079	S Cu_oxidase_Cu_BS-IPR002355 multicopper_oxidase2-PS00080
*Staphylotrichum longicolle*	Multi-copper oxidase F Cu-oxidase_fam-IPR045087 multi-copper oxidase-PTHR11709	Cu-oxidase-like_NCu-oxidase_3 D Cu-oxidase-like_N-IPR011707 Cu-oxidase_3-PF07732	Cupredoxin H Cupredoxin-IPR008972 Cupredoxins-SSF49503	S Cu_oxidase_CS-IPR033138 multicopper_oxidase1-PS00079	S Cu_oxidase_Cu_BS-IPR002355 multicopper_oxidase2-PS00080

Cu-oxidase-like…Cu-oxidase_3″ refers to a range of copper oxidase domains identified by InterProScan.

## 3. Discussion

Our study unveils a nuanced understanding of laccase thermostability, emphasizing the synergy between advanced computational models and biological insights. The predictive capabilities of RNNs and CNNs have delineated the complex interplay of genomic features influencing enzyme stability. In particular, features like pH, sulfur contents, specific amino acids (such as phenylalanine and threonine), molecular weights, and beta-sheet contents have emerged as significant predictors of thermostability. These findings suggest a multifactorial model of enzyme stability, wherein primary sequence attributes and higher-order structural characteristics both coalesce to dictate thermostability. Biologically, the prominence of pH and specific amino acids underscores the critical role of the enzyme’s microenvironment and amino acid composition in stabilizing proteins at elevated temperatures. We emphasize the importance of specific amino acids cysteine, threonine, and phenylalanine in enhancing the thermostability of laccases. Cysteine may contribute through its ability to form disulfide bonds, stabilizing protein structures at high temperatures. However, experimental validation of these claims is required. Threonine can help stability via hydrogen bonding, and phenylalanine through hydrophobic interactions Additionally, previous studies have shown that certain amino acids like arginine, threonine, alanine, and glutamic acid contribute significantly to thermostability (Table 2) by long-range interactions [23]. For instance, the importance of sulfur may reflect the stabilizing effects of disulfide bonds, while the relevance of beta-sheet contents highlights structural configurations conducive to thermal resilience. These insights align with the concept that thermostability is not merely a function of individual amino acids but also of the complex structural and environmental contexts within which these biomolecules operate.

Despite the promising strides, our study encountered several limitations. Data availability remains a perennial challenge, with the diversity and representativeness of the dataset directly influencing model performance and generalizability. The potential for model biases, especially in capturing the multifaceted nature of protein stability, necessitates a cautious interpretation of the results. Additionally, computational constraints, including the scalability of models to accommodate larger, more complex datasets, present ongoing challenges.

Looking forward, there is substantial room for enhancing the predictive modeling of enzyme thermostability. Incremental advancements could stem from expanding the dataset and integrating more diverse and comprehensive genomic data to enrich model training and validation. Exploring other machine learning algorithms, particularly those that are adept at handling the idiosyncrasies of biological data, could unveil novel insights into enzyme stability mechanisms. For instance, Transformer-based models, renowned for their prowess in capturing long-range dependencies in sequence data, may offer superior predictive performances [24]. Also, there is a need for experimental validation to support our computational predictions and provide a clearer understanding of the enzyme’s catalytic mechanism under different thermal conditions.

Moreover, extending the application of our computational framework to other enzymes could illuminate universal and enzyme-specific determinants of thermostability, fostering a broader understanding of protein behaviors across diverse biological contexts [25]. Such endeavors could significantly contribute to the biotechnological engineering of enzymes, tailoring their stability profiles to meet industrial demands, thereby advancing the frontier of sustainable and efficient biocatalysis. In essence, our study lays the groundwork for future explorations into the thermostability of laccases and related enzymes, advocating for a holistic approach that bridges computational innovations with deep biological understanding. Through iterative enhancements in computational models and data curation, coupled with an expanded focus on diverse enzymes, we aim to unravel the intricacies of enzyme thermostability, paving the way for novel biotechnological applications.

## 4. Materials and Methods

### 4.1. Data Collection

The genomic data central to our study were meticulously curated from several publicly accessible bioinformatics databases, including the National Center for Biotechnology Information (NCBI) GenBank [26] (https://www.ncbi.nlm.nih.gov/genbank/ accessed on 14 April 2024), UniProtKB [27] (https://www.uniprot.org/ accessed on 14 April 2024), and the Joint Genome Institute (JGI) MycoCosm portal [28] (https://mycocosm.jgi.doe.gov/mycocosm/home accessed on 14 April 2024). These repositories are renowned for their comprehensive collections of genomic and protein sequences, offering a robust foundation for our analysis. Upon collection, the sequences underwent a standardized preprocessing protocol. This included removing non-coding regions, correcting alignments, and normalizing data formats. Finally, we utilized a dataset comprising 926 unique enzyme instances. Features relevant to thermostability, such as amino acid compositions, sequence lengths, and specific motifs known to influence enzyme stability, were meticulously extracted, and encoded for model training.

The selection criteria were as follows:

Organism type: We specifically targeted genes from both fungal and bacterial sources, given their prevalent roles in laccase production. Preference was given to sequences from well-studied species with documented relevance to industrial applications.

Sequence quality: Only sequences with complete coding regions and high-quality annotations were included to ensure the reliability of our predictive models. This included sequences with confirmed functional assays when available.

Thermostability information: Sequences were prioritized based on the availability of experimental data regarding their temperature stability, allowing us to construct a dataset with a wide range of thermostabilities. This criterion was crucial for training our models to accurately predict thermostability from genomic features.

Diversity: To ensure the robustness of our model, we aimed for a dataset representing a wide phylogenetic spectrum. This diversity helped in capturing the vast genetic variability influencing laccase thermostability across different organisms.

### 4.2. Feature Extraction and Preprocessing

The pivotal role of feature extraction and preprocessing in our research on the thermostability of laccases is exemplified through the development and utilization of Python-based software (Version 3.10), “Laccase Thermostability Analyzer” (Supplementary Material File S1). This software leverages the rich ecosystem of bioinformatics libraries available in Python, such as Biopython and Matplotlib, to perform detailed analyses and visualization of genomic data. The process involves several sophisticated steps, each designed to elucidate features critical to understanding and predicting enzyme stability under various conditions. The software was structured to facilitate the extraction of key features from raw genomic data, transforming this information into a format amenable to machine learning analyses. The software employs the SeqIO and pairwise2 modules from Biopython to respectively read and analyze protein sequences [29].

The feature extraction process was vital in transforming raw genomic data into a structured format amenable to machine learning analysis. This procedure involved identifying and quantifying characteristics believed to influence the thermostability of laccases and related genes. Given the multifaceted nature of protein stability, a comprehensive set of features was derived from protein sequences to capture various aspects of their structure and composition that were potentially correlated with thermostability.

#### 4.2.1. Amino Acid Composition

Each protein sequence was analyzed to determine the relative frequencies of 20 standard amino acids. This information is crucial, as the composition and distribution of amino acids within a protein can significantly affect its stability, folding, and functions at different temperatures [30].

#### 4.2.2. Molecular Weight

The molecular weight of each enzyme was calculated based on the amino acid sequence. Larger proteins may exhibit different stability characteristics compared to smaller proteins, partly due to the complexity of their folding patterns and surface area exposed to solvent interactions [31].

#### 4.2.3. Aromaticity

The aromaticity index was computed to assess the proportion of aromatic amino acids (phenylalanine, tyrosine, and tryptophan) within the protein. Aromatic residues are known for their stabilizing interactions, such as π-π stacking, which can contribute to a protein’s thermal stability [32].

#### 4.2.4. Isoelectric Point (pI)

The pI was calculated for each protein, reflecting the pH at which the protein carries no net electrical charge. The pI can influence protein stability and solubility, which are critical for enzymatic functions across different temperatures [33].

#### 4.2.5. Secondary Structural Content

The proportions of amino acids participating in α-helices, β-sheets, and turns were determined, providing insights into the secondary structure of the enzymes. The configuration and content of secondary structural elements are integral to protein stability, affecting how proteins fold and maintain their structure under thermal stress [34].

#### 4.2.6. Extraction Technique

Utilizing bioinformatics tools and algorithms, each feature was extracted from the sequence data provided in the dataset. The process involved the application of sequence analysis tools to calculate the properties, ensuring that each feature accurately reflected the physical and chemical characteristics of the protein that might influence its thermostability.

The extracted features served as input variables for our machine learning models, facilitating the investigation into how these molecular characteristics correlated with the thermostability of laccases. This comprehensive feature set aimed to enable the models to uncover patterns and relationships within the data that can predict thermostability with high accuracy, contributing valuable insights into the design and optimization of thermostable enzymes for industrial applications.

### 4.3. Machine Learning Models

**Figure 8:**
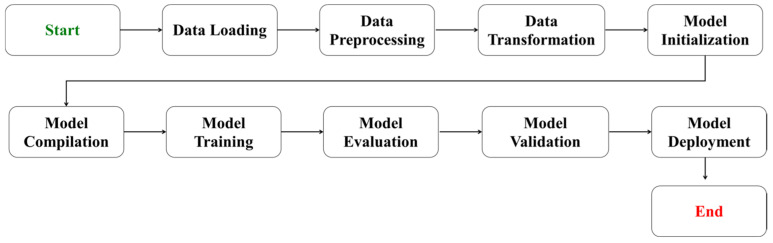
Brief layout of the machine learning process.

In our study, we employed two distinct modeling approaches to analyze and predict the thermostability of laccase enzymes: random forest (RF) regressors [35] and convolutional neural networks (CNNs) [36]. These methods were chosen for their ability to handle the complexity and non-linearity of biological data, as well as their proven track record in various predictive modeling tasks (Figure 8).

#### 4.3.1. RF Regressor

The preprocessing steps were as follows:

- The dataset was first split into training, test, and validation sets to ensure a robust evaluation of the model’s performance.
- Feature columns, excluding identifiers and the target variable (‘temp_melt’), were extracted as inputs for the model.

The model configuration was as follows:

- The RF model was initialized with 100 estimators, leveraging the ensemble learning method to reduce overfitting and improve prediction accuracy.
- The model was trained on the training set, with its performance evaluated on both the test and validation sets to gauge its generalization ability.

The rationale for selection included the following:

- RF was selected for its effectiveness in handling high-dimensional data, like amino acid sequences in our study, and its capacity to model complex relationships between features and the target variable without extensive parameter tuning.
- The model’s inherent feature importance metric offered insights into which genomic features were most predictive of thermostability, aiding in the biological interpretation of the results.

The mean squared error (MSE) was calculated by the following equation:

(1) $$\mathrm{MSE}=\frac{1}{n}\Sigma_{i=1}^{n}{\left(y_{i}-{\hat{y}}_{i}\right)}^{2}$$

where n is the number of observations, $y_{i}$ is the actual value, and ${\hat{y}}_{i}$ is the predicted value.

#### 4.3.2. Convolutional Neural Network (CNN)

The preprocessing steps were as follows:

- Input data were reshaped to fit CNN requirements, with each sequence represented as a two-dimensional (2D) array (features by 1) to mimic a single-channel image.
- Datasets were normalized to ensure efficient training of the neural network.

The model configuration included the following:

- Our CNN model comprised an input Conv1D layer with 64 filters and a kernel size of 3, followed by a MaxPooling1D layer to reduce dimensionality.
- A flattened layer was then applied to convert the pooled feature maps into a single vector per sample, which was followed by two Dense layers, with the final one outputting the predicted temperature melt value.
- The model was compiled using the Adam optimizer and MSE loss function, trained over 100 epochs with a batch size determined by default settings.

The rationale for selection included the following:

- The CNN was chosen for its ability to capture local dependencies and patterns within sequential data, a common characteristic of genomic sequences affecting protein function and stability.
- This approach is particularly suited for datasets like ours, where the spatial arrangement of features (e.g., amino acid sequences) plays a crucial role in determining the target variable (thermostability).

#### 4.3.3. Model Evaluation

Both models were rigorously evaluated using the MSE (Equation (1)) as the primary metric to quantify the prediction accuracy. Additional metrics, including model accuracy (approximated from the MSE for regression tasks) and training time, were also recorded to assess the performance and computational efficiency. A comparative analysis of model performances was conducted to identify which approach better captured the underlying patterns associated with laccase thermostability, providing a comprehensive view of the predictive capabilities of each model. These machine learning models, with their distinct yet complementary strengths, formed the core of our analytical framework, enabling us to delve deeply into the predictive modeling of thermostability in laccases and related genes. Through this exploration, we aimed to uncover new insights and contribute to the advancement of biotechnological applications leveraging stable laccase enzymes.

### 4.4. Model Training and Validation

We employed both RF regressors and CNNs to address the intricate challenge of enzyme thermostability. This entailed a deliberate preparation of data, careful model construction, and diligent validation to ensure the precision of our predictions.

#### 4.4.1. Data Preparation

Our datasets, saved to detailed CSV files, underwent a thorough preparation process. We filtered out non-essential information like sequence identifiers and external data sources and focused on features directly tied to thermostability. Notably, we pre-partitioned our datasets for training, testing, and validation, setting the stage for a robust evaluation of our models’ generalizability.

#### 4.4.2. Model Training

With the RF approach, we leveraged the ensemble strength of 100 trees, aiming to capture subtleties within our feature set while mitigating overfitting. The CNN model was structured to suit the unique demands of sequence data, utilizing convolutional and pooling layers to discern patterns pivotal for predicting thermostability. Training extended over 100 epochs, closely monitored by our validation data.

#### 4.4.3. Validation and Evaluation

The MSE was at the forefront of our evaluation metrics, gauging the precision of our models against true values of enzyme thermostability. Additionally, we approximated the model accuracy from MSE values as a more comprehensible performance indicator and kept a keen eye on training times to gauge the computational efficiency.

#### 4.4.4. Model Assessment

In our evaluation of the random forest model, we employed graphical representations to analyze key performance metrics such as training losses and mean squared error (MSE) across different datasets. These visualizations provided clear insights into the model’s performance, highlighting its strong learning capabilities as evidenced by a low training MSE of approximately 13.98. This effectiveness in training, however, contrasted with its performance on unseen data, where the MSE increased to about 48.81 for the test set and 58.42 for the validation set. These figures indicate a need for improvement in the model’s ability to generalize beyond the training data. The RF model was not only quick to train but also showed high accuracy, reaching approximately 93.01%. Despite its robust training performance, the increase in MSE across test and validation datasets suggests areas for potential enhancement to increase its generalization capabilities.

## 5. Conclusions

This investigation into the predictive modeling of thermostability in laccases and related genes marks a significant advance at the intersection of computational biology and biotechnological engineering. Utilizing advanced machine learning techniques, specifically RF regressors, and CNNs, our study has shed light on the complex dynamics of enzyme stability. We have identified critical genomic features influencing thermostability, such as pH levels, sulfur content, amino acid composition, molecular weights, and secondary structural elements. These findings underscore the nuanced interplay between these features and enzyme stability, illustrating the potential of computational methods to decode complex biological phenomena.

Our results not only clarify the predictors of thermostability but also set the stage for the rational design of enzymes with improved stability profiles, crucial for industrial applications where enzyme robustness is essential. Despite these advancements, our exploration into enzyme thermostability modeling has exposed limitations related to data availability, representativeness, and inherent model biases. These challenges temper our achievements and outline the necessary steps for future research, including expanding datasets, integrating diverse genomic information, and exploring novel machine learning algorithms.

Looking forward, applying our computational framework to a broader range of enzymes holds the promise of deepening our understanding of protein behavior in various biological contexts. This study represents a step in the ongoing effort to leverage computational biology for biotechnological innovations, combining sophisticated computational techniques with profound biological insights to address complex challenges. As we refine our models and continue our research, we remain dedicated to advancing the sustainable and efficient use of enzymes in industry, unlocking the secrets of enzyme thermostability one computation at a time and paving the way for future biotechnological breakthroughs.

## Figures and Tables

**Table 2:** Amino acid profiling (%) of laccases from various organisms performed using the ExPASy ProtParam Tool (https://web.expasy.org/protparam/ accessed on 14 April 2024).

UniProtKB ID /PDB/GenBank Accession No.	Source of Laccase	Total Amino Acid Residues	Theoretical pI	% of Acidic Amino Acid Residues		% of Sulfur Amino Acids			C + F + T %	D + E	% of Basic Amino Acid Residues		R + K
				Aspartic Acid (D)	Glutamic Acid (E)	Cysteine (C)	Phenylalanine (F)	Threonine (T)			Arginine (R)	Lysine (K)	
XP_003663741	*Thermothelomyces thermophilus* ATCC 42464	616	5.28	7.3	4.1	1.1	3.4	7.0	11.5	70	4.7	2.8	46
6F5K	*Thermothelomyces thermophilus*	559	5.08	7.9	3.2	1.3	3.6	7.0	11.9	62	4.5	2.3	38
KAK4035452	*Parachaetomium inaequale*	619	6.33	6.0	2.6	1.1	4.5	7.4	13.0	53	5.3	2.1	46
KAK4120739	*Parathielavia appendiculata*	615	6.64	6.0	2.3	1.1	4.4	7.5	13.0	51	5.2	2.4	47
XP_001228806	*Chaetomium globosum CBS 148.51*	619	6.11	5.8	3.1	1.3	4.5	8.2	14.0	55	5.3	1.9	45
KAK4249145	*Corynascus novoguineensis*	615	5.14	7.6	4.4	1.1	3.6	6.7	11.4	74	4.7	2.6	45
KAK4148794	*Chaetomidium leptoderma*	620	6.76	5.5	2.4	1.3	4.7	8.4	14.4	49	4.8	2.6	46
KAK4462624	*Cladorrhinum samala*	629	6.92	6.0	2.2	1.1	3.7	7.6	12.4	52	4.8	3.2	50
KAK3901697	*Staphylotrichum tortipilum*	613	6.46	5.5	2.4	1.3	4.9	8.5	14.7	51	4.4	3.1	46

pI, isoelectric point. “D + E” and “R + K” represent the actual number of aspartic acid (D) and glutamic acid (E) residues, and the number of arginine (R) and lysine (K) residues in the laccase.

## Data Availability

Data are available upon reasonable request.

## Supplementary Materials

The supporting information can be downloaded at https://www.mdpi.com/article/10.3390/ijms252313035/s1.
