# Supplementary material for: Precision Thermostability Predictions: Leveraging Machine Learning for Examining Laccases and Their Associated Genes
Source: Int J Mol Sci. 2024 Dec 4;25(23):13035. doi: 10.3390/ijms252313035 (PMC11641568; doi:10.3390/ijms252313035)
Supplement: Supplementary file 1 [file ijms-25-13035-s001.zip › file S2.pdf]

Laccase：

>AFN86157.1_laccase_Thermus_thermophilus
LTYGTLRVRPRDTVRLTLENRLPEPPFLEIPPGSWTYEFAVPKELAGTFWYHPHLHGRVAPQLFAGLLGALVV
ESSLDAI
PELREAEETPMDWMNGKEGDLLVNGALRPTLVAQKATLRLRLLNASNARYYRLALQDHPLYLIAADGFLEEPL
EVSELLL
APGERAEVLVRLRKEGRFLLQALPYDRGAMGMMDMGGMAAMPQGPSPETLLYLIAPKNPKPLPKLSPFPTLPA
PVVTRLV
LTEDMMAARFFINGQVFDHRRVDLKQVEVWEVENGDMDHPFHLHVHPFQVLSVGGPFPYAWKDVVNLKAGEVA
RLLVPLR
EKGRTVFHCHIVEHEDRGMMGVLEVG
>lcc1-1
MLVNTIFADWGDTIQVTVINNLETNITECPIPPGGRKVYRFKAQQYGTSWYHSHFSAQYGNGVVGAIQISLPY
DTDLGVF
PISDYYYSDNVLFNGTAKHPETGEGEYANVTLTPGRRHRLRLINTSVENHFQVSLVNHTMTIIAADMVPVNAM
TVDSLFL
GVGQRYDVVIEASRPGNYWFNVTFGNPYPAAIFHYAGAPGPPTDEGAPVDLPNLKPVVARDVLSFAKRPDNTL
DVTLAIN
IDWGRPVVDYVLTQNPPGYNIVEVNGWSYWLIENPGLPHPMHLHGHDFYVLGRSPESPAVRRDVTMLPAFGWV
VLAFRAD
NPGAWLFHCHIAWHVSGGLGVVYLER
>lcc1-2
MLVNTIFADWGDTIQVTVINNLETNITECPIPPGGRKVYRFKAQQYGTSWYHSHFSAQYGNGVVGAIQISLPY
DTDLGVF
PISDYYYSDNVLFNGTAKHPETGEGEYANVTLTPGRRHRLRLINTSVENHFQVSLVNHTMTIIAADMVPVNAM
TVDSLFL
GVGQRYDVVIEASRPGNYWFNVTFGNPYPAAIFHYAGAPGPPTDEGAPVDLPNLKPVVARDVLSFAKRPDNTL
DVTLAIN
IDWGRPVVDYVLTQNPPGYNIVEVNGWSYWLIENPGLPHPMHLHGHDFYVLGRSPESPAVRRDVTMLPAFGWV
VLAFRAD
NPGAWLFHCHIAWHVSGGLGVVYLER
>lcc1-3
MLINTIFANWGDTIQVTVINNLETNVTECPIPPGGRRVYRFLAQQYGTSWYHSHFSAQYGNGVVGTIQISLPY
DIDLGVF
PLMDYYYRDNVLFNGTAKHPTTGAGQYANVTLTPGKRHRLRIINTSTENHFQVSLVNHTMTVIASDMVPVNAF
TVDSLFL
AVGQRYDVTIDASRPGNYWFNVTFGNPSPAAIFHYAGAPGLPTDRGAPVDLPNLTPVVTRNVVSFVKKPSNTL
PVKLAVN
VDWGKPVLDYVMTQNPASDNIVQVDGWTYWLVENPDLPHPMHLHGHDFLVLGRSPVPPGVRRDVTMLPAKGWL
LLAFKTD
NPGAWLFHCHIAWHVSGGLSVDFLER
>lcc1-4
MLINTIFADWGDTIEVTVINNLDTNVTECPIPPGGQRVYRFKAQQYGTSWYHSHFSAQYGNGVVGTIQISLPY
DIDLGVF
PLMDYYYRDNVLFNGTAKHPITGVGEWANVTLTPGKRHRLRIINTSTENHFQVSLVNHSMTIIASDMVPVNAM
TVDSLFL
AVGQRYDVTIDANRPGNYWFNVTFGHPSPAAIFHYAGAPGLPTNQGPPTDLPNLTPVVTRNVVSFVKRPGNTL
PVTLAIN
VDWGKPILDYVMTQNPTSDNIVQVDGWTYWLVENPDLPHPMHLHGHDFLVLGRSPVSPAVRRDVTMLPPRGWL
LVAFRTD
NPGAWLFHCHIAWHVSGGLSVDFLER
>lcc1-5
MLINTVFADWGDTIEVTVINNLDTNVTECPIPPGGQRVYRFKAQQYGTSWYHSHFSAQYGNGVVGTIQISLPY
EIDLGVF
PLMDYYYRDNVLFNGTAKHPITGVGEWANVTLTPGKRHRLRIINTSTDNHFQVSLVNHSMTIIASDMVPVNAM
TVDSLFL
AVGQRYDVTIDANSPGNYWFNVTFGHPTPAAIFHYAGAPGLPTNQGPPTDLPNLTPVVTRNVVSFVKRPGNTL
PVTIAIN
VDWGKPILDYVMTQNPTSDNIVQVDGWTYWLVENPDLPHPMHLHGHDFLVLGRSPVPPAVRRDVTMLPAGGWL
LLAFRTD

NPGAWLFHCHIAWHVSGGLSVDFLER
>lcc1-6
MLINTIFADWGDTIQVTVINNLDTNVTECPIPPGGQRVYRFKAQQYGTSWYHSHFSAQYGNGVVGTIQISLPY
DIDLGVF
PLMDYYYADNVLFNGTAKHPTTGAGQWANVTLTPGKRHRLRIINTSTENHFQVSLVNHSMTVIASDMVPVNAM
TVDSLFL
AVGQRYDVTIDANRPGNYWFNVTFGNPTPAAIFHYAGAPGLPTNKGPPTDLPNLTPVVTRNVVSFVKRPGNTL
PVNISIN
VDWGKPVADYVMTQNPTSDNIVQVDGWTYWLVENPDLPHPMHLHGHDFLVLGRSPVSPAVRRDVTMLPPRGWL
LLAFRAD
NPGAWLFHCHIAWHVSGGLSVTFLER
>lcc1-7
MLVNTIFANWGDTIQVTVINNLITNVTECPIPPGGRKVYRFRAQQYGTSWYHSHFSAQYGNGVVGTIQISLPY
DIDLGVF
PLMDYYYKDNVLFNGTAKHPTTGAGQYANVTLTPGKRHRLRIINTSTENHFQVSLVNHTMTVIAADMVPVNAM
TVDSLFL
GVGQRYDVTIDASRPGNYWFNVTFGNPHPAAIFHYAGAPGLPTDRGPPVDLPNLTPVVTRNVVNFVKKPSNTL
PVNLAIN
VNWNKPVLEYVMTGNPASDNIVQVDGWTYWLVENPDLPHPMHLHGHDFLVLGRSPVPPGVRRDVAMLPARGWL
LLAFKTD
NPGAWLFHCHIAWHVSGGLSVDFLER
>lcc1-8
MLINTIFADWGDTIQVTVINNLETNLTECPIPPGGRKVYRFKAQQYGTSWYHSHFSAQYGNGVVGTIHISLPY
DIDLGVF
PLMDYYYRDNVLFNGSAKHPTTGAGQYANVTLTPGKRHRLRIINTSTENHFQVSLVDHKFTIIASDLVPVQAQ
TVDSLFL
AVGQRYDVTIDGNAPGNYWFNVTFGNPNPAAIFHYAGSSGLPTKQGVPVDLPNLTPVVQRTVVSFTKKADNTL
TVNIAIN
VDWNKPVLEYVMTQNPRSENIVQIDAWTYWLIENPNLPHPMHLHGHDFLVLGRSPVPPGVRRDVTILPAGGWL
LLAFKSD
NPGAWLFHCHIAWHVSGGLSVDFLER
>lcc1-9
MLINTIFADWGDTIQVTVINNLDTNVTECPIPPGGRRVYRFKAQQYGTSWYHSHFSAQYGNGVVGTIQISLPY
DIDLGVF
PLMDYYYADNVLFNGTAKHPTTGAGQWANVTLTPGKRHRLRIINTSTENHFQVSLVNHTMTVIASDMVPVNAM
TVDSLFL
AVGQRYDVTIDANRPGNYWFNVTFGNPTPAAIFHYAGAPGLPTNRGPPTDLPNLTPVVTRNVVSFVKRPGNTL
PVNIAIN
VDWGKPVADYVMTQNPTSDNIVQVDGWTYWLVENPDLPHPMHLHGHDFLVLGRSPVSPAVRRDVTMLPPRGWL
LLAFRAD
NPGAWLFHCHIAWHVSGGLSVTFLER
>lcc1-10
MLINTIFADWGDTIQVTVINNLETNLTECPIPPGGRKVYRFKAQQYGSSWYHSHFSAQYGNGVVGTIHISLPY
DIDLGVF
PLMDYYYRDNVLFNGSAKHPTTGAGQYANVTLTPGKRHRLRIINTSTENHFQVSLVDHKFTIIASDLVPVQAQ
TVDSLFL
AVGQRYDVTIDGNAPGNYWFNVTFGNPNPAAIFHYAGSSGLPTKQGVPVDLPNLTPVVQRTVVSFTKKADNTL
TVNIAIN
VDWNKPVLEYVMTQNPRSENIVQIDTWTYWLIENPNLPHPMHLHGHDFLVLGRSPVPPGVRRDVTILPAGGWL
LLAFKSD
NPGAWLFHCHIAWHVSGGLSVDFLER
>lcc1-11
MLVNTIYGDWGDTIEVTVINNLRTNITECPIPPGGRKVYRFRARQYGTSWYHSHFSAQYGNGVVGSIQISLPY
DIDLGVF
PITDYYYLDNVLFNGTAKHHETGDGEYATVTLTPGKRHRLRLINPSVENHFQVSLVNHTMTIIASDMVPVQAQ
TVDSLFL
GVGQRYDVVIDASSPGNYWFNVTFGHPNPAAIFHYAGAPALPTDEGPPVDLDNLTPVLERNVVNFVQNPGNTL
DISLAIN
IDWGKPAVDYVLTGNPREYNIVQVDGWTYWLIENPDLPHPMHLHGHDFYVLGRSPTSPGTRRDVAMVPARGWL
LIAFKTD
NPGAWLFHCHIAWHVSGGLSVTFLER
>lcc1-12

MLVNTIFADWGDTIQITVINKLETNVTECPIPPGGQRVYRFRAQQYGSSWYHSHFSAQYGNGVVGTILISLPY
EVDLGVF
PLMDYYYRDNVLFNGSAKHPTTGVGSYANVTLTPGKRHRLRIINTSTENHFQVSLVNHTMTVIASDMVPVNAM
TVDSLFL
AVGQRYDVTIDASRPGNYWFNVTFGNPFPAAIFHYAGALGLPTNKGTPTDRLDLTPVLTRTVASFVKKPSNTL
PVHLSMN
IDWGKPVLDYVMTQNQTGDNVVQVDGWTYWLVENPDLPHPMHLHGHDFFVLGRSPVSPAVRRDVTMLPPRGWL
LLAFKTT
NPGAWLFHCHIAWHVSGGLSVQFLER
>lcc1-13
MLVNTIRANWGDWIQVTVINNLRTNVTECPIPPGGQRVYRFRAAQYGTSWYHSHFSAQYGNGIVGTIQISLPY
DIDLGVF
PLSDYYYKDNVLFNGTGKHPVTGAGQYANVTLTPGKRHRLRIINTSTENHFQVSLVDHSMTVIASDMVPVNAM
TVDSLFL
AVGQRYDVTIDASKPGNYWFNVTFGNPNPAAIFHYAGAPGLPTKQGPPVDNMNLSPVVTRSAVSFVKKPGNTL
PVTLAIN
VDWQKPVLDYVMTQNPPGDNLVQIDSWTYWLVENPDLPHPMHLHGHDFLVLGRSPVPPAVRRDVTMLPAKGWL
LLAFKSD
NPGAWLFHCHIAWHVSGGLSVDFLER
>lcc1-14
MLVNTIFADWGDTIEVTVINNLETNITECPIPPGGTRVYRFRAQQYGTTWYHSHFSAQYGNGVVGTIQISLPY
DIDLGVF
PITDYYYRDNVLFNGTNVHPTTGAGSYANVTLTPGRRHRLRLINTSTENHFQLSLVNHSMTIIAADLVPVEAL
TVDSLFI
GVGQRYDVVIDASQPGNYWFNATFGNPTPAAIFHYEGAPGLPTDTGVPADNISLTPVVERNAVNFEKTPGNTL
DVHLAIN
VDWGKPVLDYVITGNPPSNNLVQVDEWTYWLIENPDLPHPMHLHGHDFLVLGRSPVPPAVRRDVAMLPAKGWL
LLAFRTD
NPGAWLFHCHIAWHVSGGLSVTFLER
>lcc1-15
MLINNIVANWGDTVEVTVINNLVTNVTECPIPPGGQRTYRWRARQYGTSWYHSHFSAQYGNGVVGTIQISLPY
DIDLGVF
PITDYYYRDNVLINGTAVNPNTGEGQYANVTLTPGKRHRLRILNTSTENHFQVSLVNHTMTVIAADMVPVNAM
TVDSLFL
AVGQRYDVVIDASRPDNYWFNVTFGNPHPAAIFHYAGAPGLPTDEGPPVDTLDVRPVVPRSVVNFVKRPDNTL
PVALDIN
VDWGKPIIDYILTGNPVSDNIVQVDAWTYWLIENPELPHPMHLHGHDFLVLGRSPVPAAPRRDTTMLPAGGWL
LLAFRTD
NPGAWLFHCHIAWHVSGGLSVDFLER
>lcc1-16
MLVNTIFADWGDWIEITVINNLRTNVTECPIPPGGTRKYRFRAQQYGSSWYHSHFSAQYGNGIVGTIQISLPY
DIDLGVF
PLTDYYYRDNVLINGTLKHPTSGAGSYATVNLTPGKRHRLRIINTSTENHFQVNLQNHTMTVISADFVPVNAM
TVDSLFL
GVGQRYDVTIDASRVGNYWFNITFGNPNPAAVFHYNGAPALPTNQGAPIDLPNLTPVVTRNVVSFVKKPQNTL
PVHLAVN
VDWNKPVLEYVMTQNPPGDNIVQVDAWVYWLVENPDLPHPMHLHGHDFVVLGRSPATPAVRRDVTMLPPKGWL
LLAFKTD
NPGAWLFHCHIAWHVSGGLSVDFLER
>lcc1-17
MLVNTILADWGDKIEVTVINNLKTNVTECAIPPGGKRVYKFRAQQYGTSWYHSHFSAQYGNGVVGAIQISLNY
DIDLGVF
PITDYYYRDNVLFNGTNVHPTTGAGRYANVTLTPGKRHRLRIINTSTENHFQLSLVNHSMTIIAADLVPVQAF
TVDSVFL
AVGQRLDVTIDASKPGNYWFNATFGNPSPAAIFHYAGAPGLPTDRGTPADNVNLTPVVKRTAVNFQKTPGNTL
DVHLTIN
VDWGKPVLDYVITGNPSSNNLVQVDEWTYWLIENPDLPHPMHLHGHDFLVLGRSPVPPAVRRDVAMLPAKGWL
LLAFRTD
NPGAWLFHCHIAWHVSGGLSVTFLER
>lcc1-18
MLVNTIVANWGDTIRVTVINNLLTNVTECPIPPGGRKVYQFRAQQYGTSWYHSHFSAQYGNGVVGTVQISRNY
DIDLGVF

PLMDYYHRDNVLFNGTAKHPMTGAGEYANVTLTPGKRHRLRLINTSTENHFQVSLVNHTMTVIAADMVPVQAQ
TVDSLFL
ALGQRYDVTIDAVSPGNYWFNVTYGNPHPAAVFHYAGGPGLPTDPGAPEDLNNLTPVVKRNVVSFVKKPSNTL
DVHLAIN
VDWNKPILEYVMTGNPVSDNIVQVDGWTYWLVENPDVPHPMHLHGHDFLVLGRSPVPPGVRRDVAMLPAKGWL
LLAFRTD
NPGAWLFHCHIAWHVSGGLSVNFLER
>lcc1-19
MLVNTIFADWGDNLEITVINNLQTNVTECPIPPGGKKLYRFRAVQYGTTWYHSHFSAQYGNGVVGGIQISLPY
DIDLGVF
PITDWYNLDNVLFNGTAVHPFTGEGKYANVTLTPGKRHRLRLINTSVENHFQVSLVNHTMTIIAADLVPVNAL
TVDSLFL
GVGQRYDVTIDASRPGNYWFNVTYGNKFPAAIFHYAGAPGLPTDPGAPVDNMDLTPVVKRSVANFVKKPENTL
DVHLAIN
IDWNNPVDDYVMKGNPTADNIVEVDAWTYWLIENPELPHPMHLHGHDFLVLGKSPSPPGVRRDVTMLPAKGWL
LLAFKTD
NPGAWLLHCHIAWHVSGGLSVVFLER
>lcc1-20
MLVNTIFADWGDNLEITVINNLQTNVTECPIPPGGKKLYRFRAVQYGTTWYHSHFSAQYGNGVVGGIQISLPY
DIDLGVF
PITDWYNLDNVLFNGTAVHPFTGEGKYANVTLTPGKRHRLRLINTSVENHFQVSLVNHTMTIIAADLVPVNAL
TVDSLFL
GVGQRYDVTIDASRPGNYWFNVTYGNKFPAAIFHYAGAPGLPTDPGAPVDNMDLTPVVKRSVANFVKKPENTL
DVHLAIN
IDWNNPVDDYVMKGNPTADNIVEVDAWTYWLIENPELPHPMHLHGHDFLVLGKSPSPPGVRRDVTMLPAKGWL
LLAFKTD
NPGAWLFHCHIAWHVSGGLSVVFLER
>lcc1-21
MLVNNIEANWGDTIQVNVINNLRTNVTDCPIPPGGSRVYRFRAQQYGTSWYHSHFSAQYGNGIVGTIVISLPY
DIDLGVF
PITDYYYKDNVLFKGLGKHPVTGAGQWANVTLTPGKRHRLRIINTSTENHFQVNLQNHTMTVIASDMVPVNAQ
TVDSLFL
AVGQRYDVTIDANKVGNYWFNVTFGHPNPAAIFHYAGAPGTPTHRGAPVDLPNLTPVVQRTVINFVKKPDNTL
PVHLAID
VDWGKPTVDYVLSQNPPQANVITVNSWTYWLIENPDLPHPMHLHGHDFVVLGRSPVPPGPRRDVTMLPAKGWV
LIAFKSD
NPGAWLFHCHIAWHVSGGLSVQYLER
>lcc1-22
MLVNNIEANWGDNIQITVINNLRTNITECPIPPGGSRVYKFRAQQYGTSWYHSHFSAQYGNGVVGTIVVSLPY
DIDLGAF
PLTDYYYRDNVLFKGLGKHPVTGAGQWANLTLTPGKRHRLRIINTSTENHFQVNLQNHTMTVIASDMVPVNAQ
TVDSLFL
AVGQRYDVTIDANKPGNYWFNVTFGNPAPAAIFHYAGAPGLPTNRGPPVDLDNLTPVVQRTVVNFVKKPDNTL
PVHLAID
VDWSKPTIDYVLSQNPPQANVITVNTWTYWLVENPDLPHPMHLHGHDFVVLGRSPVQPGTRRDVTMLPAKGWV
LIAFKSD
NPGAWLFHCHIAWHVSGGLSVQYLER
>lcc1-23
MLVNTIFADWGDFIQVTVINNLETNVTECPIPPGGRKVYRFRAQQYGTSWYHSHFSAQYGNGVVGTIHISLPY
DIDLGVF
PITDYYYKDNVLFNGTNVHPTTGAGQYANVTLTPGKRHRLRIINTSTENHFQLSLVNHSMTVISADMVPVNAM
TVDSLFL
AVGQRYDVTIDASKVGNYWFNVTFGHPAPAAVFRYAGAPVLPTDPGKPVDTVGLTPVVKRTVVNFVKKPSNTL
DVHLAIN
VNWGKPVLENVMAGNPREDNIVRVDGWTYWLVENPGIPHPMHLHGHDFLIVGRSPVAAGLRRDVTMLPAGGWL
LLAFKTD
NPGAWLFHCHIAWHVSGGLSVDFLER
>lcc1-24
MLVNTLEANWGDTFQINVINNLRTNVTDCPIPPGGSRIYRFRAQQYGTSWYHSHFSAQYGNGIVGTIVVSLPY
DIDLGAF
PITDYYYKDNVLFKGLGKHPVTGVGQYANITLTPGKRHRLRLINTSTENHFQLNLQNHSMTIIASDMVPVQAQ
TVNSVFL

AVGQRLDVTIDGNQIGNYWFNVTFGHPNPAAIFHYAGAPGVPTNRGAPVDLPNLTPVVSRTVVNFVKKVDNTL
PVTLAID
VDWQKPTVDFVLSQNPPEANVITVNSWTYWLIENPDLPHPMHLHGHDFVVLGRSPVQPGVRRDVTMLPAKGWL
LIAFKSD
NPGAWLFHCHIAWHVSGGLSVQYLER
>lcc1-25
MLVNTIEANWGDTIQVNVINNLRTNVTDCPIPPGGSRIYKFRAQQYGTSWYHSHFSAQYGNGVVGTIVVSLPY
DIDLGVL
PLTDYYYRDNVLIKGVAKHPVTGAGQWANITLTPGKRHRLRIINTSTENHFQVNLQNHTMTVIASDMVPVNAQ
TVDSLFL
AVGQRYDVTIDANKVSNYWFNVTFGNPSPAAIFHYAGAPGTPTNRGAPIDLPNLTPVVTRTVVTFVKKADNTL
PVHLAID
VDWGKPTVDYVLSQNPPQANVITVNAWTYWLIENPDLPHPMHLHGHDFVVLGRSPVQPAVRRDVTMLPAKGWL
LIAFKSD
NPGAWLFHCHIAWHVSGGLSVQYLER
>lcc1-26
MLINNIVANWGDTVEVTVINNLVTNVTECPIPPGGQRTYRWRARQYGTSWYHSHFSAQYGNGVVGTIQISLPY
DIDLGVF
PITDYYYRDNVLINGTAVNPNTGEGQYANVTLTPGKRHRLRILNTSTENHFQVSLVNHTMTVIAADMVPVNAM
TVDSLFL
AVGQRYDVVIDASRPDNYWFNVTFGNPHPAAIFHYAGAPGLPTDEGPPVDTLDVRPVVPRSVVNFVKRPDNTL
PVALDIN
VDWGKPIIDYILTGNPVSDNIVQVDAWTYWLIENPELPHPMHLHGHDFLVLGRSPVPAAPRRDTTMLPAGGWL
LLAFRTD
NPGAWLFHCHIAWHVSGGLSVDFLER
>lcc1-27
MLINNIVANWGDTVEVTVINNLVTNVTECPIPPGGQRTYRWRARQYGTSWYHSHFSAQYGNGVVGTIQISLPY
DIDLGVF
PITDYYYRDNVLINGTAVNPNTGEGQYANVTLTPGKRHRLRILNTSTENHFQVSLVNHTMTVIAADMVPVNAM
TVDSLFL
AVGQRYDVVIDASRPDNYWFNVTFGNPHPAAIFHYAGAPGLPTDEGPPVDTLDVRPVVPRSVVNFVKRPDNTL
PVALDIN
VDWGKPIIDYILTGNPVSDNIVQVDAWTYWLIENPELPHPMHLHGHDFLVLGRSPVPAAPRRDTTMLPAGGWL
LLAFRTD
NPGAWLFHCHIAWHVSGGLSVDFLER
>lcc1-28
MLINNIVANWGDTVEVTVINNLVTNVTECPIPPGGQRTYRWRARQYGTSWYHSHFSAQYGNGVVGTIQISLPY
DIDLGVF
PITDYYYRDNVLINGTAVNPNTGEGQYANVTLTPGKRHRLRILNTSTENHFQVSLVNHTMTVIAADMVPVNAM
TVDSLFL
AVGQRYDVVIDASRPDNYWFNVTFGNPHPAAIFHYAGAPGLPTDEGPPVDTLDVRPVVPRSVVNFVKRPDNTL
PVALDIN
VDWGKPIIDYILTGNPVSDNIVQVDAWTYWLIENPELPHPMHLHGHDFLVLGRSPVPAAPRRDTTMLPAGGWL
LLAFRTD
NPGAWLFHCHIAWHVSGGLSVDFLER
>lcc1-29
MLVNTILADWGDNIQVTVINNLRTNVTECPIPPGGTRVYKFKAQQYGTSWYHSHFSAQYGNGVVGTILISLPY
DIDLGVF
PITDYYYRDNVLFQGKGKHPVTGAGQWANVTLTPGKRHRLRIINTSTHDHFQVKLQNHTMTVIASDMVPVNAF
TVDSLFL
AVGQRYDVTIDANKVGNYWFNVTYGNPNPAAIFHYAGAPGVPTNRGPSIDLPNLTPVVTRTVVSFTKKADNTL
PVTLAIN
VQWEKPIIDYVLAQNPREANVITINSWTYWLIENPDLPHPMHLHGHDFVVLGRSPVNPAVRRDVTMLPARGWV
LIAFKSD
NPGAWLFHCHIAWHVSGGLSVDYLER
>lcc1-30
MLVNTIEANWGDWIEVNVINNLLTNVTECPIPPGGQRTYRFRAQQYGTSWYHSHFSAQYGNGIVGPIVISLPY
DIDLGPF
PLVDYYYKDNVLFNGTGVHPQTGHGQYAKVTLTPGKRHRLRIINMSTENHFQVSLVGHQFTVIAADMVPVHSY
NTDSLFL
AVGQRYDVIIDASPPGNYWFNVTFGNPHPAAIFHYEGAPALPTNPGTPRDTLDLVPVVPRNVVNFVKKPENTL
PVELAID

VDWGNPVLQYVMDGNRQADNIVEVNGWTYWLIENPNLPHPMHLHGHDFLIVGRSPVPPGTRRDVAMLPAGGWL
LLAFRTD
NPGAWLLHCHIAWHVSGGLSVDFLER
>lcc1-31
MLVNTITANWGDWIQVNVINNLRTNVTECPIPPGGSRIYKFRAQQYGTSWYHSHFSAQYGNGVVGTIVISLPY
DIDLGVF
PITDYYHKDTVLFKGQGKNPATGAGKFANITLTPGKRHRLRIINTSTHDHFQLKLQNHTMTIIAADMVPVQAQ
TVDSLFL
AVGQRYDVTIDANKVSNYWFNATFGNPHPAAIFRYQGAPANPTNQGPAADLNNLTPVVSRSVTAFTPRPNNTL
PVSLSIN
VDWDKPIVDYVIAQNPPQANVITVNTWTYWLVENPTIPHPMHLHGHDFLVVGRSPQPAGVRRDVAVLPANGWL
LIAFKSD
NPGAWLFHCHIAWHVSGGLSVQYLER
>lcc1-32
MLVNTIVADWGDFIQITVINNLRTNVTECPIPPGGSKIYKFRASQYGTSWYHSHFSAQYGNGVVGSMQISLPY
DIDLGVF
PITDYYYRDNVLFNGTNVHPVTGVGKYANVTLTPGKRHRLRLINPSTENHFQLSLVGHDMTIISSDLVPVNAM
TVSSVFL
GVGQRLDVTIDASKPGNYWFNVTFGNPTPAAIFHYAGAPGLPTNKGAPVDLLNLTPVVTRSVPGFNPNPGNTL
PVTIAIN
VDWNKPVLDYVLTGNPPRNNLIQVNSWTYWLIENDPLPHPIHLHGHDFLIVGRSPVTPGARRDVAMLPAKGWL
LIAFKTD
NPGAWLMHCHIAWHVSGGLSVDFLER
>lcc1-33
MLVNTIFADWGDNIEILVINNLRTNVTECPIPPGGRFTYKFRATQYGTSWYHSHFSAQYGNGVVGTIQISLPY
EVDLGVF
PISDYYYRDNVLFNGTAKHPTTGVGAYANVTLTPGKRHRLRIINTSTENHFQLSLVGHQFTIISSDFVPVQAQ
TVDSLFL
AVGQRYDVTIDASKPGNYWFNATFGNPAPAAIFHYAGAAGLPTDPGAPVDLLNLTPVVTRTVVAFSKRPANTL
PVTLAIN
VDWNKPVAEYILTSNPTSENIVQIDVWTYWLIENDPLPHPFHLHGHDFVILGRSPELPAARRDVVMLPARGWA
LIAFKSD
NPGAWLMHCHIAWHVSGGLSVDFLER
>lcc1-34
MLINTLVADWGDFFQITVINNLRTNVTECPIPPGGSKIYKFRAQQYGSSWYHSHFSAQYGNGVVGAIQISLPY
DIDLGPM
PLSDYYYRDNVLFNGFGKHPTTGAGQYANITLTPGKRHRLRLINTSTENHFQVSLANHTFTIIASDFVPVQAQ
TVDSVFL
GVGQRMDVTIDASKIGNYWLNVTFGNPAPAAIVHYAGASGLPTNPGAPVDLLNLTPVVQRTVPTFNKTPGNTM
PVTIAIN
VDWNKPIVDYVLTGNPTNENLVKIDAWTFWLIENDPLPHPIHLHGHDFVVLGRSPATPAMRRDVTMLPAKGWL
LIAFKTD
NPGSWLMHCHIAWHVSGGLSVDFLER
>lcc1-35
MLINTLVADWGDFFQITVINKLRTNVTECPIPPGGSKVYKFRAQQYGSSWYHSHFSAQYGNGVVGTIQISLPY
DIDLGPL
ALTDYYYRDNVLFNGFAKHPTTGAGQYANITLTPGKRHRLRLINTSTENHFQVSLANHTFTVIASDFVPVQAQ
TVDSVFL
GVGQRMDVTIDANKVGNYWLNVTFGNPAPAAVVRYAGAPGLPTFPGAPVDLINLTPVVQRTVPTFNKQPSNTL
PVTIAIN
INWNKPVVDYVLTGNPTSENLVQITAWTFWLIENDPLPHPIHLHGHDFVVLGRSPATPALRRDVAMLPAKGWM
LIAFKSD
NPGSWLMHCHIAWHVSGGLSVDFLER
>lcc1-36
MLVNTIRANWGDNIEVTVINNLKTNVTECPIPPGGRKTYKFRATQYGTSWYHSHFSAQYGNGVVGTIQISLPY
DIDLGVF
PLMDYYYRDNVLFNGTARHPETGAGQWYNVTLTPGKRHRLRIINTSTDNHFQVSLVGHNMTVIATDMVPVNAF
TVSSLFL
AVGQRYDVTIDANSVGNYWFNVTFGNRFPAAIFRYQGAPTLPTDQGPVPDNLNLTPVVTRSAVNFVKRPSNTL
GVTLAIN
VDWGKPILDYVMSGNPVSDNIVQVDAWTYWLIENPTLPHPMHLHGHDFLVLGRSPELPSVRRDVTMLPAGGWL
LLAFKTD
NPGAWLFHCHIAWHVSGGLSVDFLER

>lcc1-37
MLVNTITANWGDWIQVNVINNLRTNVTECPIPPGGSRIYRFRAQQYGTSWYHSHFSAQYGNGVVGTIVVSLPY
DIDLGVF
PITDYYHKDTVLFKGHGKNPQTGAGNFANVTLTPGKRHRLRIINTSTHDHFQLKLQNHTMTIIAADMVPVQAQ
TVDSLFL
AVGQRYDVTIDANKVGNYWFNATFGNPHPAAVFRYQGAPTLPTNIGPAADLNNLTPVVSRSVTSFTPRPNNTL
PVSLSIN
VDWDKPIVDYVIAQNPPQANVITVNSWTYWLIENPTIPHPMHLHGHDFLVVGRSPQQAGVRRDVAMLPANGWL
LIAFKSD
NPGAWLFHCHIAWHVSGGLSVQYLER
>lcc1-38
MLVNTITANWGDWIQVNVINNLRTNVTECPIPPGGSRIYRFRAQQYGTSWYHSHFSAQYGNGVVGTIVVSLPY
DIDLGVF
PITDYYHKDTVLFKGHGKNPQTGAGKFANVTLTPGKRHRLRIINTSTHDHFQLKLQNHTMTIIAADMVPVQAQ
TVDSLFL
AVGQRYDVTIDANQVGNYWFNATFGNPHPAAVFRYQGAPTLPTYIGPAADLNNLTPVVSRSVTSFTPRPNNTL
PVSLSIN
VDWDKPIVDYVIAQNPPQANVITVNSWTYWLIENPTIPHPMHLHGHDFLVVGRSPQPAGVRRDVAMLPANGWL
LIAFKSD
NPGAWLFHCHIAWHVSGGLSVQYLER
>lcc1-39
MLVNTITANWGDWIQVNVINNLRTNVTECPIPPGGSRIYRFRAQQYGTSWYHSHFSAQYGNGVVGTIVVSVPY
DIDLGVF
PITDYYHKDTVLFKGHGKNPQTGAGKFANVTLTPGKRHRLRIINTSTHDHFQLKLQNHTMTIIAADMVPVQAQ
TVDSLFL
AVGQRYDVTIDANKVGNYWFNATFGNPHPAAVFRYQGAPTLPTNIGPAADLNNLTPVVSRSVTSFTPRPNNTL
PVSLSIN
VDWDKPIVDYVIAQNPPQANVITVNSWTYWLIENPTIPHPMHLHGHDFLVVGRSPQPAGVRRDVAMLPANGWL
LIAFKSD
NPGAWLFHCHIAWHVSGGLSVQYLER
>lcc1-40
MLVNTITANWGDWIQVNVINNLRTNVTECPIPPGGSRIYRFRAQQYGTSWYHSHFSAQYGNGVVGTIVVSVPY
DIDLGVF
PITDYYHKDTVLFKGHGKNPQTGAGKFANVTLTPGKRHRLRIINTSTHDHFQLKLQNHTMTIIAADMVPVQAQ
TVDSLFL
AVGQRYDVTIDANKVGNYWFNATFGNPHPAAVFRYQGAPTLPTNIGPAADLNNLTPVVSRSVTSFTPRPNNTL
PVSLSIN
VDWDKPIVDYVIAQNPPQANVITVNSWTYWLIENPTIPHPMHLHGHDFLVVGRSPQPAGVRRDVAMLPANGWL
LIAFKSD
NPGAWLFHCHIAWHVSGGLSVQYLER
>lcc1-41
MLVNTITANWGDWIQVNVINNLRTNVTECPIPPGGSRVYRFRAQQYGTSWYHSHFSAQYGNGVVGTIVVSLPY
DIDLGVF
PITDYYHKDTVLFKGHGKNPATGAGQFANITLTPGKRHRLRIINTSTHDHFQLKLQNHTMTIIAADMVPVQAQ
TVDSLFL
AVGQRYDVTIDANKVGNYWFNATFGNPHPAAIFRYQGAPANPTNQGPAADLNNLTPVVTRSVTSFTPRPNNTL
PVSLSIN
VDWDKPIVDYVIAQNPPQANVITVNSWTYWLVENPTIPHPMHLHGHDFLVVGRSPQPAGVRRDVAMLPANGWL
LIAFKSD
NPGAWLFHCHIAWHVSGGLSVQYLER
>lcc1-42
MLVNTITANWGDWIQVNVINNLRTNVTECPIPPGASRVYRFRAQQYGTSWYHSHFSAQYGNGVVGTIVISLPY
DIDLGVF
PITDYYHKDTVLFKGHGKNPATGAGRFANVTLTPGKRHRLRIINTSTHDHFQLKLQNHTMTIIAADMVPVQAY
TVDSLFL
AVGQRYDVTIDANKVGNYWFNATFGNPHPAAIFRYQGAPANPTNQGPAADLNNLTPVVTRNVTSFTPRPNNTL
PVSLAIN
VDWDKPIVDYVIAQNPPQANVITVNSWTYWLVENPDIPHPMHLHGHDFLVVGRSPQPAGVRRDVAMLPANGWL
LIAFKSD
NPGAWLFHCHIAWHVSGGLSVQYLER
>lcc1-43
MLVNTITANWGDWIQVNVINNLRTNVTECPIPPGASRIYKFRAQQYGTSWYHSHFSAQYGNGVVGTIVVSLPY
DIDLGVF

PITDYYHKDTVLFKGHGKNAQTGGGKFANVTLTPGKRHRLRIINTSTHDHFQLKLQNHTMTIIAADMVPVQAQ
TVDSLFL
AVGQRYDVTIDANKVGNYWFNATFGNPHPAAVFRYQGAPTLPTNQGPAADLNNLTPVVTRNVTSFTPRPNNTL
PVSLSIN
VDWDKPIVDYVIAQNPPQANVITVNSWTYWLIENPTIPHPMHLHGHDFLVVGRSPQPAGVRRDVAMLPANGWL
LIAFKSD
NPGAWLFHCHIAWHVSGGLSVQYLER
>lcc1-44
MLVNTIFADWGDKIEILVINNLRSNVSECPIPPGGRFTYKFRATQYGTSWYHSHFSAQYGNGVVGTIQISLPY
EVDLGVF
PVSDYYYKDNVLFNGTGKHPVTGVGSYANVTLTPGKRHRLRLINTSTENHFQLSLVGHQFTIIAADLVPVQAQ
TVDSIFL
GVGQRYDVTIDASQPGNYWFNATFGNPAPAAIFHYAGAAGLPTNPGPPVDLLNLTPVLTRSVVAFVKRPANTL
PVTLAIN
VDWNKPVVDYILTGNPTSENIVQIDVWTYWLIENDPLPHPFHLHGHDFVVLGRSPELPATRRDVVMLPVRGWV
LIAFKTD
NPGAWLMHCHIAWHVSGGLSVDFLER
>lcc1-45
MLINTITADWGDRIQVTVINNLRTNLTECPIPPGGSKVYSFLATQYGTSWYHSHFSAQYGNGVVGTILISLPY
DIDLGTF
PLSDYYYESNVLFNGTNVHPVTGQGQYANVTLTPGKRHRLRLINTSVENHFQVSLVGHSMTIIASDLVPQNAM
TVQSLFI
AVGQRYDVTIDASQVGNYWFNVTFGNPTPAAIFHYAGASQLPTNPGAPADLLNLTPVVTRTVVAFAPQPNNTI
PIHLAIN
VQWEKPVIDYILTGNPAGENIIQVNSWAYWLIENDPLPHPIHLHGHDFVILGRSPATPAARRDVAMLPAKGWL
LLAFQTD
NPGAWLMHCHIAWHVSHGLSVDFLER
>lcc1-46
MLVNTIRANWGDNIEVTVINNLKTNVTECPIPPGGRKTYKFRATQYGTSWYHSHFSAQYGNGVVGTIQISLPY
DIDLGVF
PLMDYYYRDNVLFNGTARHPETGAGQWYNVTLTPGKRHRLRIINTSTDNHFQVSLVGHNMTVIATDMVPVNAF
TVSSLFL
AVGQRYDVTIDANSVGNYWFNVTFGNKFPAAIFRYQGAPTLPTDQGPVPDNLNLTPVVTRSAVNFVKRPSNTL
GVTLAIN
VDWGKPILDYVMSGNPVSDNIVQVDAWTYWLIENPTLPHPMHLHGHDFLVLGRSPELPSVRRDVTMLPAGGWL
LLAFKTD
NPGAWLFHCHIAWHVSGGLSVDFLER
>lcc1-47
MLVNTIVADWGDNIQVTVINNLVTNVTECPLPPGGSKVYKFRATQYGSSWYHSHFSAQYANGVVGTILISLPY
DIDLGVF
PLSDYYYQSNVLFNGTAKHPVTGAGSYANVTLTPGKRHRLRLINTSAENHFQVSLVGHSMTVIATDLVPVNAF
TTNSLLL
GVGQRYDVTIDASQPGNYWFNVTFGNPFPAAIFHYAGAPGLPTAQGTATDNLNFRPVVQRNVVSFTKRPANTL
PVTLSIN
VDWNKPVAEYVLTGNPTSENLVILDQWTYWLIENDPLPHPFHLHGHDFLVLGRSPVTPAVRRDVAMLPAGGWL
LIAFHTN
NPGAWLMHCHIAWHVSGGLSVDFLER
>lcc1-48
MLVNTIVADWGDNIQVTVINNLVTNVTECPIPPGGSKVYKFRATQYGTSWYHSHFSAQYANGVVGTILISLPY
DIDLGVF
PLSDYYYQSNVLFNGTAKHPVTGAGSYANVTLTPGKRHRLRLINTSAENHFQVALVGHSMTVIATDLVPVNAF
TTNSLLL
GVGQRYDVTIDASQPGNYWFNVTFGNPFPAAIFHYAGAPGLPTAKGTATDNLNFKPVVQRNVVSFTKRPANTL
PVTLSIN
VDWNKPVAEYVLTGNPTSENLVILDQWTYWLIENDPLPHPFHLHGHDFLVLGRSPVTPAVRRDVAMLPTGGWL
LIAFQTN
NPGAWLMHCHIAWHVSGGLSVDFLER
>lcc1-49
MLINTITADWGDTIQVTVINNLVTNVTECPIPPGGTHVYRFLATQYGTSWYHSHFSAQYGNGVVGPIVISLPY
DIDLGPL
ALSDYYYKDNVLFNGLAKHPITGAGNYATLTLTPGKRHRLRIINTSTENHFQVSLVNHSMTIIASDLVPVNAQ
TVSSLFL

AVGQRYDVTIDANQVGNYWLNVTFGNPNPAAIIKYTGAPGLPTNKGPVTDLQNLSPVLTRSLVSFTKRPANTL
PVTLAIN
VDWNKPVVDYVASGNPTSENIVVVPDWTYWLIENDPLPHPIHLHGHDFLVLGRSPIQPLARRDVTMLPAGGWL
LIAFKTD
NPGAWLMHCHIAWHVSGGLSVDFLER
>lcc1-50
MLVNTIFADWGDNIEVTVINNLRTNVTQCPIPPGGSMVYKFRATQYGTSWYHSHFSAQYSNGVVGTIQISLPY
DIDLGVF
PISDYYLKDNVFLNGTGKHPTTGQGQYANVTLTPGKRHRLRLINTSAENHFQLSLVGHQMTIIASDFVPVNAQ
TVSSVFL
GVGQRYDVTIDASQPGNYWFNVTFGNPAPAAIFHYAGAPGLPTNPGPPTDIINLTPVVTRTVTVFVKRPSNTL
PVHLAIN
VDWNKPVAEYVMTNNPTSENLVIVDAWTYWLVENDPLPHPFHLHGHDFVVLGRSPVTPATRRDVTMLPARGWI
LIAFKTN
NPGAWLMHCHIAWHVSGGLSVDFLER
>lcc1-51
MLVNTIMADWGDFIQITVINNMISNVTECPVPPGKSKVYRFHATQHGTSWYHSHFSAQYGNGIVGSIQISLPY
DVDLGVF
PISDYYYRDNILFNGTAKHPTTGAGKWYNVTLTPGKRHRLRIINPSAENHYQVSLVGHKFTIIASDLVPVNAQ
TVDDLFL
GVGQRYDVTIDANQVGNYWFNVTFGNPNPAAVFRYAGAPTLPTNQGPPTDLINLTPVVSRTIPSFSPTPGNTL
PVVFAIN
VDWNKPVLEYVITGNPPAENLVHINQWVYWLIENDPLPHPIHLHGHDFLIVGRSPSPPGGRRDVAMLPARGWM
LIAFKTD
NPGAWLMHCHIAWHVSGGLSVDFLER
>lcc1-52
MLVNTIFADWGDRVNITVTNNLRTNVTECPIPPGGSKVYSFMATQYGTSWYHSHFSAQYGNGVVGTIQISLPY
DVDLGVF
PITDYYYQDNILINGTNVHPTTGVGKYANVTLTPGKRHRLRIINTSVENHFQVSLVNHSMTVIAADMVPVNAY
TTDTLFL
GVGQRYDVTIDASKVGNYWFNVTLPNQFPAAVFHYAGASGLPTARGPPVDNIDLVPVVSRTAPGFNPSPNNSL
PVHLSIN
VDWNKPVLQYVIENNPPSENIVSVPSWSYWLIENDVLPHPFHLHGHDFLVLGRSPASPAVRRDVAMLPAKGWL
LIAFKTD
NPGAWLMHCHIAWHVSGGLSVDFLEG
>lcc1-53
MLVNTIVADWGDRISITVINNLKTNVTECPIPPGGSKVYNFLATQYGTSWYHSHFSAQYGNGIAGPIQISLPY
DIDLGTF
PITDYYYKDNVLFNGTNIHPITGTGKYSNVTLTPGKRHRLRLINTSVENHFQVSLVNHTMTVIAADFVPVNAY
TVDSLFL
GIGQRYDVTLDASKIDKYWFNVTFGNPKPAAIFHYVGASGRPTSPGDPVDLINLTPVITRAVTSFIPDAGNTL
PVTLSIN
VDWTKPVVEYILEGNPASENIVSVDQWVYWLISNDPLPHPIHLHGHDFVVLGRSPATPATRRDVTMLPAKGWV
LVAFKTD
NPGAWLLHCHIAWHASAGLGVDFLER
>lcc1-54
MLVNTILADWGDFIEVKVINNLLSNVTECPIPPGGTFTYKFRATQYGSSWYHSHFSAQYGNGVVGTIQISLPY
DIDLGVF
PVTDYYYADNVLFNGTNKHPVTGAGNYAEVKLTPGKRHRLRLINTSTENHFQLSLVGHQMTIIAADFVPVNAM
TVQSVFL
AVGQRYDVTIDASAPGNYWFNATFGNPAPAAIFRYTNSPALPTNPGRPTDILNLTPVVQRTVVQFVKRPANTL
PVQLAID
VNWSKPVAQYVIEGNPTQDNIVHVDEWTYWLIENDPLPHPFHLHGHDFVVLGRSPATPAVRRDVAMLPARGWL
LIAFKTN
NPGAWLMHCHIAWHVSHGLSVDFLER
>lcc1-55
MLINTIFANWGDTIEVTVNNHLRTNVTECPIPPGGSRVYSFRARQYGTSWYHSHFSAQYGNGVSGAIQISLPY
DIDLGVL
PLQDWYYKDNVLINGTAKHPITGEGEYAVVKLTPGKRHRLRLINMSVENHFQVSLAKHTMTVIAADMVPVNAM
TVDSLFM
AVGQRYDVTIDASQVGNYWFNITFGNPAPAAIFRYEGAPALPTDPGAPKDTLDLSPVVQKNVVDFVKEPGNTL
PVTLAAD

VDWDRPVLEYVMNNDPAKNNIVRVDGWTYWLVENPELPHPMHLHGHDFFVLGRSPVSPDVRRDVTMLPARGWL
LLAFRTD
NPGAWLFHCHIAWHVSGGFSVDFLER
>lcc1-56
MLVNTIRADWGDWIEVKVINNLRTNVTECPIPPGGQFTYRFRATQYGTSWYHSHFSAQYGNGVVGTIQISLPY
DIDLGVF
PITDYYYADNVLFNGTNVHPVTGAGKYAEVKLTPGKRHRLRLINPSTENHFQLSLVGHQMTIIAADFVPVNAM
TVQSVFL
GVGQRYDVTIDASAPGNYWFNATFGNPAPAAIFRYTNSPALPTNPGPPVDILNLSPVVQRNVVQFVKRPANTL
DVELAID
VDWSKPVAQYVIEGNPTQDNIVHVNEWTYWLIENDPLPHPFHLHGHDFVVLGRSPATPAVRRDVTMLPAKGWV
LIAFKTN
NPGAWLMHCHIAWHVSHGLSVDFLER
>lcc1-57
MLVNTIFADWGDRINITVTNNLRTNVTECPIPPGGSKVYSFVATQYGTSWYHSHFSAQYGNGVVGTIQISLPY
DIDLGVF
PITDYYYQDNILINGTNVHPVTGAGKYANVTLTPGKRHRLRLINTSVENHFQVSLVNHSMTVIAADMVPVNSY
TTDTLFL
GVGQRYDVTIDASKVGNYWFNVTLPHPAPAAIFHYAGASGLPTAPGPPVDNTDLVPVVSRTAPGFNPSPNNSL
PVHLSIN
VNWNKPVLQYVIEGNPPSENIVSVASWSYWLIENDILPHPFHLHGHDFLVLGRSPATPATRRDVTMLPAKGWL
LIAFKTD
NPGAWLMHCHIAWHVSGGLSVDFLEG
>lcc1-58
MMVNTLQADWGDYLEITVINRLKSNVTECPIPPGGRKVYRFRATQYGTSWYHSHFSAQYGNGIVGSLVISANY
DVDLGPF
PLMDYYYEDNVLFNGFAKHPTTGAGQYATVSLTKGKKHRLRLINTSVENHFQLSLVNHSMTIIAADLVPVQPK
KVDSLML
GVGQRYDVIIDANQVGNYWFNVTFGNPAPAAIFRYQGAPTLPTNKGAPPDLVDLTPVLQRSMTNIALNTGNKI
PITLAIN
VNWNKPVLEYVMTGNSESDNIVHIDGWKYWLIENPDLPHPIHLHGHDFLILGRSPVAAITRRDVAMLPAKGWL
LIAFRTD
NPGAWLMHCHIAWHVSGGLSNQFLER
>lcc1-59
MLVNTIFADWGDRINITVTNNLRTNVTECPIPPGGSKVYSFIAAQYGTSWYHSHFSAQYANGVVGSIQISLPY
DIDLGVF
PITDYYYRDNVLINGTNVHPVTGAGKYANVTLTPGKRHRLRLINPSVENHFQVSLVNHSMTVIAADLVPVNAY
TTNTLFL
GVGQRYDVTIDASKIGNYWFNVTLPHPAPAAIFHYAGAPGLPTNPGPPVDNTDLVPVVSRTAPGFNPNPNNSL
PVHLSIN
VNWNKPVLQYVIEGNPPSENIISVPSYSYWLIENDLLPHPFHLHGHDFLILGRSPASPVTRRDVTMLPAKGWL
LIAFRTD
NPGAWLMHCHIAWHVSGGLSVDFLEG
>lcc1-60
MMVNTLQANWGDYLEITVINRLKTNVTECPIPPGGSKVYRFRATQYGTSWYHSHFSAQYGNGIVGSIVISANY
DVDLGPF
PLMDYYYKDNVLFNGFAKHPTTGAGQYATVSLTKGKKHRLRLINTSVENHFQLSLVNHSMTIISADLVPVQPY
KVDSLLL
GVGQRYDVIIDANQVGNYWFNVTFGNPSPAAIFRYQGAPALPTNQGAPLDLNDLKPVLQRSLTNIALNTGNTI
PIKLAIN
VDWNKPVLEYVLTGNPQSENIVQIDGWKYWLIENPDLPHPIHLHGHDFLILGRSPVDPATRRDVAMLPARGWL
LIAFRTD
NPGAWLMHCHIAWHVSGGLSNQFLER
>lcc1-61
MLVNTILADWGDWIEVTVNNKLDTNVTECPIPPGHSFKYRFRAEQYGSSWYHSHFSAQYGNGVVGTIQISLPY
EVDLGVF
PISDYYYKDNVFFNGTNVHPTSGGGQYSVTTLQPGKRHRLRIINTSTENHFQLSLVGHQMTIIAADFVPVNAM
TVSSVFL
AVGQRFDVTIDASQPGNYWFNATFGNPTPAAIFRYAGAPTLPTNPGRPTDILNLSPVVQRTVITFTPRPANTL
KVTLAIN
VNWEKPVAQYVMESNPTSDNIVHVNDWTYWLVENDPLPHPFHLHGHDFVVLGRSPATPAVRRDVTMLPAKGWI
VIAFKTN
NPGAWLMHCHIAWHVSHGLSVDFLER

>lcc1-62
MLINTIFADWGDRINITVTNNLRTNVTECPIPPGGSKVYSFVATQYGTSWYHSHFSAQYGNGVVGTIQISLPY
DIDLGVF
PITDYYYRDNILINGTNVHPVTGAGKYANVTLTPGKRHRLRLINTSVENHFQVSLVNHSMTVIAADLVPVNAY
TTNTLFL
AVGQRYDVTIDASKIGNYWFNVTLPHPAPAAIFHYAGASGLPTVPGPPVDNVDLVPVVSRTAPGFNPSPNNSL
PVHLSIN
VDWNKPVLQYVIEGNPPSENIVSVASWSYWLIENDVLPHPFHLHGHDFLILGRSPASPATRRDVAMLPAKGWL
LIAFKTD
NPGAWLMHCHIAWHVSGGLSVDFLEG
>lcc1-63
MMVNTIQADWGDYIEITVINKLKSNVTECPIPPGGSKVYRWRATQYGTSWYHSHFSAQYGNGIVGPIVISANY
DVDLGPF
PLTDYYYDNNVLFNGFAKHPTTGAGQYATVSLTKGKKHRLRLINTSVENHFQLSLVNHSMTIISADLVPVQPY
KVDSLFL
GVGQRYDVIIDANQVGNYWFNVTFGNHYPAAIFRYQGAPALPTNQGAPVDLNDLKPVLQRSLTNIALNTGNTI
PITLAIN
INWNKPVLEYVLTGNSQSDNIVQVEGWKYWLIENPDLPHPIHLHGHDFLILGRSPVTAITRRDTAMLPAKGWL
LIAFRTD
NPGSWLMHCHIAWHVSGGLSNQFLER
>lcc1-64
MLVNTIRADWGDWIEVKVINNLRTNVTECPIPPGGQFTYRFRATQYGSSWYHSHFSAQYGNGVVGTIQISLPY
DIDLGVF
PITDYYYADNVLFNGTNVHPTTGAGNYAEVKLTPGKRHRLRLINTSTENHFQLSLVGHQMTIIAADFVPVNAM
TVQSVFL
GVGQRYDVTIDASAPGNYWFNATFGNPAPAAIFRYTNSPALPTNPGPPVDILNLTPVVQRTVVQFVKRPANTL
DVELAIN
VDWSKPVAQYVIEGNPTQDNIVHVNEWTYWLIENDPLPHPFHLHGHDFVVLGRSPATPAIRRDVAMLPAKGWL
LIAFKTN
NPGAWLMHCHIAWHVSHGLSVDFLER
>lcc1-65
MMVNTLQANWGDYLEITVINRLKSNVTECPIPPGGSKVYRFRATQYGTSWYHSHFSAQYGNGIVGSIVISANY
DVDLGPF
SLMDYYYEDNVLFNGFAKHPTTGAGQYATVSLTKGKKHRLRLINTSVENHFQLSLVNHSMTIIAADLVPVQPY
KVDSLLL
GVGQRYDVIIDANQIGNYWFNVTFGNPAPAAIFKYQGAPGLPTNKGAPPDLVDLKPVLQRSLTNIALNTGNTI
PITLAIN
VDWNKPVLEYVMTGLSESDNIVKVDGWKYWLIENPDLPHPIHLHGHDFLILGRSPVAAITRRDVAMLPAKGWL
LIAFRTD
NPGAWLMHCHIAWHVSGGLSNQFLER
>lcc1-66
MMVNTIQADWGDYIEITVINKLKSNVTECPIPPGGSKVYRWRATQYGTSWYHSHFSAQYGNGIVGPIVISANY
DVDLGPF
PLTDYYYDNNVLFNGFAKHPTTGAGQYATVSLTKGKKHRLRLINTSVENHFQLLLVNHSMTIISADLVPVQPY
KVDSLFL
GVGQRYDVIIDANQVGNYWFNVTFGNHYPAAIFRYQGAPALPTNQGAPVDLNDLKPVLQRSLTNIALNTGNTI
PITLAIN
INWNKPVLEYVLTGNSQSDNIVQVEGWKYWLIENPDLPHPIHLHGHDFLILGRSPVTAITRRDTAMLPAKGWL
LIAFRTD
NPGSWLMHCHIAWHVSGGLSNQFLER
>lcc1-67
MMVNTIQADWGDYIEITVINKLKSNVTECPIPPGGSKVYRWRATQYGTSWYHSHFSAQYGNGIVGPIVISANY
DVDLGPF
PLTDYYYDNNVLFNGFAKHPTTGAGQYATVSLTKGKKHRLRLINTSVENHFQLLLVNHSMTIISADLVPVQPY
KVDSLFL
GVGQRYDVIIDANQVGNYWFNVTFGNHYPAAIFRYQGAPALPTNQGAPVDLNDLKPVLQRSLTNIALNTGNTI
PITLAIN
INWNKPVLEYVLTGNSQSDNIVQVEGWKYWLIENPDLPHPIHLHGHDFLILGRSPVTAITRRDTAMLPAKGWL
LIAFRTD
NPGSWLMHCHIAWHVSGGLSNQFLER
>lcc1-68
MLVNTIVADWGDWIEVTVNNKLDTNVTECPIPPNHSFKYRFRAEQYGSSWYHSHFSAQYGNGVVGTILISLPY
EVDLGVF

PITDYYYKDNVLFNGTNIHPTSGGGAYSITKLQPGKRHRLRIINTSTENHFQLSLVGHQMTIIAADFVPVNAM
TVDSVFL
AVGQRYDVTIDASQPGNYWFNATFGNPTPAAIFRYEGAPTLPTNPGRPADILNLTPVVQRTVITFTPRPANTL
HVSLAIN
VNWEKPVAQYVMEGNPTSDNIVHVNEWTYWLVENDPLPHPFHLHGHDFVVLGRSPATPAVRRDVTMLPAKGWI
VIAFKTN
NPGAWLMHCHIAWHVSHGLSVDFLER
>lcc1-69
MLVNTIYAQWGDTITVRVINNLETNITECPLPPGSERTYTYIARQYGTSWYHSHFSAQYGNGVVGAIHISLPY
DIDLGPF
ILSDYYHKDNVLFNGTTVDPDTSAGEYAKITLTPGKRHRLRLINTSVENHFQVSIVDHSMTIISSDFVPVNSF
TTDSIFI
GVGQRYDVTIDASQVANYWFNVTYGNPYPAAIVQYDGAPGNPTDEGAPIDTMDISPVVSRTVTAFTASSGNTM
DVHLSEK
VDWDKPVAQYVAENEPSDLNTWTVDEWVYWLIENPNLPHPIHLHGHDFVVLGRSPTSPIVRRDVTMLPAAGWL
VLAFKTD
NPGAWLMHCHIAWHVAGGLSVTFLER
>lcc1-70
MMVNTIQADWGDYIEITVINKLKSNVTECPIPPGGSKVYRWRATQYGTSWYHSHFSAQYGNGIVGPIVISANY
DVDLGPF
PLTDYYYDNNVLFNGFAKHPTTGAGQYATVSLTKGKKHRLRLINTSVENHFQLSLVNHSMTIISADLVPVQPY
KVDSLLL
GIGQRYDVIIDANQVGNYWFNVTFGNKYPAAIFRYQGAPALPTNKGAPPDLNDLKPVLQRSLTNIALNTGNTI
PITLAIN
INWNKPVLEYVMTGNSQSDNIVQVEGWKYWLIENPDLPHPIHLHGHDFLILGRSPVTAITRRDTAMLPAKGWL
LIAFRTD
NPGSWLMHCHIAWHVSGGLSNQFLER
>lcc1-71
MMVNTLQADWGDYFEITVINRLKSNVTECPIPPGGSKVYRFQATQYGTSWYHSHFSAQYGNGIVGAIVISANY
DVDLGAF
PLMDYYYEDNVLFNGFAKHPTTGAGQYATVSLTKGKKHRLRLINTSVENHFQLSLVNHSMTIIAADLVPVQPY
KVDSLLL
GVGQRYDVIIDANQVGNYWFNVTFGNPKPAAIFKYQGAPGLPTNQGVPPDLVDLKPVLQRSLTDIALNPGNTI
PITLAMN
VDWNKPVLEYVLTGNSQSDNIVQIDGWKYWLIENPDLPHPIHLHGHDFLVLGRSPVAAITRRDVAMLPAKGWL
LIAFRTD
NPGAWLMHCHIAWHVSGGLANQFLER
>lcc1-72
MMVNTLQADWGDYLEITVINKLKSNVTECPIPPGGSKVYRWRATQYGTSWYHSHFSAQYGNGVVGPIVISANY
DVDLGPF
PLTDYYYDNNVLFNGFAKHPTTGAGQYATVSLTKGKKHRLRLINTSVENHFQLSLVNHSMTIISADLVPVQPY
KVDSLLL
GVGQRYDVIIDANQVGNYWFNVTFGNKYPAAIFRYQGAPALPTNKGAPPDLNDLKPVLQRSLTNIALNTGNTI
PITLAIN
INWNKPVLEYVLTGNSQSDNIVQVDGWKYWLIENPDLPHPIHLHGHDFLILGRSPVTAITRRDTAMLPAKGWL
LIAFRTD
NPGSWLMHCHIAWHVSGGLSNQFLER
>lcc1-73
MMVNTLQADWGDYFEITVINRLQSNVTECPIPPGGSKVYRFRATQYGTSWYHSHFSAQYGNGIVGAVVISANY
DVDLGPF
PLMDYYYEDNVLFNGFAKHPTTGAGQYATVSLTKGKKHRLRLINTSVENHFQLSLVNHSMTIIAADLVPVQPY
KVDSLLL
GVGQRYDVIIDANQVGNYWFNVTFGNPKPAAIFKYHGAPGLPTNQGNPPDLVDLKPVLQRSLTDIALNPGNTI
PITLAIN
VDWNKPVLEYVLTGNSQSDNIVQIDGWKYWLIENPDLPHPIHLHGHDFLVLGRSPVAAITRRDVAMLPAKGWL
LIAFRTD
NPGAWLMHCHIAWHLSGGLSNQFLER
>lcc1-74
MMVNTLQANWGDYLEITVINRLKANVTECPIPPGGSKVYRFRATQYGTSWYHSHFSAQYGNGIVGSIVVSANY
DVDLGPF
PLMDYYYEDNVLFNGFAKHPTTGAGQYATVSLTKGKKHRLRLINTSVENHFQLSLVNHSMTIIAADLVPVQPY
KVDSLLL

GVGQRYDVIIDANQIGNYWFNVTFGNPAPAAIFKYQGAPGLPTNKGAPPDLVDLKPVLQRSLTNIALNTGNTI
PITLAID
VNWNKPVLEYVMTGLSESDNIVKVDGWKYWLIENPDLPHPIHLHGHDFLILGRSPVPAITRRDVAMLPAKGWL
LIAFRTD
NPGAWLMHCHIAWHVSGGLSNQFLER
>lcc1-75
MMVNTLEADWGDYLEITVINKLKSNVTECPIPPGGSKVYRWRATQYGTSWYHSHFSAQYGNGIVGPIVISANY
DVDLGPF
PLTDYYYDNNVLFNGFAKHPTTGAGQYATVSLTKGKKHRLRLINTSVENHFQLSLVNHSMTIISADLVPVQPY
KVDSLLL
GIGQRYDVIIDANQVGNYWFNVTFGNKYPAAIFRYQGAPALPTNKGAPPDLNDLKPVLQRSLTNIALNTGNTI
PITLAIN
INWNKPVLEYVMTGNSQSDNIVQVDGWKYWLIENPDLPHPIHLHGHDFLILGRSPVTAITRRDTAMLPAKGWL
LIAFRTD
NPGSWLMHCHIAWHVSGGLSNQFLER
>lcc1-76
MMVNTLEADWGDYLEITVINKLKLNVTECPIPPGGSKVYRWRATQYGTSWYHSHFSAQYGNGVVGPIVISANY
DVDLGPF
PLTDYYYDNNVLFNGFAKHPTTGAGQYATVSLTKGKKHRLRLINTSVENHFQLSLVNHSMTIISADLVPVQPY
KVDSLLL
GIGQRYDVIIDANQVGNYWFNVTFGNKYPAAIFRYQGAPALPTNQGAPPDLNDLKPVLQRSLTNIALNTGNTI
PITLAIN
INWNKPVLEYVLTGNSQSDNIVQVDGWKYWLIENPDLPHPIHLHGHDFLILGRSPVTAITRRDTAMLPAKGWL
LIAFRTD
NPGSWLMHCHIAWHVSGGLSNQFLER
>lcc1-77
MMVNTLQADWGDYLEITVINRLKTNVTECPIPPGGSKVYRFRATQYGTSWYHSHFSAQYGNGIVGSIVISANY
DIDLGPF
PLMDYYYEDNVLFNGFAKHPTTGAGQYATVSLTPGKKHRLRLINTSVENHFQLSLVNHSMTIISADLVPVIPR
KVDSLLL
GVGQRYDVIIDANQYGNYWFNVTFGNPKPAAIFRYQGVPALPTIQGAPPDLNDLKPVVQRSFTNIALNPGNEI
PITLAIN
VDWNKPVLEYVLTGDPQSENIVKIDGWKYWLIENPDLPHPIHLHGHDFLILGRSPANVSTRRDVAMLPAKGWL
LIAFRTD
NPGAWLMHCHIAWHVSGGLSNQFLER
>lcc1-78
RMVNTLQANWGDYLEITVINRLKANVTECPIPPGGSKVYRFRATQYGTSWYHSHFSAQYGNGIVGSIVVSANY
DVDLGPF
PLMDYYYEDNVLFNGFAKHPTTGAGQYATVSLTKGKKHRLRLINTSVENHFQLSLVNHSMTIIAADLVPVQPY
KVDSLLL
GVGQRYDVIIDANQIGNYWFNVTFGNPAPAAIFKYQGAPGLPTNKGAPPDLVDLKPVLQRSLTNIALNTGNTI
PITLAID
VNWNKPVLEYVMTGLSESDNIVKVDGWKYWLIENPDLPHPIHLHGHDFLILGRSPVPAITRRDVAMLPAKGWL
LIAFRTD
NPGAWLMHCHIAWHVSGGLSNQFLER
>lcc1-79
MLINTIVADWGDRLNITVINNLRDNVTECPIPPGGKRLYSFLATQYGTGWYHSHFSAQYANGVVGNMVISLPY
DIDLGVF
PISDYYYEDNVLFNGTNIHPVTGAGKYARVKLTPGKRHRLRLINPSVENHFTVSLVDHQMTVIGTDYVPSNAF
TTDSLFI
GVGQRYDVTIDASKVGNYWFNVTFGNPFPAAIFSYDGAPANPTKAGPPADNLNLTPVVPRSAPSFTPSASNKL
DVHLSIK
VDWQKPIGQYVMDGNPANANIVKVDAWTYWLVENDPLPHPFHLHGHDFLVLGRSPDTPPTRRDVTMLPAKGWV
LIAFKTD
NPGAWLMHCHIAWHVSGGLSVDFLER
>lcc1-80
MLINTIVADWGDTIQITVKNSLQTNATECPIAPGTSKVYTFRALQYGSSWYHSHFSAQYANGVVGTIQISLPY
DVDLGVF
PITDYYYDDNVLFNGTNVHPTTGAGKYANVTLTPGKRHRLRIINTSAENHFQLSLVGHTMTIIGTDFVPVNSY
TVNSLFV
AIGQRYDVTIDASQPGNYWFNVTFGNPTPAAIFHYAGAAGLPTNPGVPPDLFNLTPVVQRTVVSFSPNITDTI
PIHLAIR

VDWNKPVDQYVLEGNPQNENVIQANQWAFWLIENDPLPHPIHLHGHDFVLVGRSPSGALVRRDVTMLPPHGWI
VLAFRTD
NPGSWLMHCHIAWHISGGLGVDFVER
>lcc1-81
MLINVITADWGDRITIVVKNSLRANVTECPIPPGGVKTYSFLALQYGTSWYHSHFTAQYGNGVVGTIQISLPY
DIDLGTF
PISDYYYRDNVLFNGTNVHPVTGAGKYANVTLTPGKRHRLRIINTSVENHIQVSLVGHTMTVIAADFVPVNAE
TVNSLFL
GIGQRYDVTIDASQVSNYWFNVTFGNPTPAAIFHYAGASGNPTNPGAPPDLDNLSPVVTRTVVAFNPTPADTL
PVHLSIV
VDWDKPVDEYVLTGNPRSENLIIENSWGFWLISNDPLPHPIHLHGHDFVVVAKSPVTPLPRRDVTMLPPKGWL
IVAFKTD
NPGAWLMHCHIAWHVSGGLSVDFLER
>lcc1-100
MLINVLTADWGDTLVVTVVNNLKTNVTECPIPPGGSRVYTFRATQYGTTWYHSHFSAQYGNGVWGTIVISLPY
DIDLGPF
PISDYYYDNNVLFNGTNVSPLTGKGKYANVTLTHGKRHRLRIINTSVENHFVLSLANHQFTIIAADLVPVNAM
TVDTLFV
AIGQRYDITIDASQVDNYWFNVTFVNQFPAAIFHYAGASDLPKHPGSVSDLINLTPVVSRTVTTVTANAGNEL
PVTLQMI
INWNKPVDQYVMASNPPSDNVVTVDKWVYWVIENPVIPHPIHLHGHDFVLLGRSDNNAAVRRDVVMLPSLGWI
VIAFRTD
NPGTWLMHCHIAWHVSGGLAVNFLER
>lcc1-82
MLVNTIFADWGDRINITVTNNLRTNVTECPIPPGGSKVYSFIAAQYGTSWYHSHFSAQYGNGVVGSIQISLPY
DIDLGVF
PITDYYYRDNVLINGTNVHPVTGAGKYANVTLTPGKRHRLRLINPSVENHFQVSLVNHSMTVIAADLVPVNAF
TTNTLFL
GVGQRYDVTIDASKIGNYWFNVTLPHPAPAAIFHYAGAPGLPTNPGPPVDNTDLVPVVSRTAPGFNPNPNNSL
PVHLSIN
VDWNKPVLQYVIEGNPPSENIISVPSYSYWLIENDLLPHPFHLHGHDFLILGRSPASPVTRRDVTMLPAKGWL
LIAFRTD
NPGAWLMHCHIAWHVSGGLSVDFLEG
>lcc1-83
MLVNTIYGQWGDSISVRVINNLKTNITECPLAPGSERTYNFIARQYGTSWYHSHFSAQYGNGVVGPIQISLSY
DIDLGPF
LLTDYYHKDNVLFNGTTVDPETGAGEYAKITLTPGKRHRLRLINTSVENHFQVSIVSHSMTIISSDFVPVDSF
TTDSLFL
GVGQRYDVTIDASQVGNYWFNVTFGNSYPAAIVQYDGAPGNPTDEGAPIDTLDIVPVVSRTVTAFTASSDNTM
DVHLSEK
VDWNKPVAQYVANNEPSDLNIWKVDEWVYWLIENPNLPHPIHLHGHDFVVLGRSPANPVVRRDVTMLPAAGWL
LLAFKTD
NPGAWLMHCHIAWHVAGGLSVTFLER
>lcc1-84
MLVNTIHAQWGDTISVRVINNLETNITECPIPPGGERQYTFIARQYGSAWYHSHFSAQYGNGIIGAVQVSTPY
DIDLGPM
ILTDYYHDDNVLFNGHTVNPTTGDGEYEKMTLTPGKRHRLRLINTSVDNHFQVSIVGHDMTVIASDFVPVNAF
TTDSLFI
GVGQRYDVTIEAKEIANYWFNVTFGTPYPAAILHYDGAPSLPTNEGRPKDLINLTPVVERKVTAFTPSASNTM
EVQLAIR
TEWENPVAQYIAANRPETNSIWEVEVWVYWLIENPELPHPIHLHGHDFLVVGRSPAAPAVRRDVAMLPAAGWL
LLAFKTD
NPGAWLMHCHIAWHISGGLGMTFLER
>lcc1-85
MLVNTIYAHWGDTISVKVINNLESNITECPIPPGGERVYTFIARQYGSAWYHSHFSAQYGNGIIGPIQISLPY
DVDLGPM
LLTDYYHEDNVLFNGHAVNPSTGDGEYGKLTLTPGKRHRLRLINTSVDNHFQVSIVNHDMTVIASDFVPVNAF
TTDSLFI
GVGQRYDVTIEANQIGNYWLNVTFGNPYPAAILHYDGAPGLPTKKGRPRDLLDLTPVVERKITEFTPSADNTM
EIQLSIR
TVWQNPVAQYVAANRPETNSIWSVNVWVYWLIENPELPHPIHLHGHDFLVVGRSPAAPAVRRDVAMLPAAGWL
LLAFKTD
NPGAWLMHCHIAWHVSGGLGMTFLER

>lcc1-86
MLVNTIYAHWGDTISVKVINNLESNITECPIPPGGERVYTFIARQYGSAWYHSHFSAQYGNGIIGPIQISLPY
DVDLGPM
LLTDYYHEDNVLFNGHAVNPSTGDGEYGKLTLTPGKRHRLRLINTSVDNHFQVSIVNHDMTVIASDFVPVNAF
TTDSLFI
GVGQRYDVTIEANQIGNYWLNVTFGNPYPAAILHYDGAPGLPTKKGRPRDLLDLTPVVERKVTEFTPSADNTM
EIQLSIR
TVWQNPVAQYVAANRPETNSIWSVNVWVYWLIENPELPHPIHLHGHDFLVVGRSPAAPAVRRDVAMLPAAGWL
LLAFKTD
NPGAWLMHCHIAWHVSGGLGMTFLER
>lcc1-87
MLINTIYAQWGDTISVQVINNLKTNITECPLPPNSERVYTYIAHQYGTSWYHSHFSAQYGNGVSGPIHISLPY
DIDLGPF
VLSDYYHKDNVLFNGTTVDPTTGVGDYAKITLTPGKRHRLRLINTSVENHFQLSIVNHNMTIIASDFVPVDAY
TTDSLFI
GVGQRFDVTIDADQVDNYWFNVTYGNPYPAAIVQYDGAAGNPTDQGTPKDTMDIVPVVSRTVTSFTASTDNTM
DVHLSEK
VDWNKPLSQYVANNEPSDLNIWKVDEWVYWLIENPDLPHPIHLHGHDFVVLGRSPANPIVRRDVTMLPAAGWL
LLAFKTD
NPGAWLMHCHIAWHVAGGLSVTFLER
>lcc1-88
MLVNTIHAQWGDTISVRVINNLETNITECPIPPGGERQYTFIARQYGSAWYHSHFSAQYGNGIIGAVQISTPY
DIDLGPM
LLTDYYHDDNVLFNGHAVNPSTGEGQYEKVTLTPGKRHRLRLINTSVDNNFQVSIVGHDMTVIASDFVPVNAF
TTDSLFI
AVGQRYDVTIEAKEIGNYWFNVTFGTPYPAAILHYDGAPSLPTNEGRPRDLSNLTPVVERKVTAFTPSAENTM
EVQLAIR
TEWENPVAQYIASNRPETNSIWQVDVWVYWLIENPELPHPIHLHGHDFLVVGRSPSAPSVRRDVTMLPAAGWL
LLAFKTD
NPGVWLMHCHIAWHISGGLGMTFLER
>lcc1-89
MMINTIYAQWGDTISVRVINNLKTNITECPLPPNSERVYTYIAHQYGTSWYHSHFSAQYGNGVSGPIHISLPY
DIDLGPF
VLSDYYHKDNVLFNGTTVDPTTGVGDYAKITLTPGKKHRLRLINTSVENHFQLSIVNHNMTIIGSDFVPVEAY
DTDSLFI
GVGQRFDVIIDADQVDNYWFNVTYGNPYPAAIVQYDGAAGNPSDRGTPKDTMDIVPVISRTVTSFTASSDNTL
DFHLSEK
VDWNKPLSQYIANNQPSNLNIWKVDEWVYWLIENPDLPHPIHLHGHDFVVLGRSPANPVVRRDVTMLPAAGWL
LLAFKTD
NPGAWLMHCHIAWHVAGGLSVTFLER
>lcc1-90
MLVNTIVADWGDTIQVTVINNLETNLTECPIPPGGSKVYRFRATQYGTSWYHSHFSAQYDNGIVGTILISLPY
EVDLGVF
PITDYYYADNILFNGTNKHPSTGQGRYAEVTLTPGKRHRLRIINTSADASFTVSLVDHDMTVIASDFVPVQSV
TVNKLFV
AIGQRFDVTIDASKVGNYWFNVTFENPHPAAVFRYQGAPTDPTNPGAVVDKLDLVPVVTRTVTSFQPTIDNTL
NVSLAIN
VDWNRPVLEYVMEGNPQSENIISVNGWAYWLIENVGIAHPLHLHGHDFVVLGRSAGAIGARRDVTMLPAQGWV
VLAFRTD
NPGAWLFHCHIAWHVSAGLAVDFLEN
>lcc1-91
MLINVISADWGDTLSITVINNLKSNVTECPIPPGHSRVYTFLATQYGSSWYHSHFSAQYGNGVVGTIQISLPY
DVDLGVF
PVSDYYYHNNVLFNGTNVHPVTGNGKYANVTLTPGKRHRLRIINISVENHFVFSLANHTMTIIAADLVPVNAM
TVNELFV
GVGQRYDVTIDASQPGNYWFNATFVNLTPAAIFHYAGSAGLPTNPGVATDLTNLTPVVKRTVVSFVADSSNEL
NVTLQEI
ISWEKPVDQYLLNSQPNSDNVVVVDKWVFWVVENPVIAHPMHLHGHDFMVVGRADNNPGVRRDVTMLPPLGWV
AIAYKTD
NPGTWLFHCHIAWHVSGGLAVTFAER
>lcc1-92
MLIDTIYADWGDTVEVTVKNSLECNITECPIAPGSTKVYTFVARQYGTGWYHSHFSAQYGNGVVGAIVISLPY
DIDLGAF

PITDYYYNDNVLFNGTNVHPVTGAGTYANVTLTPGKRHRLRIINTSVENHMQVSLVGHSMTVIATDYVPVNAY
TTDTLSV
AIGQRYDVTIDATNPGNYWFNVTYSNPHPAAVFHYKGAPGLPTDVGKPKDQLNFTPVVQRFVVNFNPVVDNTL
PVTVAIR
VDWGKPIDQAILAGNAGSQNVIPTGRWGYWLITNDPVPHPIHLHGHDMVVVGRSPGSAKLRRDVTMLPPRGWI
VVAFRTD
NPGSWLMHCHIAWHASGGLSVDFVER
>lcc1-93
MLINVISADWGDTLSITVINNLKTNVTECPIPPGHSRVYTFLATQYGSSWYHSHFSAQYGNGVVGTIQISLPY
DIDLGVF
PVSDYYYHNNVLFNGTNVHPVTGNGKYANVTLTPGKRHLLRIINISVENHFVFSLANHTMTIVAADLVPVNSM
TVNELFV
GVGQRYDVTIDASQPGNYWFNATFVNLTPAAVFHYAGSAGLPTNPGVATDLTNLTPVVKRTVVSFVADSSNEL
NVTLQEI
ISWEKPVDQYLLNSQPNSDNVVVVDKWVFWVVENPVIAHPMHLHGHDFMVVGRADNNPAVRRDVTMLPPLGWV
AIAYKTD
NPGTWLFHCHIAWHVSGGLAVTFAER
>lcc1-94
MLVNVIHAQWGDTISVTVTNNLKYNITECPIPPGGSKTYTFIAHQYGTSWYHSHFSAQYGNGIVGAIQISLPY
DIDLGPL
VLSDYYYKDNVLFNGTNINPATTQGQYKTITLTPGKRHRLRIINTSVENNFQVSIVGHSMTVIESDFVPVDSF
TTDSLFV
GIGQRYDVTIDASQTDNYWMNVTFGNPYPAAIIHYNGASSHPTNKGAPADLLNLVPVVPRSITSFVAASDNTL
DVQLTLD
VDWGHPITQYVINKSPSTDNVWLVEAWAYWLIENPTLPHPIHLHGHDFVVLGRSPVSPTIRRDVVMLPPKGWL
LIAFQTT
NPGAWLMHCHIAWHVSAGLGNTFLEQ
>lcc1-95
MLVNTIYAQWGDTISVRVINNLETNITECPIPPGGERVYTYIAHQYGTSWYHSHFSAQYGNGISGPIQISLNY
DIDLGPF
VLSDYYHRQNVLFNGKTVNPDTGVGEYAKIKLTRGKRHRLRLINTSVQHNLVVSIVKHDMTVIGTDFVPVNSF
TTNSLFI
GVGQRYDVTIDASQVDNYWFNVSLGNLFPAAIVQYDGAADIPTKEGAPSDSLDLVPVVSRTVTSFTPSSGNTL
DLTLPMK
VDWNKPLAQYVNKNEASSENILKVDDWVYWLIENLDIPHPIHLHGHDFVVIGRSPSAPAVRRDVTMIPAKGWL
LLAFKTD
NPGAWLMHCHIAWHVAGGLSNTFLER
>lcc1-96
MLVNTLFADWGDFIQITVINKLRTNVTECPIPPGGTKVYKFRATQYGTSWYHSHFSAQYGNGVVGAIQISLPY
DIDLGTF
PITDYYYNNNVLFNGTNVHPTTGNGKYANVTLTPGKRHRLRIINTSVENHFQLSLAGHQFTIIAADLIPVQAQ
TVSSLFL
SVGQRLDVTIDASKPGNYWFNATFGNKGAAAIFHYAGAAGLPKDPGAPADLTNLTPVVTRSLVNFIARPNNTL
PVTVAIN
VNWNKPVIDYVLTDNPREENVVQISQWVYWLIENDPIPHPIHLHGHDFLIVGRSPATPLARRDTAMLPARGWL
LLAFKTD
NPGAWLMHCHIAWHVSAGLSIQFLER
>lcc1-97
MLINNLVADWGDQLEITVINGLEINVTECPIPPGSSRVYKFLAQQYGTSWYHSHFSAQYGNGVVGTIQISLNY
DIDLGAF
PVTDYYYDDNVLFNGTNVHPTSGAGKYASVTLTPGKRHRLRIINTSVENHFVFSLVGHSMTIIAADFVPVQPQ
TVDQLFV
GVGQRYDVTIDASMVGNYWFNATFVNPYPAAIFRYKGASALPTTPGTAADLSNLQPVLQRHITKFQSSKDNTL
PVTLKVA
INWEKPVDQYVMQGSPPDDNVVTVTKWIFWVIENPTIAHPIHLHGHDFLLLGRSDNNPTVRRDVVMLPSLGWI
VIGYKTD
NPGTWLMHCHIAWHVSGGLAVNFLER
>lcc1-98
MLVNTINAQWGDTISVKVINNLKSNVTECPIPPGGERQYSFLATQYGNSWYHSHFSAQLGNGIVGPIQISLPY
DIDLGPI
LLTDYYHDDNVLFNGHAVNPSTGDGKYHTVTITAGKRHRLRLINTSAENNFQVSIVGHNMTIIQSDFVPVNAF
TTDSLFI

GIGQRYDVTIEANQVGNYWLNVTFGNPHPAAILHYDGAPGLPTEDGQPRDLLDLKPVVERQLTAFTPSAANTL
DVQLSIR
TDWQNPIAQYVAANRPETNSIFPVEVWVYWLIENPQLPHPIHLHGHDFLVVGRSPSAPAVRRDVTMLPAAGWL
LLAFKTD
NPGAWLMHCHIAWHIAGGLGLTFLER
>lcc1-99
MLVNLIYAQWGDTISVKVTNNLEFNATECPIPPGGTRTYTYIAHQYGTSWYHSHFSAQYGNGVIGPIQISLPY
DIDLGPL
ILSDYYYQDNVLFNGTNINPAPSEGEYKTITLTPGKRHRLRLINTSVENHFQVSIVGHDMTVIGTDFVPVDSF
TTSSLFV
GIGQRYDVTIDASQTDNYWMNVTFGNPYPAAIIHYDGAAGNPTNLGVPTDLINFTPVVSRSVTSFTATGDNTL
DIESSLV
VDWGHPLAQYAIENSPSSDNVYQIDEWVYWLIQNPALPHPIHLHGHDFVVLGRSPEAPQLRRDVTMLPAKGWL
LLAFKTD
NPGAWLMHCHIAWHASGGLGLSFLEN

Thermoth elomyces thermophilu s ATCC:

>XP_003663741.1:1-616 extracellular laccase, lcc1 [Thermothelomyces thermophilus ATCC 42464]
MKSFISAATLLVGILTPSVAAAPPSTPEQRDLLVPITEREEAAVKARQQSCNTPSNRACWTDGYDIN
TDYEVDSPDTGVV
RPYTLTLTEVDNWTGPDGVVKEKVMLVNRPTIFADWGDTIQVTVINNLETNGTSIHWHGLHQK
GTNLHDGANGITECPIP
PKGGRKVYRFKAQQYGTSWYHSHFSAQYGNGVVGAIQINGPASLPYDTDLGVFPISDYYYSSADE
LVELTKNSGAPFSDN
VLFNGTAKHPETGEGEYANVTLTPGRRHRLRLINTSVENHFQVSLVNHTMTIIAADMVPVNAMT
VDSLFLGVGQRYDVVI
EASRTPGNYWFNVTFGGGLLCGGSRNPYPAAIFHYAGAPGGPPTDEGKAPVDHNCLDLPNLKP
VVARDVPLSGFAKRPDN
TLDVTLDTTGTPLFVWKVNGSAINIDWGRPVVDYVLTQNTSFPPGYNIVEVNGADQWSYWLIEN
DPGAPFTLPHPMHLHG
HDFYVLGRSPDESPASNERHVFDPARDAGLLSGANPVRRDVTMLPAFGWVVLAFRADNPGAWL
FHCHIAWHVSGGLGVVY
LERADDLRGAVSDADADDLDRLCADWRHYWPTNPYPKSDSGLKHRWVEEGEWLVKA
>6F5K_A:1-559 Chain A, Extracellular laccase, lcc1 [Thermothelomyces thermophilus]
QQSCNTPSNRACWTDGYDINTDYEVDSPDTGVVRPYTLTLTEVDNWTGPDGVVKEKVMLVNNS
IIGPTIFADWGDTIQVT
VINNLETNGTSIHWHGLXQKGTNLHDGANGITECPIPPKGGRKVYRFKAQQYGTSWYHSHFSA
QYGNGVVGAIQINGPAS
LPYDTDLGVFPISDYYYSSADELVELTKNSGAPFSDNVLFNGTAKHPETGEGEYANVTLTPGRRHR
LRLINTSVENHFQV
SLVNHTMTIIAADMVPVNAMTVDSLFLGVGQRYDVVIEASRTPGNYWFNVTFGGGLLCGGSRNP
YPAAIFHYAGAPGGPP
TDEGKAPVDHNCLDLPNLKPVVARDVPLSGFAKRPDNTLDVTLDTTGTPLFVWKVNGSAINIDW
GRPVVDYVLTQNTSFP
PGYNIVEVNGADQWSYWLIENDPGAPFTLPHPMHLHGHDFYVLGRSPDESPASNERHVFDPAR
DAGLLSGANPVRRDVTM
LPAFGWVVLAFRADNPGAWLFHCHIAWHVSGGLGVVYLERADDLRGAVSDADADDLDRLCAD
WRRYWPTNPYPKSDSGL
>KAK4035452.1:1-619 laccase [Parachaetomium inaequale]
MRPFFGAAALMVGILAPSAVAAPPSTPAQRDLLVPVAERQEADLLPRQSSCHTPSNRACWSTGYN
INTDYEVNSPNTGVV
RPYTLTLTEVNNWTGPDGVVKNKVMLINSMSWPTIFANWGDTIQVTVINNLETNGTSIHWHGM
HQKDTNLHDGANGVTEC
PIPPKGGRRVYRFLAQQYGTSWYHSHFSAQYGNGVVGTIQINGPASLPYDIDLGVFPLMDYYYRS
ADELVHFTMNNGAPF
SDNVLFNGTAKHPTTGAGQYANVTLTPGKRHRLRIINTSTENHFQVSLVNHTMTVIASDMVPVN
AFTVDSLFLAVGQRYD
VTIDASRAPGNYWFNVTFGGQAFCGGSLNPSPAAIFHYAGAPGGLPTDRGVAPVDHQCLDLPNL
TPVVTRNVPVSGFVKK
PSNTLPVKLDLTGTPLFVWKVNGSAVNVDWGKPVLDYVMTQNTSYPASDNIVQVDGVDQWTY
WLVENDPDGAFSLPHPMH
LHGHDFLVLGRSPAVPPGSQQRFVFDPAVDLPRLRGTNPVRRDVTMLPAKGWLLLAFKTDNPGA
WLFHCHIAWHVSGGLS
VDFLERPNELRQRITSGDRDDFNRVCREWREYWPTNPFPRVDSGLRHRFVEEGEWMVTA
>KAH6845722.1:1-616 laccase [Chaetomium sp. MPI-CAGE-AT-0009]
MKFLFGAMALTVGIFVPSVAAAPPSTAAQRDLLVPIAERQEAGVLPRQASCHTPSNRACWTTGY
NINTDYEVNSPDTGVV

RPYIFTLTEEENWTGPDGVVKNKVMLINRPTIFADWGDTIEVTVINNLDTNGTSIHWHGMHQKD
TNLHDGANGVTECPIP
PNGGQRVYRFKAQQYGTSWYHSHFSAQYGNGVVGTIQINGPASLPYDIDLGVFPLMDYYYRSAD
ELVHFTMNNGAPFSDN
VLFNGTAKHPITGVGEWANVTLTPGKRHRLRIINTSTENHFQVSLVNHSMTIIASDMVPVNAMTV
DSLFLAVGQRYDVTI
DANRTPGNYWFNVTFGGQAFCGGSLHPSPAAIFHYAGAPGGLPTNQGVPPTDHQCLDLPNLTP
VVTRNVPVSGFVKRPGN
TLPVTLDLSGTPLFVWKVNGSAINVDWGKPILDYVMTQNTSYPTSDNIVQVDGVNQWTYWLVE
NDPDGPFSLPHPMHLHG
HDFLVLGRSPDVSPASQQRFVFDPAVDLPRLRGTNPVRRDVTMLPPRGWLLVAFRTDNPGAWLF
HCHIAWHVSGGLSVDF
LERPNDLRQRITPADRDDFNRVCEEWRAYWPTNPFPKIDSGLRHRFVEESEWMVRA
>KAK3299560.1:1-620 laccase [Chaetomium fimeti]
MKFLFGVVALTVGILVPSVAAAPPSTAAQRDLLVPIAERQEAGVLPRQASCHTPSNRACWTTGYN
INTDYEVDSPDTGVV
RPYTFTLTEKENWPGPDGVVKNKVMLINDNIMGPTVFADWGDTIEVTVINNLDTNGTSIHWHG
MHQKDTNLHDGANGVTE
CPIPPNGGQRVYRFKAQQYGTSWYHSHFSAQYGNGVVGTIQINGPASLPYEIDLGVFPLMDYYYR
SADELVHFTMDNGAP
FSDNVLFNGTAKHPITGVGEWANVTLTPGKRHRLRIINTSTDNHFQVSLVNHSMTIIASDMVPVN
AMTVDSLFLAVGQRY
DVTIDANSTPGNYWFNVTFGGQASCGGSLHPTPAAIFHYAGAPGGLPTNQGSPPTDHQCLDLPN
LTPVVTRNVPVSGFVK
RPGNTLPVTIDLTGTPLFVWKVNGSAINVDWGKPILDYVMTQNTSYPTSDNIVQVDGVNQWTY
WLVENDPDGPFSLPHPM
HLHGHDFLVLGRSPDVPPASQQRFVFDPAVDLPRLRGTNPVRRDVTMLPAGGWLLLAFRTDNPG
AWLFHCHIAWHVSGGL
SVDFLERPDDLRQRITPADRDDFNRVCEEWREYWPTNPFPKIDSGLRHRFVEESEWMVKA
>KAH6637198.1:1-619 laccase [Chaetomium globosum]
MKFFFGTLALTVGLLVPGGVAAPPSAASQRDLLVPIAERQEAAVLPRQTSCHTPSNRACWTTGYN
INTDYEVNSPDTGVV
RPYTFTLTEEENWTGPDGVVKNKVMLINNKIMGPTIFADWGDTIQVTVINNLDTNGTSIHWHG
MHQKDTNLHDGANGVTE
CPIPPGGQRVYRFKAQQYGTSWYHSHFSAQYGNGVVGTIQINGPASLPYDIDLGVFPLMDYYYAS
ADELVHFTMNNGAPF
SDNVLFNGTAKHPTTGAGQWANVTLTPGKRHRLRIINTSTENHFQVSLVNHSMTVIASDMVPVN
AMTVDSLFLAVGQRYD
VTIDANRTPGNYWFNVTFGGQAFCGGSLNPTPAAIFHYAGAPGGLPTNKGVPPTDHQCLDLPN
LTPVVTRNVPVSGFVKR
PGNTLPVNIDLSGTPLFVWKVNGSSINVDWGKPVADYVMTQNTSYPTSDNIVQVDGANQWTYW
LVENDPDGPFSLPHPMH
LHGHDFLVLGRSPDVSPASQQRFVFDPAVDLPRLRGTNPVRRDVTMLPPRGWLLLAFRADNPGA
WLFHCHIAWHVSGGLS
VTFLERPNELRQRITPADRADFNRVCGEWREYWPTNPFPKVDSGLRHRMVEESEWMVKA
>KAK4120739.1:1-615 multicopper oxidase [Parathielavia appendiculata]
MKFFLGVVALMLGAIAPSVVAAPPATPVQRDLLVPLEERQDDTARRQTGCHTPSNRACWAPGFN
INTDYEVNSPNTGVVR
PYTLTLTEIDNWTGPDGVVKNKVMLVNGPTIFANWGDTIQVTVINNLITNGTSIHWHGMHQKN
TNLHDGANGVTECPIPP
KGGRKVYRFRAQQYGTSWYHSHFSAQYGNGVVGTIQINGPASLPYDIDLGVFPLMDYYYKSADE
LVHFTMNNGPPFSDNV

LFNGTAKHPTTGAGQYANVTLTPGKRHRLRIINTSTENHFQVSLVNHTMTVIAADMVPVNAMT
VDSLFLGVGQRYDVTID
ASRTPGNYWFNVTFGGQAFCGGSLNPHPAAIFHYAGAPGGLPTDRGTPPVDHQCLDLPNLTPVV
TRNVPVNGFVKKPSNT
LPVNLDLTGTPLFVWKVNGSAINVNWNKPVLEYVMTGNTSYPASDNIVQVDGVDQWTYWLVE
NDPDGAFSLPHPMHLHGH
DFLVLGRSPDVPPGSQQRFVFDPTVDLPRLRGANPVRRDVAMLPARGWLLLAFKTDNPGAWLFH
CHIAWHVSGGLSVDFL
ERPNDLRQRISPADRNDFNRVCNEWRAYWPTNPYPKIDSGLRHRFVEESEWMVRA
>KAH6616152.1:1-620 laccase [Chaetomium sp. MPI-SDFR-AT-0129]
MKSIFIRAALIVGVLASRAVAAPPSTPAQRDLLVPIVDRDETGLAARQSSCNTPSNRACWSNGFDI
NTDYEVKSPNTGVV
RPYTFTLTEVDNWTGPDGVVKEKVMLINNKIIGPTIFADWGDTIQVTVINNLETNGTSIHWHGLH
QKGTNLHDGANGLTE
CPIPPQGGRKVYRFKAQQYGTSWYHSHFSAQYGNGVVGTIHINGPASLPYDIDLGVFPLMDYYYR
TADELVAFTQNNGAP
FSDNVLFNGSAKHPTTGAGQYANVTLTPGKRHRLRIINTSTENHFQVSLVDHKFTIIASDLVPVQA
QTVDSLFLAVGQRY
DVTIDGNAAPGNYWFNVTFGGQAACGGSLNPNPAAIFHYAGSSGGLPTKQGVVPVDHQCLDLP
NLTPVVQRTVPVSGFTK
KADNTLTVNIDLTGTPLFVWKVNGSAINVDWNKPVLEYVMTQNNSIPRSENIVQIDAADQWTY
WLIENDPNGPFSLPHPM
HLHGHDFLVLGRSPDVPPGSNQRFTFDPTVDLPRLRGSNPVRRDVTILPAGGWLLLAFKSDNPGA
WLFHCHIAWHVSGGL
SVDFLERPNDLRQQITPGDRDDFNRVCDEWRAYWPTNPFPKVDSGLRHRFVEESEWLVKA
>XP_001228806.1:1-619 laccase [Chaetomium globosum CBS 148.51]
MKFFFGALALTVGLLVPGGAAAPPSAASQRDLLVPIEERQEAAVLPRQTSCHTPSNRACWTTGYN
INTDYEVNSPDTGVV
RPYTFTLTEEENWTGPDGVVKNKVMLINSMSCRPTIFADWGDTIQVTVINNLDTNGTSIHWHGM
HQKDTNLHDGANGVTE
CPIPPGGRRVYRFKAQQYGTSWYHSHFSAQYGNGVVGTIQINGPASLPYDIDLGVFPLMDYYYAS
ADELVHFTMNNGAPF
SDNVLFNGTAKHPTTGAGQWANVTLTPGKRHRLRIINTSTENHFQVSLVNHTMTVIASDMVPVN
AMTVDSLFLAVGQRYD
VTIDANRTPGNYWFNVTFGGQAFCGGSLNPTPAAIFHYAGAPGGLPTNRGVPPTDHQCLDLPNL
TPVVTRNVPVSGFVKR
PGNTLPVNIDLSGTPLFVWKVNGSAINVDWGKPVADYVMTQNTSYPTSDNIVQVDGANQWTY
WLVENDPDGPFSLPHPMH
LHGHDFLVLGRSPDVSPASQQRFVFDPTVDLPRLRGTNPVRRDVTMLPPRGWLLLAFRADNPGA
WLFHCHIAWHVSGGLS
VTFLERPNELRQRITPADRADFNRVCEEWREYWPTNPFPKVDSGLRHRMVEESEWMVKA
>KAK4145141.1:1-620 laccase [Dichotomopilus funicola]
MKSILARVALVVGILASRAVAAPPSTPAQRDLLVPIVDRDESGLAPRQSSCNTPSNRACWSNGFDI
NTDYEVKSPDTGVV
RPYTFTLTEVDNWTGPDGVVKEKVMLINNKIIGPTIFADWGDTIQVTVINNLETNGTSIHWHGLH
QKGTNLHDGANGLTE
CPIPPQGGRKVYRFKAQQYGSSWYHSHFSAQYGNGVVGTIHINGPASLPYDIDLGVFPLMDYYYR
TADELVAFTQNNGAP
FSDNVLFNGSAKHPTTGAGQYANVTLTPGKRHRLRIINTSTENHFQVSLVDHKFTIIASDLVPVQA
QTVDSLFLAVGQRY
DVTIDGNAAPGNYWFNVTFGGQAACGGSLNPNPAAIFHYAGSSGGLPTKQGVVPVDHQCLDLP
NLTPVVQRTVPVSGFTK

KADNTLTVNIDLTGTPLFVWKVNGSAINVDWNKPVLEYVMTQNNSIPRSENIVQIDTADQWTY
WLIENDPNGPFSLPHPM
HLHGHDFLVLGRSPDVPPGSNQRFTFDPTVDLPRLRGSNPVRRDVTILPAGGWLLLAFKSDNPGA
WLFHCHIAWHVSGGL
SVDFLERPNDLRQQITPGDRDDFNRVCDEWRAYWPTNPFPKVDSGLRHRFVEESEWLVKA
>KAK4249145.1:1-615 extracellular laccase [Corynascus novoguineensis]
MKPFIGAAALMMGVLAPSVAAAPPSTPAQRDLLVPIMERDEATIVPRQESCNTPSNRACWSEGFD
INTDYEVETPDTGVV
RHYNLTLTEVDDWLGPDGVVKKKVMLVNGPTIYGDWGDTIEVTVINNLRTNGTSIHWHGLHQK
DNNLHDGANGITECPIP
PKGGRKVYRFRARQYGTSWYHSHFSAQYGNGVVGSIQINGPASLPYDIDLGVFPITDYYYLAADE
LVHMTQVGPPPFSDN
VLFNGTAKHHETGDGEYATVTLTPGKRHRLRLINPSVENHFQVSLVNHTMTIIASDMVPVQAQT
VDSLFLGVGQRYDVVI
DASSTPGNYWFNVTFGGANACGGSRHPNPAAIFHYAGAPDALPTDEGTPPVDHQCLDLDNLTP
VLERNVPVNDFVQNPGN
TLDISLDLTPIFVWKINGSAINIDWGKPAVDYVLTGNTSFPREYNIVQVDGVDQWTYWLIENDPD
GIVSLPHPMHLHGHD
FYVLGRSPVTSPGDNLKFRFDPAVDRGRLSGDNPTRRDVAMVPARGWLLIAFKTDNPGAWLFHC
HIAWHVSGGLSVTFLE
RPNDLRDAVEGTADEQELNRVCEEWREYWPTNPYPKIDSGLKHRWVEESEWFVKA
>KAK4148794.1:1-620 laccase [Chaetomidium leptoderma]
MKSFLGVVALMVGILAPSVIAAPPSTPAQRDLLLPIEELQGPAMTPRQTSCHSPSNRACWSTGFNI
NTDYEVSTPNTGNV
RTYTLTLTEANNWTGPDGVVKNKVMLVNSMLCRPTIFADWGDTIQITVINKLETNGTSIHWHGM
HQRDTNYHDGANGVTE
CPIPPNGGQRVYRFRAQQYGSSWYHSHFSAQYGNGVVGTILINGPASLPYEVDLGVFPLMDYYYR
SADELVHFTMSNGAP
FSDNVLFNGSAKHPTTGVGSYANVTLTPGKRHRLRIINTSTENHFQVSLVNHTMTVIASDMVPVN
AMTVDSLFLAVGQRY
DVTIDASRTPGNYWFNVTFGGQAFCGGSLNPFPAAIFHYAGALGGLPTNKGVTPTDHQCLDRLD
LTPVLTRTVPASAFVK
KPSNTLPVHLEIGGTPLFVWKVNGSSMNIDWGKPVLDYVMTQNTSYQTGDNVVQVDGVDQWT
YWLVENDPDGAFGLPHPM
HLHGHDFFVLGRSPDVSPASQQRFVFDPTTDLPRLRGSNPVRRDVTMLPPRGWLLLAFKTTNPG
AWLFHCHIAWHVSGGL
SVQFLERPNDLRQRLTAADKADFNRVCNAWRAYWPTNPFPKIDSGLKHRFVEESEWMVKA
>KAG7284776.1:1-612 sphingosine N-acyltransferase lac1 [Staphylotrichum longicolle]
MKSFLGAVALMVGILAPSVTAAPPTTPVQRDLLAPLEERQSSCHTPSNRACWSTGFSINTDYETNV
PTTGATRPYTLTLT
EADNWTGPDGVVKNKVMLVNGKILGPTIRANWGDWIQVTVINNLRTNGTSIHWHGMHQKDT
NLHDGANGVTECPIPPNGG
QRVYRFRAAQYGTSWYHSHFSAQYGNGIVGTIQIEGPASLPYDIDLGVFPLSDYYYKSADELVHFT
MNNGPPFSDNVLFN
GTGKHPVTGAGQYANVTLTPGKRHRLRIINTSTENHFQVSLVDHSMTVIASDMVPVNAMTVDSL
FLAVGQRYDVTIDASK
TPGNYWFNVTFGGSAFCGGSLNPNPAAIFHYAGAPGGLPTKQGTPPVDHQCLDNMNLSPVVTR
SAPVSGFVKKPGNTLPV
TLDLTGTPLFVWKVNASAINVDWQKPVLDYVMTQNTNYPPGDNLVQIDSVDQWTYWLVENDP
DGPFSLPHPMHLHGHDFL
VLGRSPDVPPASQQRFVFNPTVDLPRLRGTNPVRRDVTMLPAKGWLLLAFKSDNPGAWLFHCHI
AWHVSGGLSVDFLERP

NELRQRITTADRNDFNRVCNEWRSYWPTNPFPKIDSGLKHRFVEESEWLVKA
>KAK4237176.1:1-624 laccase [Achaetomium macrosporum]
MRSFFGAVALVMGILAPSVIAAPPITPAQRDLLVPLEQRQGSALRPRQTSCNTPSNRACWSDGFDI
NTDYETSTPDTGFT
REYTLTLTEADNWQGPDGVVKEKVMLVNGMSARAWGWPTIFADWGDTIEVTVINNLETNGTSI
HWHGIRQLNTVHHDGVN
GITECPIPPNGGTRVYRFRAQQYGTTWYHSHFSAQYGNGVVGTIQINGPASLPYDIDLGVFPITDY
YYRTADELVEFTKN
NGAPFADNVLFNGTNVHPTTGAGSYANVTLTPGRRHRLRLINTSTENHFQLSLVNHSMTIIAADL
VPVEALTVDSLFIGV
GQRYDVVIDASQTPGNYWFNATFGGGNFCGGSNNPTPAAIFHYEGAPGGLPTDTGVVPADHQC
LDNISLTPVVERNAPVN
NFEKTPGNTLDVHLDTTGTPLFVWKVNGSAINVDWGKPVLDYVITGNTSYPPSNNLVQVDEAD
AWTYWLIENDPDGGFAL
PHPMHLHGHDFLVLGRSPDVPPATQQRFVFDPAVDLPRLKGNNPVRRDVAMLPAKGWLLLAFR
TDNPGAWLFHCHIAWHV
SGGLSVTFLERAEDMRSRFSDADKDDFNRVCGEWNAYAPSAPPKTDSGLKIRHPWVEKSEWMVR
>Q70KY3.1:1-623 RecName: Full=Laccase-1; AltName: Full=Benzenediol:oxygen oxidoreductase 1; AltName: Full=Diphenol oxidase 1; AltName: Full=Ligninolytic phenoloxidase; AltName: Full=Urishiol oxidase 1; Flags: Precursor [Melanocarpus albomyces]
MKTFTSALALVVGMLAPGAVVAAPPSTPAQRDLVELREARQEGGKDLRPREPTCNTPSNRACWS
DGFDINTDYEVSTPDT
GVTQSYVFNLTEVDNWMGPDGVVKEKVMLINGNIMGPNIVANWGDTVEVTVINNLVTNGTSIH
WHGIHQKDTNLHDGANG
VTECPIPPKGGQRTYRWRARQYGTSWYHSHFSAQYGNGVVGTIQINGPASLPYDIDLGVFPITDY
YYRAADDLVHFTQNN
APPFSDNVLINGTAVNPNTGEGQYANVTLTPGKRHRLRILNTSTENHFQVSLVNHTMTVIAADM
VPVNAMTVDSLFLAVG
QRYDVVIDASRAPDNYWFNVTFGGQAACGGSLNPHPAAIFHYAGAPGGLPTDEGTPPVDHQCL
DTLDVRPVVPRSVPVNS
FVKRPDNTLPVALDLTGTPLFVWKVNGSDINVDWGKPIIDYILTGNTSYPVSDNIVQVDAVDQWT
YWLIENDPEGPFSLP
HPMHLHGHDFLVLGRSPDVPAASQQRFVFDPAVDLARLNGDNPPRRDTTMLPAGGWLLLAFRT
DNPGAWLFHCHIAWHVS
GGLSVDFLERPADLRQRISQEDEDDFNRVCDEWRAYWPTNPYPKIDSGLKRRRWVEESEWLVR
>KAK3901697.1:1-613 laccase [Staphylotrichum longicolle]
MKSFLGVMALVAGFLAPRVVAAPPTTPLQRDLLNPIELRQTSCNTPSNRACWSTGFNINTDYETST
PTTGVTRPYTLTLT
EVDNWTGPDGVVKNKVMLVNGMLECRPTIFADWGDWIEITVINNLRTNGTSIHWHGIRQQNTI
FHDGANGVTECPIPPQG
GTRKYRFRAQQYGSSWYHSHFSAQYGNGIVGTIQINGPASLPYDIDLGVFPLTDYYYRSADDLVLF
TQSNGPPFSDNVLI
NGTLKHPTSGAGSYATVNLTPGKRHRLRIINTSTENHFQVNLQNHTMTVISADFVPVNAMTVDS
LFLGVGQRYDVTIDAS
RAVGNYWFNITFGGQNFCGGSLNPNPAAVFHYNGAPNALPTNQGVAPIDHQCLDLPNLTPVVT
RNVPVSGFVKKPQNTLP
VHLEIGGTPLFVWKVNGSAVNVDWNKPVLEYVMTQNTSYPPGDNIVQVDAVDQWVYWLVEN
DPDGAFSLPHPMHLHGHDF
VVLGRSPFATPASQQRFVFNPATDLPALNGANPVRRDVTMLPPKGWLLLAFKTDNPGAWLFHC
HIAWHVSGGLSVDFLER
PTDLRNRLTTADKNEFNRLCNDWRTYWPTNPFPKGDSGLKHRFVEESEWLVKA
>KAK3306164.1:1-623 laccase-1 [Chaetomium strumarium]

MKPFLGAVALVMGMLAPSVIAAPPATPAQRDLLVPLEQEQESALRARQATSASCNTPSNRACWS
NGFNINTDYETSTPNT
GVTRKYTLTLTETDNWQGPDGVVKEKVMLVNGTILGPTILADWGDKIEVTVINNLKTNGTSIHW
HGIRQQNSIYHDGVNG
VTECAIPPKGGKRVYKFRAQQYGTSWYHSHFSAQYGNGVVGAIQINGPASLNYDIDLGVFPITDY
YYRTADELVEFTKNN
GAPFADNVLFNGTNVHPTTGAGRYANVTLTPGKRHRLRIINTSTENHFQLSLVNHSMTIIAADLV
PVQAFTVDSVFLAVG
QRLDVTIDASKTPGNYWFNATFGGGNFCGGSSNPSPAAIFHYAGAPGGLPTDRGVTPADHQCLD
NVNLTPVVKRTAPVNN
FQKTPGNTLDVHLDTTGTPLFVWKVNGSTINVDWGKPVLDYVITGNTSYPSSNNLVQVDEKDA
WTYWLIENDPDGGFSLP
HPMHLHGHDFLVLGRSPDVPPATQQRFVFDPAVDLPRLKGANPVRRDVAMLPAKGWLLLAFRT
DNPGAWLFHCHIAWHVS
GGLSVTFLERAADLRSRIPDADKQDFNRVCAEWNAYAPSAPPKTDSGLKARRPWVEKSEWIVR
>KAK4101287.1:1-606 multicopper oxidase [Parathielavia hyrcaniae]
MKVVVGLVALLLSILAPRAAAMPSSTPGQKFGDFIVRQNSCHNASNRACWTPGWNINTDYEVRT
PNTGVVRPYTLTLTEV
DNWTGPDGVVKNKVMLVNGPTIVANWGDTIRVTVINNLLTNGTSIHWHGMHQKDTNLHDGA
NGVTECPIPPNGGRKVYQF
RAQQYGTSWYHSHFSAQYGNGVVGTVQINGPASRNYDIDLGVFPLMDYYHRSADELVHFTMN
NGPPFSDNVLFNGTAKHP
MTGAGEYANVTLTPGKRHRLRLINTSTENHFQVSLVNHTMTVIAADMVPVQAQTVDSLFLALG
QRYDVTIDAVSTPGNYW
FNVTYGGQAFCGGSFNPHPAAVFHYAGGPGGLPTDPGTAPEDHQCLDLNNLTPVVKRNVPVSG
FVKKPSNTLDVHLDLTG
TPLFVWKVNGSAINVDWNKPILEYVMTGNTSYPVSDNIVQVDGVNQWTYWLVENDPDGAFSVP
HPMHLHGHDFLVLGRSP
DVPPGSQQRFVFDPSTDLARLRGTNPVRRDVAMLPAKGWLLLAFRTDNPGAWLFHCHIAWHVS
GGLSVNFLERPTDLRRR
ISAADRSDFERVCREWRAYWPTNPYEKADSGLRHRFVEESEWMVEG
>XP_003654809.1:1-617 benzenediol:oxygen oxidoreductase [Thermothielavioides terrestris NRRL 8126]
MKSLAGAVALMLGILAPTITAAPPATLAQRDLLTPLEGRQDAAGLPSCNTPSNRACWTTGFDINT
DYELKTPTTNVTRKY
TLVLTEATNWKGPDGFVKERVMLVNSPTIFADWGDNLEITVINNLQTNGTSIHWHGLRQLNNNL
NDGVNGVTECPIPPKG
GKKLYRFRAVQYGTTWYHSHFSAQYGNGVVGGIQINGPASLPYDIDLGVFPITDWYNLTADQLVL
YTQNNGPPFSDNVLF
NGTAVHPFTGEGKYANVTLTPGKRHRLRLINTSVENHFQVSLVNHTMTIIAADLVPVNALTVDSL
FLGVGQRYDVTIDAS
RTPGNYWFNVTYGGSNLCGGSNNKFPAAIFHYAGAPGGLPTDPGVAPVDHNCLDNMDLTPVV
KRSVPANSFVKKPENTLD
VHLDTTGTPLFVWKVNGSAINIDWNNPVDDYVMKGNLSFPTADNIVEVDAADQWTYWLIEND
PEAVISLPHPMHLHGHDF
LVLGKSPVSPPGAQVAYTFDPSTDLARLVGTNPVRRDVTMLPAKGWLLLAFKTDNPGAWLLHCH
IAWHVSGGLSVVFLER
ASDLRAQLNGAAAAADKAEFERVCAAWRDYYPANDPFHKVDSGLKQRFVEVSEWLIR
>SPQ24255.1:1-621 fe4d1947-85b7-422d-b417-222af6121c61 [Thermothielavioides terrestris]
MKSLAGAVALMLGILAPTITAAPPATPAQRDLLTPLEGRQDAAGLPSCNTPSNRACWTTGFDINT
DYELKTPTTNVTRKY

TLVLSEATNWKGPDGFVKERVMLVNNSIVGPTIFADWGDNLEITVINNLQTNGTSIHWHGLRQL
NNNLNDGVNGVTECPI
PPKGGKKLYRFRAVQYGTTWYHSHFSAQYGNGVVGGIQINGPASLPYDIDLGVFPITDWYNLTAD
QLVLYTQNNGPPFSD
NVLFNGTAVHPFTGEGKYANVTLTPGKRHRLRLINTSVENHFQVSLVNHTMTIIAADLVPVNALT
VDSLFLGVGQRYDVT
IDASRTPGNYWFNVTYGGSNLCGGSNNKFPAAIFHYAGAPGGLPTDPGVAPVDHNCLDNMDLT
PVVKRSVPANSFVKKPE
NTLDVHLDTTGTPLFVWKVNGTAINIDWNNPVDDYVMKGNLSFPTADNIVEVDAANQWTYWL
IENDPEAVISLPHPMHLH
GHDFLVLGKSPVSPPGAQVAYKFDPSTDLARLVGTNPVRRDVTMLPAKGWLLLAFKTDNPGAWL
FHCHIAWHVSGGLSVV
FLERATDLRAQLNGAAAAADKAEFERVCAAWRDYYPANDPFHKVDSGLKQRFVEVSEWMIR
>KAK3995163.1:1-629 laccase [Cladorrhinum sp. PSN332]
MKSFIGVAALMMGILAPRGIFAAPPVTHVQRDDASRDLLVPLPLEERDSHIEPRQGLTPPSCHTAS
NRACWGSYNINTDY
EVNGPNTGATRSYTLTLTEVDNWTGPDGVVKEKVMLVNGNILGPNIEANWGDTIQVNVINNLR
TNGTTIHWHGFHQKGSN
LHDGANGVTDCPIPPKGGSRVYRFRAQQYGTSWYHSHFSAQYGNGIVGTIVIHGPASLPYDIDLG
VFPITDYYYKSADEL
VEITKNAGPPFGDNVLFKGLGKHPVTGAGQWANVTLTPGKRHRLRIINTSTENHFQVNLQNHT
MTVIASDMVPVNAQTVD
SLFLAVGQRYDVTIDANKSVGNYWFNVTFGGQAFCGGSFHPNPAAIFHYAGAPGGTPTHRGVAP
VDHQCLDLPNLTPVVQ
RTVPINNFVKKPDNTLPVHLDLTGTPLFVWKVNGSAIDVDWGKPTVDYVLSQNTSYPPQANVIT
VNSVNQWTYWLIENDP
DGAFSLPHPMHLHGHDFVVLGRSPDVPPGSQQRFVFSAADLGRLRGNNPPRRDVTMLPAKGWV
LIAFKSDNPGAWLFHCH
IAWHVSGGLSVQYLERPNDFRQQITTADRTIHDNVCNAWRAYWPTNPFPKGDSGLKVRSVGESE
DWMIK
>KAK4462624.1:1-629 laccase [Cladorrhinum samala]
MKSFISVAALMMGILAPRGLFAAPPITSVQRDLGTRDLLAPLPVEARDSHLEPRQALTPPSCHTAS
NRACWGNFNINTDY
EVNGPNTGVTRTYTLTLTEVDNWTGPDGVVKEKVMLVNGNILGPNIEANWGDNIQITVINNLRT
NGTSIHWHGFHQKDTN
LHDGANGITECPIPPKGGSRVYKFRAQQYGTSWYHSHFSAQYGNGVVGTIVVHGPASLPYDIDLG
AFPLTDYYYRSADEL
VEITKNAGPPFSDNVLFKGLGKHPVTGAGQWANLTLTPGKRHRLRIINTSTENHFQVNLQNHTM
TVIASDMVPVNAQTVD
SLFLAVGQRYDVTIDANKTPGNYWFNVTFGGQALCGGSLNPAPAAIFHYAGAPGGLPTNRGVPP
VDHQCLDLDNLTPVVQ
RTVPVNGFVKKPDNTLPVHLDLTGTPLFVWKVNGSAIDVDWSKPTIDYVLSQNTSYPPQANVITV
NTVDQWTYWLVENDP
DGAFALPHPMHLHGHDFVVLGRSPDVQPGSQQRFVFSAADLSRLRGNNPTRRDVTMLPAKGW
VLIAFKSDNPGAWLFHCH
IAWHVSGGLSVQYLERPNELRQRITPADRTMHDNNCNAWRAYWPTNPFPKGDSGLRKRSVGVS
DEWMIK
>KAJ4289950.1:1-610 laccase, multicopper oxidase, benzenediol:oxygen oxidorectuctase [Collariella sp. IMI 366227]
MKSLLSAVALMVGILAPSLVVAAPPQALSPSPRDLLAPIEERQETQGRPPPNCHTPSNRACWRPGF
NINTDYEVDTPFTG

NTRRVNNWTGPDGVVKEKVMLVNGKILGPTIFADWGDFIQVTVINNLETNGTSIHWHGMHQK
DTNLHDGANGVTECPIPP
KGGRKVYRFRAQQYGTSWYHSHFSAQYGNGVVGTIHINGPASLPYDIDLGVFPITDYYYKAADEL
VHITQNNGPPFSDNV
LFNGTNVHPTTGAGQYANVTLTPGKRHRLRIINTSTENHFQLSLVNHSMTVISADMVPVNAMTV
DSLFLAVGQRYDVTID
ASKAVGNYWFNVTFGGNALCGGSVHPAPAAVFRYAGAPNVLPTDPGAKPVDHQCLDTVGLTPV
VKRTVPVNNFVKKPSNT
LDVHLDLTGTPLFVWKVNGSAINVNWGKPVLENVMAGNLTFPREDNIVRVDGWTYWLVENDP
GAQFSIPHPMHLHGHDFL
IVGRSPDVAAGSQRFTFDPAVDLPRLKGNNPLRRDVTMLPAGGWLLLAFKTDNPGAWLFHCHIA
WHVSGGLSVDFLERPN
ELRQRITRDDRNDFNRVCREWRSYWPTNPYPKIDSGLRVRYVDEGEWLVR
>KAK4225831.1:1-630 laccase [Podospora fimiseda]
MKSFIGVAALLMGILAPRGIFAAPPVTPVQRDLAPRDLVVPLPLEERDSHLEARQAITPPSCHTASN
RACWGSFNINTDY
EVAGPNTGVVRSYTLTLTEVDNWTGPDGVVKHKVMLVNGNILGPTLEANWGDTFQINVINNLR
TNGTTIHWHGFHQKGSN
LHDGANGVTDCPIPPKGGSRIYRFRAQQYGTSWYHSHFSAQYGNGIVGTIVVHGPASLPYDIDLG
AFPITDYYYKSADEL
VELTKNAGPPFGDNVLFKGLGKHPVTGVGQYANITLTPGKRHRLRLINTSTENHFQLNLQNHSM
TIIASDMVPVQAQTVN
SVFLAVGQRLDVTIDGNQSIGNYWFNVTFGGQAFCGGSLHPNPAAIFHYAGAPGGVPTNRGVAP
VDHQCLDLPNLTPVVS
RTVPVNNFVKKVDNTLPVTLDLSGTPLFVWKVNGSAIDVDWQKPTVDFVLSQNTSYPPEANVIT
VNSVNQWTYWLIENDP
DGPFSLPHPMHLHGHDFVVLGRSPDVQPGSQQRFVFSPADLGRLRGNNPVRRDVTMLPAKGWL
LIAFKSDNPGAWLFHCH
IAWHVSGGLSVQYLERPNEFRQQISTADRTIHDNVCNAWRAYWPTNPFPKGDSGLRKRKNVGES
EEWMIR
>KAK4167045.1:1-630 laccase [Cladorrhinum sp. PSN259]
MKSFIGVAALVMGILAPHGIFAAPPITPVQRDLDTRDLLLPLPIEGRDSHIEPRQALAPPPSCHTAS
NRACWGTFNINTD
YEVNGPNTGATRSYTLTLTEVDNWTGPDGVVKRKVMLVNGNILGPTIEANWGDTIQVNVINNLR
TNGTTIHWHGFHQKGS
NLHDGANGVTDCPIPPNGGSRIYKFRAQQYGTSWYHSHFSAQYGNGVVGTIVVNGPASLPYDID
LGVLPLTDYYYRTADE
LVEITKNAGPPFSDNVLIKGVAKHPVTGAGQWANITLTPGKRHRLRIINTSTENHFQVNLQNHTM
TVIASDMVPVNAQTV
DSLFLAVGQRYDVTIDANKSVSNYWFNVTFGGQAFCGGSLNPSPAAIFHYAGAPGGTPTNRGVA
PIDHQCLDLPNLTPVV
TRTVPVTNFVKKADNTLPVHLDLGGTPLFVWKVNGSAIDVDWGKPTVDYVLSQNTSYPPQANV
ITVNAVNQWTYWLIEND
PDGGFALPHPMHLHGHDFVVLGRSPDVQPASQQRFVFSASDLANLRGNNPVRRDVTMLPAKG
WLLIAFKSDNPGAWLFHC
HIAWHVSGGLSVQYLERPNEFRQQISTADKTIHNNVCNAWRSYWPTNPFPKGDSGLKVRSVGVS
EEWMIK
>1GW0_A:1-559 Chain A, LACCASE-1 [Melanocarpus albomyces]
EPTCNTPSNRACWSDGFDINTDYEVSTPDTGVTQSYVFNLTEVDNWMGPDGVVKEKVMLINGNI
MGPNIVANWGDTVEVT
VINNLVTNGTSIHWHGIHQKDTNLHDGANGVTECPIPPKGGQRTYRWRARQYGTSWYHSHFSA
QYGNGVVGTIQINGPAS

LPYDIDLGVFPITDYYYRAADDLVHFTQNNAPPFSDNVLINGTAVNPNTGEGQYANVTLTPGKRH
RLRILNTSTENHFQV
SLVNHTMTVIAADMVPVNAMTVDSLFLAVGQRYDVVIDASRAPDNYWFNVTFGGQAACGGSL
NPHPAAIFHYAGAPGGLP
TDEGTPPVDHQCLDTLDVRPVVPRSVPVNSFVKRPDNTLPVALDLTGTPLFVWKVNGSDINVDW
GKPIIDYILTGNTSYP
VSDNIVQVDAVDQWTYWLIENDPEGPFSLPHPMHLHGHDFLVLGRSPDVPAASQQRFVFDPAVD
LARLNGDNPPRRDTTM
LPAGGWLLLAFRTDNPGAWLFHCHIAWHVSGGLSVDFLERPADLRQRISQEDEDDFNRVCDEW
RAYWPTNPYPKIDSGL
>3DKH_A:1-558 Chain A, Laccase-1 [Melanocarpus albomyces]
EPTCNTPSNRACWSDGFDINTDYEVSTPDTGVTQSYVFNLTEVDNWMGPDGVVKEKVMLINGNI
MGPNIVANWGDTVEVT
VINNLVTNGTSIHWHGIHQKDTNLHDGANGVTECPIPPKGGQRTYRWRARQYGTSWYHSHFSA
QYGNGVVGTIQINGPAS
LPYDIDLGVFPITDYYYRAADDLVHFTQNNAPPFSDNVLINGTAVNPNTGEGQYANVTLTPGKRH
RLRILNTSTENHFQV
SLVNHTMTVIAADMVPVNAMTVDSLFLAVGQRYDVVIDASRAPDNYWFNVTFGGQAACGGSL
NPHPAAIFHYAGAPGGLP
TDEGTPPVDHQCLDTLDVRPVVPRSVPVNSFVKRPDNTLPVALDLTGTPLFVWKVNGSDINVDW
GKPIIDYILTGNTSYP
VSDNIVQVDAVDQWTYWLIENDPEGPFSLPHPMHLHGHDFLVLGRSPDVPAASQQRFVFDPAVD
LARLNGDNPPRRDTTM
LPAGGWLLLAFRTDNPGAWLFHCHIAWHVSGGLSVDFLERPADLRQRISQEDEDDFNRVCDEW
RAYWPTNPYPKIDSG
>2Q9O_A:1-559 Chain A, Laccase-1 [Melanocarpus albomyces]
EPTCNTPSNRACWSDGFDINTDYEVSTPDTGVTQSYVFNLTEVDNWMGPDGVVKEKVMLINGNI
MGPNIVANWGDTVEVT
VINNLVTNGTSIHWHGIXQKDTNLHDGANGVTECPIPPKGGQRTYRWRARQYGTSWYHSHFSA
QYGNGVVGTIQINGPAS
LPYDIDLGVFPITDYYYRAADDLVHFTQNNAPPFSDNVLINGTAVNPNTGEGQYANVTLTPGKRH
RLRILNTSTENHFQV
SLVNHTMTVIAADMVPVNAMTVDSLFLAVGQRYDVVIDASRAPDNYWFNVTFGGQAACGGSL
NPHPAAIFHYAGAPGGLP
TDEGTPPVDHQCLDTLDVRPVVPRSVPVNSFVKRPDNTLPVALDLTGTPLFVWKVNGSDINVDW
GKPIIDYILTGNTSYP
VSDNIVQVDAVDQWTYWLIENDPEGPFSLPHPMHLHGHDFLVLGRSPDVPAASQQRFVFDPAVD
LARLNGDNPPRRDTTM
LPAGGWLLLAFRTDNPGAWLFHCHIAWHVSGGLSVDFLERPADLRQRISQEDEDDFNRVCDEW
RAYWPTNPYPKIDSGL
>KAK4183168.1:19-629 laccase [Podospora australis]
SIFAAPPVTPIQRDLALTTTVEKRGADTNLLRALPVEGRQASCHSATNRACWKTGFTINTDYETST
PTTGVTRTYTLTLT
EVDNWTGPDGVVKKKVMLVNGGILGPTILADWGDNIQVTVINNLRTNGTSIHWHGMHQKDTN
LHDGANGVTECPIPPKGG
TRVYKFKAQQYGTSWYHSHFSAQYGNGVVGTILINGPASLPYDIDLGVFPITDYYYRTADELVEFT
MNNGPPASDNVLFQ
GKGKHPVTGAGQWANVTLTPGKRHRLRIINTSTHDHFQVKLQNHTMTVIASDMVPVNAFTVDS
LFLAVGQRYDVTIDANK
SVGNYWFNVTYGGAAFCGVSNNPNPAAIFHYAGAPGGVPTNRGTPSIDHQCADLPNLTPVVTR
TVPVSGFTKKADNTLPV

TLDLSGTPLFVWKVNGSAINVQWEKPIIDYVLAQNTSYPREANVITINSVNQWTYWLIENDPDGA
FSLPHPMHLHGHDFV
VLGRSPTVNPALQQRFVFGPSDVANLKSVNPVRRDVTMLPARGWVLIAFKSDNPGAWLFHCHIA
WHVSGGLSVDYLERPN
DLRQRITTADRNMHNNNCNAWRAYWPTNPFPKIDSGLRKKSVGESKEWFIK
>XP_006692507.1:2-607 hypothetical protein CTHT_0020300 [Thermochaetoides thermophila DSM 1495]
KFLTYATTLLGTLTAVVGAVPTRSGTKNKYIRDGPGPCHTPSNRACWASGFDIYTDYEVNTPNTGV
TRKYTLTLTEEDNW
TGPDGVVKEKIMLVNGKIMGPTIEANWGDWIEVNVINNLLTNGTSIHWHGIHQKGSNLHDGAN
GVTECPIPPNGGQRTYR
FRAQQYGTSWYHSHFSAQYGNGIVGPIVIHGPASLPYDIDLGPFPLVDYYYKSADELVHHTQSNG
PPFSDNVLFNGTGVH
PQTGHGQYAKVTLTPGKRHRLRIINMSTENHFQVSLVGHQFTVIAADMVPVHSYNTDSLFLAVG
QRYDVIIDASPTPGNY
WFNVTFGGGFACGGSLNPHPAAIFHYEGAPDALPTNPGVTPRDHNCLDTLDLVPVVPRNVQVN
QFVKKPENTLPVELSIG
GTPLFVWKVNGSAIDVDWGNPVLQYVMDGNTSYRQADNIVEVNGVNQWTYWLIENDPNGAF
SLPHPMHLHGHDFLIVGRS
PDVPPGSNQRYNFDPATDIYRLRGQNPTRRDVAMLPAGGWLLLAFRTDNPGAWLLHCHIAWHV
SGGLSVDFLERPDDLRN
SIPQHDKDEFNRVCNEWRTYWPNNPYPKIDSGLKHRWVEESEWLVR
>KAK4204568.1:1-619 Cupredoxin [Triangularia verruculosa]
MKSFFSAAALLLGLIIPSAVLAAPPLPGVPREVTRDLLRPVEERQSSCHTPSNRACWATGFDINTDY
EVSTPNTGVTRTY
TLTLSEVDNWLGPDGVVKQKVMLVNGDIFGPTITANWGDWIQVNVINNLRTNGTSIHWHGLH
QKGTNMHDGANGVTECPI
PPKGGSRIYKFRAQQYGTSWYHSHFSAQYGNGVVGTIVINGPASLPYDIDLGVFPITDYYHKPADV
LVEETMNGGPPPSD
TVLFKGQGKNPATGAGKFANITLTPGKRHRLRIINTSTHDHFQLKLQNHTMTIIAADMVPVQAQ
TVDSLFLAVGQRYDVT
IDANKSVSNYWFNATFGGGLACGASLNPHPAAIFRYQGAPNANPTNQGTPAADANCLDLNNLT
PVVSRSVPTAGFTPRPN
NTLPVSLQLGGTPLFVWKVNGSSINVDWDKPIVDYVIAQNTSYPPQANVITVNTVNQWTYWLVE
NDPTGPFSIPHPMHLH
GHDFLVVGRSPDQPAGGPQTRYRFDPATDMARLKGSNPVRRDVAVLPANGWLLIAFKSDNPGA
WLFHCHIAWHVSGGLSV
QYLERPNDLRNGFTTADKNQHNNNCNAWRAYWPTNPFPKIDSGLKVRKWVGEHPDWYIK
>KAK3312917.1:1-630 laccase-1 [Apodospora peruviana]
MRFTFGAFALLAGSLVFGAPPGNPQQRGLLTLLETRDVAPGEHVDLDLEARADDTSGNLVARAP
TCNTPSNRACWSTGFN
INTDYETSTPTTGVTKFYTLTLTEVDNWVGPDGVTKKKVMLVNGKILGPTIVADWGDFIQITVINN
LRTNGTSIHWHGMR
QFGSNLQDGANGVTECPIPPKGGSKIYKFRASQYGTSWYHSHFSAQYGNGVVGSMQINGPASLP
YDIDLGVFPITDYYYR
TADELVLFTENNGAPFSDNVLFNGTNVHPVTGVGKYANVTLTPGKRHRLRLINPSTENHFQLSLV
GHDMTIISSDLVPVN
AMTVSSVFLGVGQRLDVTIDASKTPGNYWFNVTFGGQNFCGGSQNPTPAAIFHYAGAPGGLPTN
KGVAPVDHQCLDLLNL
TPVVTRSVTPGGFNPNPGNTLPVTIDLSGTPLFVWKVNGSAINVDWNKPVLDYVLTGNTSYPPRN
NLIQVNSVNQWTYWL

IENDPDGPFSLPHPIHLHGHDFLIVGRSPDVTPGSQTRFKFNAATDNARLNGSNPARRDVAMLPA
KGWLLIAFKTDNPGA
WLMHCHIAWHVSGGLSVDFLERPADLKAGISAADKAAFNQNCAAWRAYFPSQDPFPKIDSGLK
MMKHKYV
>KAK0724512.1:1-616 laccase-1 [Lasiosphaeris hirsuta]
MKFFASFVALAAGILAPSAVLAAPPVTPVQRDTLGVGSDLSVVSKRATNCNSATNRACWTTNPN
FNINTDYETSTPTTGV
IRRYTLNLTETDNFIGVDGVVKEKVMLVNNKILGPTIFADWGDNIEILVINNLRTNGTSIHWHGIR
QLNSNLHDGANGVT
ECPIPPNGGRFTYKFRATQYGTSWYHSHFSAQYGNGVVGTIQINGPASLPYEVDLGVFPISDYYYR
TADQLVEFTMNNGA
PFSDNVLFNGTAKHPTTGVGAYANVTLTPGKRHRLRIINTSTENHFQLSLVGHQFTIISSDFVPVQA
QTVDSLFLAVGQR
YDVTIDASKTPGNYWFNATFGGQNFCGGSFNPAPAAIFHYAGAAGGLPTDPGTAPVDSQCLDLL
NLTPVVTRTVPVANFS
KRPANTLPVTLTIGGTPLFVWKVNGSAINVDWNKPVAEYILTSNTSYPTSENIVQIDVADQWTYW
LIENDPDGIVSLPHP
FHLHGHDFVILGRSPDELPANQVRHVFNETTDRGLLRGTNPARRDVVMLPARGWALIAFKSDNP
GAWLMHCHIAWHVSGG
LSVDFLERPVEFKNRITPADAAAFNQNCAAWRAYFPALDPFPKIDSGLRKSKFIKE
>KAK3317472.1:1-621 laccase-1 [Cercophora scortea]
MRSFIGAVALMAGVLAPRLVLGAPPVTPMQRDVLRAVEERDVSASSSGVDLYPRASTCNTPSNRA
CWSPGFDINTDYETS
IPTTGVTRNYVLTLSEVDNWTGPDGVVKSKVMLINGKILGPTLVADWGDFFQITVINNLRTNGTSI
HWHGIRQLGSNLHD
GANGVTECPIPPKGGSKIYKFRAQQYGSSWYHSHFSAQYGNGVVGAIQINGPASLPYDIDLGPMP
LSDYYYRTADEVVIF
TENNGAPASDNVLFNGFGKHPTTGAGQYANITLTPGKRHRLRLINTSTENHFQVSLANHTFTIIAS
DFVPVQAQTVDSVF
LGVGQRMDVTIDASKAIGNYWLNVTFGGQGFCGSSNNPAPAAIVHYAGASGGLPTNPGVAPVD
AQCMDLLNLTPVVQRTV
PTTFNKTPGNTMPVTIDLSGATGKLFVWKVNGSAINVDWNKPIVDYVLTGNTSYPTNENLVKID
AVNQWTFWLIENDPNG
PFSLPHPIHLHGHDFVVLGRSPLATPASQTTYIFDPATDGANLDGSNPMRRDVTMLPAKGWLLIA
FKTDNPGSWLMHCHI
AWHVSGGLSVDFLERAADFRAGISPADALAFNTNCAAWRAYFPSADPFPKIDSGLRHKYVR
>KAK3689317.1:1-612 laccase [Podospora appendiculata]
MRSFIGAVALLAGMFAPRVALGAPPVTPMQRDVLRMVEERDIGVDLAPRASTCNTPSNRACWSS
GFDINTDYETSIPTTG
VTRNYVLTLTEVDNWTGPDGVVKSKVMLINGPTLVADWGDFFQITVINKLRTNGTSIHWHGIRQ
QGSNLHDGANGVTECP
IPPNGGSKVYKFRAQQYGSSWYHSHFSAQYGNGVVGTIQINGPASLPYDIDLGPLALTDYYYRTA
DELVVFTENNGAPAS
DNVLFNGFAKHPTTGAGQYANITLTPGKRHRLRLINTSTENHFQVSLANHTFTVIASDFVPVQAQ
TVDSVFLGVGQRMDV
TIDANKSVGNYWLNVTFGGQGFCGSSNNPAPAAVVRYAGAPGGLPTFPGAAPVDHQCLDLINLT
PVVQRTVPTTFNKQPS
NTLPVTIDLSGATGKLFSWKVNGSAININWNKPVVDYVLTGNTSYPTSENLVQITAVNQWTFWLI
ENDPNGPFSLPHPIH
LHGHDFVVLGRSPPATPASQTTYIFNPATDGANLDGSNPLRRDVAMLPAKGWMLIAFKSDNPGS
WLMHCHIAWHVSGGLS
VDFLERPSDFRAGISPADALAFNTNCAAWRAYFPTLDPFPKIDSGLRHKYMR

>KAK4107780.1:1-617 multicopper oxidase [Canariomyces arenarius]
MKSWAAAVALMVGILSPHAAAAPPANPVQRDMLQVLEARQSGPTCNTPSNRACWTNGFDINT
DYEVSTPNTGRTVAYQLT
LTEKENWIGPDGVLKNVVMLVNDKIIGPTIRANWGDNIEVTVINNLKTNGTSMHWHGLRQLGN
VFNDGANGVTECPIPPK
GGRKTYKFRATQYGTSWYHSHFSAQYGNGVVGTIQIDGPASLPYDIDLGVFPLMDYYYRSADELV
HFTQSNGAPPSDNVL
FNGTARHPETGAGQWYNVTLTPGKRHRLRIINTSTDNHFQVSLVGHNMTVIATDMVPVNAFTVS
SLFLAVGQRYDVTIDA
NSPVGNYWFNVTFGDGLCGSSNNRFPAAIFRYQGAPATLPTDQGLPVPNHMCLDNLNLTPVVTR
SAPVNNFVKRPSNTLG
VTLDIGGTPLFVWKVNGSAINVDWGKPILDYVMSGNTSYPVSDNIVQVDAVDQWTYWLIENDPT
NPIVSLPHPMHLHGHD
FLVLGRSPDELPSAGVRHIFDPAKDLPRLKGNNPVRRDVTMLPAGGWLLLAFKTDNPGAWLFHC
HIAWHVSGGLSVDFLE
RPNDLRTQLNSNAKRADRDDFNRVCREWNAYWPTNPFPKIDSGLRHRFVEESEWMVR
>VBB80813.1:2-620 Laccase-2 precursor [Podospora comata]
MKSFFSAAALLLGLVAPSAVLAAPSLPGVPREVTRDLLRPVEERQSSCHTAANRACWAPGFDINT
DYEVSTPNTGVTRTY
TLTLTEVDNWLGPDGVVKEKVMLVNGDIFGPTITANWGDWIQVNVINNLRTNGTSIHWHGLHQ
KGTNMHDGANGVTECPI
PPKGGSRIYRFRAQQYGTSWYHSHFSAQYGNGVVGTIVVNGPASLPYDIDLGVFPITDYYHKPAD
VLVEETMNGGPPPSD
TVLFKGHGKNPQTGAGNFANVTLTPGKRHRLRIINTSTHDHFQLKLQNHTMTIIAADMVPVQA
QTVDSLFLAVGQRYDVT
IDANKSVGNYWFNATFGGGLACGASLNPHPAAVFRYQGAPNTLPTNIGTPAADANCMDLNNL
TPVVSRSVPTSGFTPRPN
NTLPVSLTLGGTPLFVWKVNGSSINVDWDKPIVDYVIAQNTSYPPQANVITVNSVNQWTYWLIE
NDPTGPFSIPHPMHLH
GHDFLVVGRSPDQQAGVPQTRYRFNPATDMALLKSSNPVRRDVAMLPANGWLLIAFKSDNPGA
WLFHCHIAWHVSGGLSV
QYLERPNDLRNGFSQADKNQHNNNCNAWRAYWPTNPYPKIDSGLKVKKWVGEHPDWYIK
>KAK4641513.1:2-620 laccase, multicopper oxidase, benzenediol:oxygen oxidorectuctase [Podospora bellae-mahoneyi]
MKSFFSAAALLLGLVAPSAVLAAPSLPGVPREVTRDLLRPVEERQSSCHTAANRACWAPGFDINT
DYEVSTPNTGVTRTY
TLTLTEVDNWLGPDGVVKQKVMLVNGDIFGPTITANWGDWIQVNVINNLRTNGTSIHWHGLH
QKGTNMHDGANGVTECPI
PPKGGSRIYRFRAQQYGTSWYHSHFSAQYGNGVVGTIVVNGPASLPYDIDLGVFPITDYYHKPAD
VLVEETMNGGPPPSD
TVLFKGHGKNPQTGAGKFANVTLTPGKRHRLRIINTSTHDHFQLKLQNHTMTIIAADMVPVQA
QTVDSLFLAVGQRYDVT
IDANQSVGNYWFNATFGGGLACGASLNPHPAAVFRYQGAPNTLPTYIGTPAADANCMDLNNLT
PVVSRSVPTSGFTPRPN
NTLPVSLTLGGTPLFVWKVNGSSINVDWDKPIVDYVIAQNTSYPPQANVITVNSVNQWTYWLIE
NDPTGPFSIPHPMHLH
GHDFLVVGRSPDQPAGVPQTRYRFNPATDMALLKSSNPVRRDVAMLPANGWLLIAFKSDNPGA
WLFHCHIAWHVSGGLSV
QYLERPDDLRNGFSQADKNQHNNNCNAWRAYWPTNPYPKIDSGLKVKKWVGEHPDWYIK
>KAK4652669.1:40-658 laccase, multicopper oxidase, benzenediol:oxygen oxidorectuctase [Podospora pseudocomata]

MKSFFSAAALLLGLVAPSAVLAAPSLPGVPREVTRDLLRPVEERQSSCHTAANRACWAPGFDINT
DYEVSTPNTGVTRTY
TLTLTEVDNWLGPDGVVKQKVMLVNGDIFGPTITANWGDWIQVNVINNLRTNGTSIHWHGLH
QKGTNMHDGANGVTECPI
PPKGGSRIYRFRAQQYGTSWYHSHFSAQYGNGVVGTIVVNGPASVPYDIDLGVFPITDYYHKPAD
VLVEETMNGGPPPSD
TVLFKGHGKNPQTGAGKFANVTLTPGKRHRLRIINTSTHDHFQLKLQNHTMTIIAADMVPVQA
QTVDSLFLAVGQRYDVT
IDANKSVGNYWFNATFGGGLACGASLNPHPAAVFRYQGAPNTLPTNIGTPAADANCMDLNNL
TPVVSRSVPTSGFTPRPN
NTLPVSLTLGGTPLFVWKVNGSSINVDWDKPIVDYVIAQNTSYPPQANVITVNSVNQWTYWLIE
NDPTGPFSIPHPMHLH
GHDFLVVGRSPDQPAGVPQTRYRFNPATDMALLKSSNPVRRDVAMLPANGWLLIAFKSDNPGA
WLFHCHIAWHVSGGLSV
QYLERPNDLRNGFSQADKNQHNNNCNAWRAYWPTNPFPKIDSGLKVKKWVGEHPDWYIK
>XP_001904217.1:2-620 uncharacterized protein PODANS_5_1200 [Podospora anserina S mat+]
MKSFFSAAALLLGLVAPSAVLAAPSLPGVPREVTRDLLRPVEERQSSCHTAANRACWAPGFDINT
DYEVSTPNTGVTRTY
TLTLTEVDNWLGPDGVVKQKVMLVNGDIFGPTITANWGDWIQVNVINNLRTNGTSIHWHGLH
QKGTNMHDGANGVTECPI
PPKGGSRIYRFRAQQYGTSWYHSHFSAQYGNGVVGTIVVNGPASVPYDIDLGVFPITDYYHKPAD
VLVEETMNGGPPPSD
TVLFKGHGKNPQTGAGKFANVTLTPGKRHRLRIINTSTHDHFQLKLQNHTMTIIAADMVPVQA
QTVDSLFLAVGQRYDVT
IDANKSVGNYWFNATFGGGLACGASLNPHPAAVFRYQGAPNTLPTNIGTPAADANCMDLNNL
TPVVSRSVPTSGFTPRPN
NTLPVSLTLGGTPLFVWKVNGSSINVDWDKPIVDYVIAQNTSYPPQANVITVNSVNQWTYWLIE
NDPTGPFSIPHPMHLH
GHDFLVVGRSPDQPAGVPQTRYRFNPATDMALLKSSNPVRRDVAMLPANGWLLIAFKSDNPGA
WLFHCHIAWHVSGGLSV
QYLERPNDLRNGFSQADKNQHNNNCNAWRAYWPTNPFPKIDSGLKVKKWVGEHPDWYIK
>KAK4178952.1:1-619 Cupredoxin [Podospora setosa]
MKSFFSAAALLLGLVAPSAVLAAPPLPGVPREVTRNLLRPVEERQSSCHTASNRACWAPGFNINT
DYEVSTPNTGVTRTY
TLTLTEVDNWLGPDGVVKQKVMLVNGDIFGPTITANWGDWIQVNVINNLRTNGTSIHWHGMH
QKGTNMHDGANGVTECPI
PPKGGSRVYRFRAQQYGTSWYHSHFSAQYGNGVVGTIVVNGPASLPYDIDLGVFPITDYYHKPAD
VLVEETMNGGPPPSD
TVLFKGHGKNPATGAGQFANITLTPGKRHRLRIINTSTHDHFQLKLQNHTMTIIAADMVPVQAQ
TVDSLFLAVGQRYDVT
IDANKSVGNYWFNATFGGGLACGASLNPHPAAIFRYQGAPNANPTNQGTPAADANCMDLNNL
TPVVTRSVPTSGFTPRPN
NTLPVSLTLGGTPLFVWKVNGSSINVDWDKPIVDYVIAQNTSYPPQANVITVNSVNQWTYWLVE
NDPTGPFSIPHPMHLH
GHDFLVVGRSPDQPAGVPQTRYRFNPATDMALLKSSNPVRRDVAMLPANGWLLIAFKSDNPGA
WLFHCHIAWHVSGGLSV
QYLERPNDLRNGFSTADKNQHNNNCNAWRAYWPTNPFPKIDSGLKVRKWVGEHPDWYIK
>KAK0710528.1:1-619 Cupredoxin [Apiosordaria backusii]
MKSFFSAAALLLGLLAPSAVLAAPPVPGVPREVTRDLLRPVEERQSSCHTPSNRACWSTGFNINTD
YEVSTPNTGVTRTY
TLILSEVDNWLGPDGVVKQKVMLVNGDIFGPTITANWGDWIQVNVINNLRTNGTSIHWHGLHQ
KGTNMHDGANGVTECPI

PPKGASRVYRFRAQQYGTSWYHSHFSAQYGNGVVGTIVINGPASLPYDIDLGVFPITDYYHKPAD
VLVEETMNGGPPPSD
TVLFKGHGKNPATGAGRFANVTLTPGKRHRLRIINTSTHDHFQLKLQNHTMTIIAADMVPVQAY
TVDSLFLAVGQRYDVT
IDANKSVGNYWFNATFGGGLACGASLNPHPAAIFRYQGAPNANPTNQGTPAADANCMDLNNL
TPVVTRNVPTSGFTPRPN
NTLPVSLTLGGTPLFVWKVNGSAINVDWDKPIVDYVIAQNTSYPPQANVITVNSVNQWTYWLVE
NDPDGPFSIPHPMHLH
GHDFLVVGRSPDQPAGGPQTRYRFNPATDMARLKGSNPVRRDVAMLPANGWLLIAFKSDNPGA
WLFHCHIAWHVSGGLSV
QYLERPNDLRNGFSQADKNQHNNNCNAWRAYWPTNPFPKIDSGLKVKKWVGEHPDWYIK
>KAK0660873.1:1-619 Cupredoxin [Cercophora samala]
MKSFFSAAALLLGLAAPTAVLAAPPLPGVPREVTRDLLRPVEVRQSSCHSASNRACWAPGFDINT
DYEVSTPNTGVTRTY
TLTLTEVDNWLGPDGVVKQKVMLVNGDIFGPTITANWGDWIQVNVINNLRTNGTSIHWHGMH
QKGTNMHDGANGVTECPI
PPNGASRIYKFRAQQYGTSWYHSHFSAQYGNGVVGTIVVNGPASLPYDIDLGVFPITDYYHKPAD
VLVEETMNGAPPPSD
TVLFKGHGKNAQTGGGKFANVTLTPGKRHRLRIINTSTHDHFQLKLQNHTMTIIAADMVPVQA
QTVDSLFLAVGQRYDVT
IDANKSVGNYWFNATFGGGLACGASLNPHPAAVFRYQGAPNTLPTNQGTPAADANCMDLNNL
TPVVTRNVPTSGFTPRPN
NTLPVSLTLGGTPLFVWKVNGSSINVDWDKPIVDYVIAQNTSYPPQANVITVNSVNQWTYWLIE
NDPTGPFSIPHPMHLH
GHDFLVVGRSPDQPAGVPQTRYRFNPATDMSLLKSSNPVRRDVAMLPANGWLLIAFKSDNPGA
WLFHCHIAWHVSGGLSV
QYLERPNDLRNGFTQADKNQHNNNCNAWRAYWPTNPFPKGDSGLKVKKWVGEHPEWYIK
>KAK3348695.1:1-616 laccase [Lasiosphaeria hispida]
MKFLAGLVALAAGILAPGAVLAAPPVTPIQRDTLAVDSDLSVVNKRATNCHSATNRSCWTTNPN
FNINTDYETSTPTTVG
VRKYNLTLTEEDNWMGPDGVVKEKVMLVNGKILGPTIFADWGDKIEILVINNLRSNGTSIHWHG
LHQRGSNLADGANGVS
ECPIPPNGGRFTYKFRATQYGTSWYHSHFSAQYGNGVVGTIQINGPASLPYEVDLGVFPVSDYYYK
GADELVHFTMSNGA
PFSDNVLFNGTGKHPVTGVGSYANVTLTPGKRHRLRLINTSTENHFQLSLVGHQFTIIAADLVPVQ
AQTVDSIFLGVGQR
YDVTIDASQTPGNYWFNATFGGQAFCGGSFNPAPAAIFHYAGAAGGLPTNPGTPPVDAQCQDL
LNLTPVLTRSVPVANFV
KRPANTLPVTLQIGGTPLFVWKVNGSAINVDWNKPVVDYILTGNTSYPTSENIVQIDVANQWTY
WLIENDPDGVVSLPHP
FHLHGHDFVVLGRSPDELPASQVRHVYNDATDRPNLRGTNPTRRDVVMLPVRGWVLIAFKTDN
PGAWLMHCHIAWHVSGG
LSVDFLERPVDFKNSITPEQKTAFNQNCAAWNAYFPANAPFPKIDSGLRKSKYIKE
>KAK3936690.1:1-605 laccase [Diplogelasinospora grovesii]
MRFLLGAAALVAVIFAPAVHGAPPVSPHDMSLRDVDMDLAPRASTCNTPSNRACWTTGFDISTD
YETSTPVTGVTRTYTL
TITEVDNWVGPDGVTKEKVMLINNKILGPTITADWGDRIQVTVINNLRTNGTSIHWHGLRQLGS
NLHDGANGLTECPIPP
NGGSKVYSFLATQYGTSWYHSHFSAQYGNGVVGTILINGPASLPYDIDLGTFPLSDYYYETADKIV
LNTATAGPPPSSNV
LFNGTNVHPVTGQGQYANVTLTPGKRHRLRLINTSVENHFQVSLVGHSMTIIASDLVPQNAMTV
QSLFIAVGQRYDVTID

ASQAVGNYWFNVTFGGSGFCGTSQNPTPAAIFHYAGASGQLPTNPGTAPADSNCLDLLNLTPVV
TRTVPVANFAPQPNNT
IPIHLDNSVVPFLTWKVNGSAINVQWEKPVIDYILTGNTSYPAGENIIQVNSVNQWAYWLIENDPT
GAFSLPHPIHLHGH
DFVILGRSPDATPASQTQYIFNPSTDIFRLNGNNPARRDVAMLPAKGWLLLAFQTDNPGAWLMH
CHIAWHVSHGLSVDFL
ERPTDLKNSISAADQANFNNNCANWRAYWPSQDPFPKVDSGLRMR
>3PPS_A:1-604 Chain A, Laccase [Canariomyces arenarius]
MKSWAAAVALMVGILSPHAAAAPPANPVQRDMLQVLEARQSGPTCNTPSNRACWTNGFDINT
DYEVSTPNTGRTVAYQLT
LTEKENWIGPDGVLKNVVMLVNDKIIGPTIRANWGDNIEVTVINNLKTNGTSMHWHGLRQLGN
VFNDGANGVTECPIPPK
GGRKTYKFRATQYGTSWYHSHFSAQYGNGVVGTIQIDGPASLPYDIDLGVFPLMDYYYRSADELV
HFTQSNGAPPSDNVL
FNGTARHPETGAGQWYNVTLTPGKRHRLRIINTSTDNHFQVSLVGHNMTVIATDMVPVNAFTVS
SLFLAVGQRYDVTIDA
NSPVGNYWFNVTFGDGLCGSSNNKFPAAIFRYQGAPATLPTDQGLPVPNHMCLDNLNLTPVVT
RSAPVNNFVKRPSNTLG
VTLDIGGTPLFVWKVNGSAINVDWGKPILDYVMSGNTSYPVSDNIVQVDAVDQWTYWLIENDPT
NPIVSLPHPMHLHGHD
FLVLGRSPDELPSAGVRHIFDPAKDLPRLKGNNPVRRDVTMLPAGGWLLLAFKTDNPGAWLFHC
HIAWHVSGGLSVDFLE
RPNDLRTQLNSNAKRADRDDFNRVCREWNAYWPTNPFPKIDSGL
>XP_060293879.1:1-618 laccase-1 [Lasiosphaeria miniovina]
MKSIIGAAALLLGLLAPSAVLGAPPVTPIQRDLLTPIEERDESGVDTLRHRASTCNTPSNRACWSPG
FDINTDYEVSTPT
TGVTRQYTLTFTEVTNWVGPDGVVEPIRMLVNGKILGPTIVADWGDNIQVTVINNLVTNGTSVH
WHGLHQRTSVLHDGAN
GVTECPLPPKGGSKVYKFRATQYGSSWYHSHFSAQYANGVVGTILINGPASLPYDIDLGVFPLSDY
YYQSADDLVIQTKT
QGAPPSSNVLFNGTAKHPVTGAGSYANVTLTPGKRHRLRLINTSAENHFQVSLVGHSMTVIATDL
VPVNAFTTNSLLLGV
GQRYDVTIDASQTPGNYWFNVTFGGSGFCGTSANPFPAAIFHYAGAPGGLPTAQGTATDHNCLD
NLNFRPVVQRNVVVSN
FTKRPANTLPVTLEGPPGQPLFVWKVNGSSINVDWNKPVAEYVLTGNTSYPTSENLVILDQANV
WTYWLIENDPDGAFSL
PHPFHLHGHDFLVLGRSPDVTPASQTRFHFSQSDVASLNGSNPVRRDVAMLPAGGWLLIAFHTN
NPGAWLMHCHIAWHVS
GGLSVDFLERRADFRSQTSAADAAAFNTNCNNWRNYFPAQDPFPKIDSGLRKAKYMRE
>KAK3358324.1:1-618 laccase-1 [Lasiosphaeria ovina]
MKSIIGAAALLLGLLAPSAVLGAPPVTPIQRDLLTPIEERDESRVDTLRHRASTCNTPSNRACWSSG
FNINTDYEVSTPT
TGVTRQYTLTFTEVTNWVGPDGVVEPIRMLVNGKILGPTIVADWGDNIQVTVINNLVTNGTSVH
WHGLHQRTSVLHDGAN
GVTECPIPPKGGSKVYKFRATQYGTSWYHSHFSAQYANGVVGTILINGPASLPYDIDLGVFPLSDY
YYQSADDLVILTKT
QGAPPSSNVLFNGTAKHPVTGAGSYANVTLTPGKRHRLRLINTSAENHFQVALVGHSMTVIATDL
VPVNAFTTNSLLLGV
GQRYDVTIDASQTPGNYWFNVTFGGSGFCGTSANPFPAAIFHYAGAPGGLPTAKGTATDHNCLD
NLNFKPVVQRNVVVSN
FTKRPANTLPVTLEGPPGQPLFVWKVNGSSINVDWNKPVAEYVLTGNTNYPTSENLVILDQANV
WTYWLIENDPDGAFSL

PHPFHLHGHDFLVLGRSPDVTPASQTRFHFSQSDVASLNGSNPVRRDVAMLPTGGWLLIAFQTN
NPGAWLMHCHIAWHVS
GGLSVDFLERRADFKAQTSAADTAAFNSNCNKWRAYFPAQDPFPKIDSGLRKAKYMRE
>KAK3385816.1:1-623 laccase [Podospora didyma]
MRSFISTAALVLGLLAPSAVLAAPPITPVQRDILTPISEVELRAIEARHKDSAEGLSPRASTCNTATN
RACWTTGFNINT
DYETSTPTTGVIRPYTLTLTETTNWTGPDGVVKPFTMLINGGILGPTITADWGDTIQVTVINNLVTN
GTSMHWHGMHQRN
SNLHDGANGVTECPIPPKGGTHVYRFLATQYGTSWYHSHFSAQYGNGVVGPIVINGPASLPYDID
LGPLALSDYYYKSAD
ELVEFTKTNGPPASDNVLFNGLAKHPITGAGNYATLTLTPGKRHRLRIINTSTENHFQVSLVNHSM
TIIASDLVPVNAQT
VSSLFLAVGQRYDVTIDANQAVGNYWLNVTFGGQGLCGTSKNPNPAAIIKYTGAPTGLPTNKGG
PVTDHQCLDLQNLSPV
LTRSLTVSNFTKRPANTLPVTLEGPPNTPLFVWKVNGSAINVDWNKPVVDYVASGNTSYPTSENI
VVVPDANIWTYWLIE
NDPDGGFSLPHPIHLHGHDFLVLGRSPDIQPLSQQRFKFTAADIPSLKGTNPARRDVTMLPAGGW
LLIAFKTDNPGAWLM
HCHIAWHVSGGLSVDFLERPVDFRARIPPADLLAHNANCNAWRAYFPALDPHPKGDSGLRMMY
>KAK1753342.1:1-599 benzenediol:oxygen oxidoreductase [Echria macrotheca]
MKFLNGAVALLSGLLAPSALAAPPMQRDVELEMRNEIEPRVAPTCNTPSNRACWTTGFNINTDY
ETSTPPSGSTRRYTLN
ITEADNFVAPDGSIKPKAMLVNGPTIFADWGDNIEVTVINNLRTNGTAIHWHGLQQKTTQLYDG
ANGVTQCPIPPKGGSM
VYKFRATQYGTSWYHSHFSAQYSNGVVGTIQINGPASLPYDIDLGVFPISDYYLKSADELIIITQNA
GPPPSDNVFLNGT
GKHPTTGQGQYANVTLTPGKRHRLRLINTSAENHFQLSLVGHQMTIIASDFVPVNAQTVSSVFLG
VGQRYDVTIDASQTP
GNYWFNVTFGGEGFCGSSNNPAPAAIFHYAGAPGGLPTNPGTPPTDSQCLDIINLTPVVTRTVPT
VNFVKRPSNTLPVHL
QFGGTPLFVWQVNGSAINVDWNKPVAEYVMTNNQSYPTSENLVIVDAVDQWTYWLVENDDPT
GIVALPHPFHLHGHDFVV
LGRSPDVTPASQTPFVFNQTTDLSRLNGNNPTRRDVTMLPARGWILIAFKTNNPGAWLMHCHIA
WHVSGGLSVDFLERRT
DFKNQISSADATVFNNNCNAWRSYFPSQDPFPKIDSGLK
>KAK4210240.1:1-610 laccase [Rhypophila decipiens]
MKFSFGALAVLAGIFAPGNVMGAPPKTPQQRDLLQLLETRDIDQVESRDVSLVKRAPTCNTPSNR
ACWSTGFNINTDFET
SWPTTGVTKTYTLTLTEVDNWTGPDGVVKRKVMLVNNNIVGPTIMADWGDFIQITVINNMISNG
TSIHWHGMHMRGTNLH
DGANGVTECPVPPGKSKVYRFHATQHGTSWYHSHFSAQYGNGIVGSIQINGPASLPYDVDLGVF
PISDYYYRTADEIIEA
GGAPPPSDNILFNGTAKHPTTGAGKWYNVTLTPGKRHRLRIINPSAENHYQVSLVGHKFTIIASDL
VPVNAQTVDDLFLG
VGQRYDVTIDANQSVGNYWFNVTFGGQGFCGTSRNPNPAAVFRYAGAPATLPTNQGTPPTDHQ
CLDLINLTPVVSRTITP
STFSPTPGNTLPVVFDPAGGPIFIWKVNGTAINVDWNKPVLEYVITGNTSYPPAENLVHINQTNQ
WVYWLIENDPDGLFS
LPHPIHLHGHDFLIVGRSPDSPPGNQVRYKFNPAVDNGRLISNNPGRRDVAMLPARGWMLIAFK
TDNPGAWLMHCHIAWH
VSGGLSVDFLERVTDLRNGLTTQDKNEFNQNCNDWRAHTPLYPKIDSGLR
>AUS45857.1:1-591 multicopper oxidase [Coniochaeta hoffmannii]

MKFFPVSIVALFTFFSSTAVKAAPSSFKELDARASTCNTPSNRACWTTGFDINTDYETNTPTTGVTR
NYALTITEVDNWT
GPDGVVKPKVMLVNGPTIFADWGDRVNITVTNNLRTNGTSIHWHGLRQFGSNLQDGANGVTE
CPIPPKGGSKVYSFMATQ
YGTSWYHSHFSAQYGNGVVGTIQINGPASLPYDVDLGVFPITDYYYQTADDLVEFTKNNGAPASD
NILINGTNVHPTTGV
GKYANVTLTPGKRHRLRIINTSVENHFQVSLVNHSMTVIAADMVPVNAYTTDTLFLGVGQRYDV
TIDASKAVGNYWFNVT
LPPNGFCGTSNNQFPAAVFHYAGASGGLPTARGTPPVDTLCYDNIDLVPVVSRTASPGNFNPSPN
NSLPVHLDTTGSPLF
VWQINGSSINVDWNKPVLQYVIENNTSYPPSENIVSVPSNNQWSYWLIENDVDAVFSLPHPFHLH
GHDFLVLGRSPVASP
AFPQTRYKFDPATDLALLKGNNPVRRDVAMLPAKGWLLIAFKTDNPGAWLMHCHIAWHVSGG
LSVDFLEGVSTLRNNLPP
ADKAAFADNCNKWRAYFPAQDPFPKIDSGIK
>KAJ9142978.1:15-591 Laccase [Pleurostoma richardsiae]
LLAGLSRAVPSPPAPRAPTCNTPSNRACWTSGFDINTDYESSTPVTGKTRPYVLTLTEADNLTGPD
GVTKDKVMLVNGDI
LGPTIVADWGDRISITVINNLKTNGTSIHWHGIRQLGTNLQDGTNGVTECPIPPRGGSKVYNFLAT
QYGTSWYHSHFSAQ
YGNGIAGPIQINGPASLPYDIDLGTFPITDYYYKTADELVEITQTQGPPPSDNVLFNGTNIHPITGTG
KYSNVTLTPGKR
HRLRLINTSVENHFQVSLVNHTMTVIAADFVPVNAYTVDSLFLGIGQRYDVTLDASKPIDKYWFN
VTFGGQNFCGSSNNP
KPAAIFHYVGASGGRPTSPGVDPVDAQCNDLINLTPVITRAVATSRFIPDAGNTLPVTLDGPPTSPL
FVWKVNGASINVD
WTKPVVEYILEGNTSYPASENIVSVDQENQWVYWLISNDPDAAFSLPHPIHLHGHDFVVLGRSPD
ATPASQTRYVFDAAT
DISRLNGNNPTRRDVTMLPAKGWVLVAFKTDNPGAWLLHCHIAWHASAGLGVDFLERANDFK
GKISAEDAAAFSQNCASW
RAYFPSMDPFPKIDSGI
>KAK0648653.1:3-623 laccase [Cercophora newfieldiana]
FLTGALAVVGLLASGVASLPQIPEPNPIPKKYPGTSADLEIRGQEAELEFRSELEPRVPPGSCHSASN
RACWTTGFNINT
DYEVSTPPSGAIRKYTLTLTEVDNWTGPDGVVKKKVMLVNGKILGPTILADWGDFIEVKVINNLL
SNGTSIHWHGLHQKG
TQLHDGANGVTECPIPPNGGTFTYKFRATQYGSSWYHSHFSAQYGNGVVGTIQINGPASLPYDID
LGVFPVTDYYYATAD
ELVLFTKNNGPPFSDNVLFNGTNKHPVTGAGNYAEVKLTPGKRHRLRLINTSTENHFQLSLVGH
QMTIIAADFVPVNAMT
VQSVFLAVGQRYDVTIDASATPGNYWFNATFGGQDFCGNSNNPAPAAIFRYTNSPNPTALPTNP
GTRPTDSQCNDILNLT
PVVQRTVPAVQNFVKRPANTLPVQLQIGGTQLFTWKINGSAIDVNWSKPVAQYVIEGNTSYPTQD
NIVHVDEVDQWTYWL
IENDPDNVVSLPHPFHLHGHDFVVLGRSPDATPASQTRYVFDAAVDKFRLTGANPVRRDVAMLP
ARGWLLIAFKTNNPGA
WLMHCHIAWHVSHGLSVDFLERANDFRNGLSAADKAGFNDNCNAWRTWWATAPFPKEDSGL
>QDB06356.1:1-590 laccase, partial [synthetic construct]
APSTHPSSNPDILLERDDHSLTSRQGSCHSPSNRACWCSGFDINTDYETKTPNTGVVRRYTFDITEV
DNRPGPDGVIKEK
LMLINDKLLGPTIFANWGDTIEVTVNNHLRTNGTSIHWHGLHQKGTNYHDGANGVTECPIPPG
GSRVYSFRARQYGTSWY

HSHFSAQYGNGVSGAIQINGPASLPYDIDLGVLPLQDWYYKSADQLVIETLAKGNAPFSDNVLIN
GTAKHPITGEGEYAV
VKLTPGKRHRLRLINMSVENHFQVSLAKHTMTVIAADMVPVNAMTVDSLFMAVGQRYDVTIDA
SQAVGNYWFNITFGGQQ
KCGFSHNPAPAAIFRYEGAPDALPTDPGAAPKDHQCLDTLDLSPVVQKNVPVDGFVKEPGNTLP
VTLHVDQAAAPHVFTW
KINGSAADVDWDRPVLEYVMNNDLSSIPAKNNIVRVDGVNEWTYWLVENDPEGRLSLPHPMHL
HGHDFFVLGRSPDVSPD
SETRFVFDPAVDLPRLRGHNPVRRDVTMLPARGWLLLAFRTDNPGAWLFHCHIAWHVSGGFSV
DFLERPDELRGQLTGES
KAELERVCREWTDWEAKSPHGKIDSGLKQR
>KAK4446538.1:3-616 laccase-1 [Podospora aff. communis PSN243]
FLTGALALVGLLAPAVLALPQIPEPNPIPKDYGVNSAELEIRTDPDLEPRAPPTCHSPSNRACWTTG
FNINTDYEVHTPP
AGATRKYTLTLTEVDNWLGPDGVVKKKVMLVNGSILGPTIRADWGDWIEVKVINNLRTNGTSIH
WHGLHQKGTQLHDGAN
GVTECPIPPLGGQFTYRFRATQYGTSWYHSHFSAQYGNGVVGTIQIDGPASLPYDIDLGVFPITDYY
YAAADELVIFTKD
NGPPFSDNVLFNGTNVHPVTGAGKYAEVKLTPGKRHRLRLINPSTENHFQLSLVGHQMTIIAADF
VPVNAMTVQSVFLGV
GQRYDVTIDASATPGNYWFNATFGGQDFCGNSHNPAPAAIFRYTNSPNPTALPTNPGTPPVDSQ
CNDILNLSPVVQRNVP
NVQSFVKRPANTLDVELQIGGATQLFTWKINGSAIDVDWSKPVAQYVIEGNTSYPTQDNIVHVNE
VDQWTYWLIENDPDN
VVSLPHPFHLHGHDFVVLGRSPDATPASQTRYVFEEAIDKWRLSGANPVRRDVTMLPAKGWVLI
AFKTNNPGAWLMHCHI
AWHVSHGLSVDFLERVQDFRNGLSASDKAGFTNNCNNWRAWWANAPFPKLDSGL
>OIW22748.1:1-595 laccase [Coniochaeta ligniaria NRRL 30616]
MKFFSASIVTLFTLLSSTVIEAAPPSFPALEARASTCNTASNRACWTTGFDINTDYETSTPTTGVTRT
YSLSITEVDNWT
GPDGVVKPKVMLVNGKLQGPTIFADWGDRINITVTNNLRTNGTSIHWHGLRQFGSNLQDGANG
VTECPIPPKGGSKVYSF
VATQYGTSWYHSHFSAQYGNGVVGTIQINGPASLPYDIDLGVFPITDYYYQTADDLVEFTKNNGA
PASDNILINGTNVHP
VTGAGKYANVTLTPGKRHRLRLINTSVENHFQVSLVNHSMTVIAADMVPVNSYTTDTLFLGVGQ
RYDVTIDASKAVGNYW
FNVTLPPNGFCGTSAHPAPAAIFHYAGASGGLPTAPGTPPVDTLCYDNTDLVPVVSRTASPGNFN
PSPNNSLPVHLDTSG
SPLFVWQMNGSSINVNWNKPVLQYVIEGNTSYPPSENIVSVASNNQWSYWLIENDIDAVFSLPHP
FHLHGHDFLVLGRSP
VATPAFPQTRYKFNPATDLSLLKGNNPTRRDVTMLPAKGWLLIAFKTDNPGAWLMHCHIAWHV
SGGLSVDFLEGVSTLRN
NLPAADKAAFADNCNKWNAYFPSQDPFPKVDSGIK
>KAK3391351.1:2-606 Cupredoxin [Sordaria brevicollis]
KFIGIAALVAGLLAPSLVLGVPAPGTERNLLTPVYKRQDFVAEGFGGGGGGSCNSPNNRQCWTRG
FDINTDYELGTPNTG
NTRRYTLTLTETDNWTGPDGVRKDKVMMVNNKIIGPTLQADWGDYLEITVINRLKSNGTSIHWH
GMHQRNTNIHDGANGV
TECPIPPNGGRKVYRFRATQYGTSWYHSHFSAQYGNGIVGSLVINGPASANYDVDLGPFPLMDYY
YETADRIAHKTQSQG
PPFSDNVLFNGFAKHPTTGAGQYATVSLTKGKKHRLRLINTSVENHFQLSLVNHSMTIIAADLVPV
QPKKVDSLMLGVGQ

RYDVIIDANQAVGNYWFNVTFGGANLCGNSNNPAPAAIFRYQGAPNTLPTNKGVAPPDHQCLD
LVDLTPVLQRSMNTNSI
ALNTGNKIPITLDGFTWRVNGSAINVNWNKPVLEYVMTGNTSYSESDNIVHIDGVNQWKYWLIE
NDPDGGFSLPHPIHLH
GHDFLILGRSPDVAAISQTRYVFDPAVDIPRLKGNNPTRRDVAMLPAKGWLLIAFRTDNPGAWLM
HCHIAWHVSGGLSNQ
FLERAQDLKNGISPADKKAFNDNCNAWRAYFPDNAPFPKDDSGLR
>KAB5559524.1:1-590 benzenediol:oxygen oxidoreductase [Coniochaeta sp. 2T2.1]
MKFFSASVVTLFTFLSSAVVEAAPPSFKELDLRASTCNTPSNRACWTSGFNINTDYDANTPTTGVT
RTYALTITEVDNWT
GPDGVVKPKVMLVNGPTIFADWGDRINITVTNNLRTNGTSIHWHGLRQLGSNLHDGANGVTEC
PIPPKGGSKVYSFIAAQ
YGTSWYHSHFSAQYANGVVGSIQIEGPASLPYDIDLGVFPITDYYYRTADDLVEFTKNNGAPPSDN
VLINGTNVHPVTGA
GKYANVTLTPGKRHRLRLINPSVENHFQVSLVNHSMTVIAADLVPVNAYTTNTLFLGVGQRYDV
TIDASKPIGNYWFNVT
LPPNGFCGTSNHPAPAAIFHYAGAPGGLPTNPGTPPVDTLCYDNTDLVPVVSRTAAPGNFNPNP
NNSLPVHLDTTGSPLF
VWQINGSSINVNWNKPVLQYVIEGNTSYPPSENIISVPSNNQYSYWLIENDLDAAFSLPHPFHLHG
HDFLILGRSPVASP
VPPQTRYKFNPATDLSLLRGSNPTRRDVTMLPAKGWLLIAFRTDNPGAWLMHCHIAWHVSGGLS
VDFLEGVSTLRNNLPA
ADKAAFADNCNKWRAYFPSMDPFPKSDSGI
>KAJ4407264.1:2-608 laccase, multicopper oxidase, benzenediol:oxygen oxidorectuctase [Neurospora sp. IMI 360204]
KFFGIAALVAGLLAPSLVLGAPAPGNEGRNLLTPVDKRQDFQAEGYGGGGGGGCNSPSNRQCWS
QGFNINTDYELGTPNT
GNTRRYTLDLTETDNWTGPDGVVKEKVMMVNGKIIGPTLQANWGDYLEITVINRLKTNGTSIH
WHGMHQRNSNIQDGVNG
VTECPIPPNGGSKVYRFRATQYGTSWYHSHFSAQYGNGIVGSIVINGPASANYDVDLGPFPLMDY
YYKTADQIALLTQQA
GPPPSDNVLFNGFAKHPTTGAGQYATVSLTKGKKHRLRLINTSVENHFQLSLVNHSMTIISADLVP
VQPYKVDSLLLGVG
QRYDVIIDANQAVGNYWFNVTFGGSGFCGTSQNPSPAAIFRYQGAPNALPTNQGIAPLDHQCLD
LNDLKPVLQRSLNTNS
IALNTGNTIPIKLDGFVWRVNGTAINVDWNKPVLEYVLTGNTSYPQSENIVQIDGVNQWKYWLIE
NDPDPRTVSLPHPIH
LHGHDFLILGRSPDVDPASQTRYVFDPAVDMPRLKGNNPTRRDVAMLPARGWLLIAFRTDNPGA
WLMHCHIAWHVSGGLS
NQFLERAQDLKNSISDADKNAFNDNCNAWRGYFPANDPFPKPDSGLR
>KAK0750508.1:17-609 Melanocarpus Albomyces laccase [Schizothecium vesticola]
PSLVAAAPSADRDVDLEIRGDVEARAPPTCHSASNRACWTTGFNINTDYEVTAPPPGATRKYTLN
ITEEENWTGPDGVVK
VKVMLVNGPTILADWGDWIEVTVNNKLDTNGTSIHWHGLHQKGTQLHDGANGVTECPIPPGH
SFKYRFRAEQYGSSWYHS
HFSAQYGNGVVGTIQINGPASLPYEVDLGVFPISDYYYKPADQIVIDTRTQGPPPADNVFFNGTNV
HPTSGGGQYSVTTL
QPGKRHRLRIINTSTENHFQLSLVGHQMTIIAADFVPVNAMTVSSVFLAVGQRFDVTIDASQTPG
NYWFNATFGGQNFCG
VTVANNPTPAAIFRYAGAPATLPTNPGVRPTDFQCSDILNLSPVVQRTVPITAFTPRPANTLKVTLD
GPPTSPLFVWKIN

GSAINVNWEKPVAQYVMESNTSYPTSDNIVHVNDVNAWTYWLVENDPDGFSLPHPFHLHGHDF
VVLGRSPDATPASQTRY
VFNQATDSSRLSGANPVRRDVTMLPAKGWIVIAFKTNNPGAWLMHCHIAWHVSHGLSVDFLER
VDDFRASISTVDRNAFN
DNCNAWRTYWPTNPFPKLDSGLKMRALDDLKML
>KAH8902917.1:1-602 laccase [Coniochaeta sp. PMI_546]
MKFFSASIVTLFTYLSSTVVEAAPPSIRELEARAPTCNTPSNRACWTTGFDINTDYETSTPTTGVTRT
YSLSITEVDNWT
GPDGVVKPKVMLINGQGCLLNTIFIGPTIFADWGDRINITVTNNLRTNGTSIHWHGLRQFGSNLQ
DGANGVTECPIPPKG
GSKVYSFVATQYGTSWYHSHFSAQYGNGVVGTIQINGPASLPYDIDLGVFPITDYYYRTADDLVEF
TKNNGPPSSDNILI
NGTNVHPVTGAGKYANVTLTPGKRHRLRLINTSVENHFQVSLVNHSMTVIAADLVPVNAYTTNT
LFLAVGQRYDVTIDAS
KPIGNYWFNVTLPPNGFCGTSVHPAPAAIFHYAGASGGLPTVPGTPPVDTLCYDNVDLVPVVSRT
ATPGNFNPSPNNSLP
VHLDSSGSPLFVWQINGSSINVDWNKPVLQYVIEGNTSYPPSENIVSVASTNQWSYWLIENDVDA
VFSLPHPFHLHGHDF
LILGRSPVASPAFPQTRYKFDPATDLSLLKGNNPTRRDVAMLPAKGWLLIAFKTDNPGAWLMHC
HIAWHVSGGLSVDFLE
GVSTLKNTLPAADKAAFADNCNKWRAYFPSQDPFPKIDSGIK
>XP_956939.1:2-607 laccase [Neurospora crassa OR74A]
KFLGIAALVAGLLAPSLVLGAPAPGTEGVNLLTPVDKRQDSQAERYGGGGGGGCNSPTNRQCWS
PGFNINTDYELGTPNT
GKTRRYKLTLTETDNWIGPDGVIKDKVMMVNDKIIGPTIQADWGDYIEITVINKLKSNGTSIHWH
GMHQRNSNIQDGVNG
VTECPIPPRGGSKVYRWRATQYGTSWYHSHFSAQYGNGIVGPIVINGPASANYDVDLGPFPLTDY
YYDTADRLVLLTQHA
GPPPSNNVLFNGFAKHPTTGAGQYATVSLTKGKKHRLRLINTSVENHFQLSLVNHSMTIISADLVP
VQPYKVDSLFLGVG
QRYDVIIDANQAVGNYWFNVTFGGSKLCGDSDNHYPAAIFRYQGAPKALPTNQGVAPVDHQCL
DLNDLKPVLQRSLNTNS
IALNTGNTIPITLDGFVWRVNGTAININWNKPVLEYVLTGNTNYSQSDNIVQVEGVNQWKYWLI
ENDPDGAFSLPHPIHL
HGHDFLILGRSPDVTAISQTRYVFDPAVDMARLNGNNPTRRDTAMLPAKGWLLIAFRTDNPGSW
LMHCHIAWHVSGGLSN
QFLERAQDLRNSISPADKKAFNDNCDAWRAYFPDNAPFPKDDSGLR
>KAK0612088.1:3-615 laccase [Immersiella caudata]
FLAGALALVGLLAPTVLALPQIPEPNPIPKDYNVNSAELEIRTDPDLEPRAPPTCHSPSNRACWTT
GFNINTDYEVLTPP
AGTTKKYTLTLTEVDNWLGPDGVVKKKVMLVNGSIFGPTIRADWGDWIEVKVINNLRTNGTSIH
WHGLHQKGTQLHDGAN
GVTECPIPPNGGQFTYRFRATQYGSSWYHSHFSAQYGNGVVGTIQIDGPASLPYDIDLGVFPITDY
YYAAADELVIFTAN
NGPPFSDNVLFNGTNVHPTTGAGNYAEVKLTPGKRHRLRLINTSTENHFQLSLVGHQMTIIAADF
VPVNAMTVQSVFLGV
GQRYDVTIDASATPGNYWFNATFGGQDFCGNSHNPAPAAIFRYTNSPNPTALPTNPGTPPVDSQ
CNDILNLTPVVQRTVP
NVQNFVKRPANTLDVELQIGGTQLFTWKINGSAINVDWSKPVAQYVIEGNTSYPTQDNIVHVNE
VDQWTYWLIENDPDNV
VSLPHPFHLHGHDFVVLGRSPDATPASQTRYVFQEAIDKWRLNGANPIRRDVAMLPAKGWLLIA
FKTNNPGAWLMHCHIA

WHVSHGLSVDFLERVQDFRNGLSASDKAGFTNNCNAWRTWWATAPFPKIDSGL
>XP_024511624.1:2-606 uncharacterized protein SMAC_06098 [Sordaria macrospora k-hell]
KFFGIAALVAGLLAPSLVLGAPALGNDRDLLTPVDKRQDFVAPGFGGGFGGNCNSPTNRQCWSL
GFNINTDFELGTPTTG
NTRRYTLTLTETDNWIGPDGVKKDKVMMVNNKIIGPTLQANWGDYLEITVINRLKSNGTSIHWH
GMHQRNTNIHDGVNGV
TECPIPPNGGSKVYRFRATQYGTSWYHSHFSAQYGNGIVGSIVINGPASANYDVDLGPFSLMDYY
YETADRIMLLTQRAG
PPPSDNVLFNGFAKHPTTGAGQYATVSLTKGKKHRLRLINTSVENHFQLSLVNHSMTIIAADLVPV
QPYKVDSLLLGVGQ
RYDVIIDANQAIGNYWFNVTFGGSGLCGLSNNPAPAAIFKYQGAPNGLPTNKGVAPPDHQCLDL
VDLKPVLQRSLNTNSI
ALNTGNTIPITLDGFVWRVNGTAINVDWNKPVLEYVMTGLTNYSESDNIVKVDGVNQWKYWLI
ENDPDGPFSLPHPIHLH
GHDFLILGRSPDVAAISQTRYVFDPAVDIPRLKGNNPTRRDVAMLPAKGWLLIAFRTDNPGAWLM
HCHIAWHVSGGLSNQ
FLERAQDLKNGISNADKQAFNNNCNAWRAYFPDNAPFPKDDSGLR
>KHE82513.1:2-607 multicopper oxidase [Neurospora crassa]
KFLGIAALVAGLLAPSLVLGAPAPGTEGVNLLTPVDKRQDSQAERYGGGGGGGCNSPTNRQCWS
PGFNINTDYELGTPNT
GKTRRYKLTLTETDNWIGPDGVIKDKVMMVNDKIIGPTIQADWGDYIEITVINKLKSNGTSIHWH
GMHQRNSNIQDGVNG
VTECPIPPRGGSKVYRWRATQYGTSWYHSHFSAQYGNGIVGPIVINGPASANYDVDLGPFPLTDY
YYDTADRLVLLTQHA
GPPPSNNVLFNGFAKHPTTGAGQYATVSLTKGKKHRLRLINTSVENHFQLLLVNHSMTIISADLVP
VQPYKVDSLFLGVG
QRYDVIIDANQAVGNYWFNVTFGGSKLCGDSDNHYPAAIFRYQGAPKALPTNQGVAPVDHQCL
DLNDLKPVLQRSLNTNS
IALNTGNTIPITLDGFVWRVNGTAININWNKPVLEYVLTGNTNYSQSDNIVQVEGVNQWKYWLI
ENDPDGAFSLPHPIHL
HGHDFLILGRSPDVTAISQTRYVFDPAVDMARLNGNNPTRRDTAMLPAKGWLLIAFRTDNPGSW
LMHCHIAWHVSGGLSN
QFLERAQDLRNSISPADKKAFNDNCDAWRAYFPDNAPFPKDDSGLR
>AAA33591.1:2-607 laccase [Neurospora crassa]
KFLGIAALVAGLLAPPLVLAAPAPGTEGVNLLTPVDKRQDSQAERYGGGGGGGCNSPTNRQCWS
PGFNINTDYELGTPNT
GKTRRYKLTLTETDNWIGPDGVIKDKVMMVNDKIIGPTIQADWGDYIEITVINKLKSNGTSIHWH
GMHQRNSNIQDGVNG
VTECPIPPRGGSKVYRWRATQYGTSWYHSHFSAQYGNGIVGPIVINGPASANYDVDLGPFPLTDY
YYDTADRLVLLTQHA
GPPPSNNVLFNGFAKHPTTGAGQYATVSLTKGKKHRLRLINTSVENHFQLLLVNHSMTIISADLVP
VQPYKVDSLFLGVG
QRYDVIIDANQAVGNYWFNVTFGGSKLCGDSDNHYPAAIFRYQGAPKALPTNQGVAPVDHQCL
DLNDLKPVLQRSLNTNS
IALNTGNTIPITLDGFVWRVNGTAININWNKPVLEYVLTGNTNYSQSDNIVQVEGVNQWKYWLI
ENDPDGAFSLPHPIHL
HGHDFLILGRSPDVTAISQTRYVFDPAVDMARLNGNNPTRRDTAMLPAKGWLLIAFRTDNPGSW
LMHCHIAWHVSGGLSN
QFLERAQDLRNSISPADKKAFNDNCDAWRAYFPDNAPFPKDDSGLR
>KAK1830439.1:1-605 Cupredoxin [Schizothecium conicum]
MRFLSGAVALAVGLLAPSLVAAAPTADREIDLEIRGDVEPRAPPTCHSASNRACWTTGFNINTDYE
LTAPPPGTTRKYTL

NLTEEEDWTGPDGVVKSKVMLVNGKILGPTIVADWGDWIEVTVNNKLDTNGTSIHWHGLHQK
GTQLHDGANGVTECPIPP
NHSFKYRFRAEQYGSSWYHSHFSAQYGNGVVGTILIHGPASLPYEVDLGVFPITDYYYKPADQIVI
DTMTQGPPPADNVL
FNGTNIHPTSGGGAYSITKLQPGKRHRLRIINTSTENHFQLSLVGHQMTIIAADFVPVNAMTVDSV
FLAVGQRYDVTIDA
SQTPGNYWFNATFGGQTFCGTTVANNPTPAAIFRYEGAPATLPTNPGVRPADAQCNDILNLTPVV
QRTVPITAFTPRPAN
TLHVSLDGPPASPLFVWKINGSAINVNWEKPVAQYVMEGNTSYPTSDNIVHVNEVNAWTYWLV
ENDPDGFSLPHPFHLHG
HDFVVLGRSPDATPASQTRYVFDQATDRHRLNGQNPVRRDVTMLPAKGWIVIAFKTNNPGAWL
MHCHIAWHVSHGLSVDF
LERADDFRAQISTADRNAFNDNCDAWRTYWPTNPFPKLDSGLKVR
>ROW00953.1:33-590 hypothetical protein VSDG_02891 [Valsa sordida]
VLARQVESCNTASNRACWTDGFDINTDYEVDTPITGIVRNYTLTLTEEDNWTGPDGVVKEKVML
VNGDIHGPTIYAQWGD
TITVRVINNLETNGTSVHWHGIRQFGSNLQDGVNGITECPLPPGSERTYTYIARQYGTSWYHSHFS
AQYGNGVVGAIHID
GPASLPYDIDLGPFILSDYYHKTADELVIYTETNGAPASDNVLFNGTTVDPDTSAGEYAKITLTPGK
RHRLRLINTSVEN
HFQVSIVDHSMTIISSDFVPVNSFTTDSIFIGVGQRYDVTIDASQDVANYWFNVTYGGGGLCGSSN
NPYPAAIVQYDGAP
DGNPTDEGTAPIDHMCLDTMDISPVVSRTVPTAFTASSGNTMDVHLDLTAPTFVWYINNSSEKVD
WDKPVAQYVAENETS
FPSDLNTWTVDEEDQWVYWLIENDPNALFSLPHPIHLHGHDFVVLGRSPDTSPITQTTYTFDASL
LSGSNPVRRDVTMLP
AAGWLVLAFKTDNPGAWLMHCHIAWHVAGGLSVTFLERLSDYRAGMNQTDIDVLDSQCTDW
NNYFPSQDPYPQLDSGI
>AAA33592.1:2-607 laccase [Neurospora crassa]
KFLGIAALVAGLLAPSLVLGAPAPGTEGVNLLTPVDKRQDSQAERYGGGGGGGCNSPTNRQCWS
PGFNINTDYELGTPNT
GKTRRYKLTLTETDNWLGPDGVIKDKVMMVNDNIIGPTIQADWGDYIEITVINKLKSNGTSIHWH
GMHQRNSNIQDGVNG
VTECPIPPRGGSKVYRWRATQYGTSWYHSHFSAQYGNGIVGPIVINGPASANYDVDLGPFPLTDY
YYDTADRLVLLTQHA
GPPPSNNVLFNGFAKHPTTGAGQYATVSLTKGKKHRLRLINTSVENHFQLSLVNHSMTIISADLVP
VQPYKVDSLLLGIG
QRYDVIIDANQAVGNYWFNVTFGGNDLCGTSDNKYPAAIFRYQGAPKALPTNKGVAPPDHQCL
DLNDLKPVLQRSLNTNS
IALNTGNTIPITLDGFVWRVNGTAININWNKPVLEYVMTGNTNYSQSDNIVQVEGVNQWKYWLI
ENDPDGAFSLPHPIHL
HGHDFLILGRSPDVTAISQTRYVFDPAVDMARLNGNNPTRRDTAMLPAKGWLLIAFRTDNPGSW
LMHCHIAWHVSGGLSN
QFLERAQDLRNSISPADKKAFNDNCDAWRAYFPDNAPFPKDDSGLR
>KAK3952309.1:20-615 Cupredoxin [Pseudoneurospora amorphoporcata]
VLGAPAPDNENRNLLTPVDKRQDFTTEGYGDGGGYGGGGGGGGCNSPKNRQCWSGGFNINTD
YELGTPNTGNTRRYTLTL
TETDNWIGPDGVKKDKVMMVNGKIIGPTLQADWGDYFEITVINRLKSNGTSIHWHGMHQRNS
NIQDGANGVTECPIPPNG
GSKVYRFQATQYGTSWYHSHFSAQYGNGIVGAIVINGPASANYDVDLGAFPLMDYYYETADRIV
LLTQHAGPPPSDNVLF

NGFAKHPTTGAGQYATVSLTKGKKHRLRLINTSVENHFQLSLVNHSMTIIAADLVPVQPYKVDSL
LLGVGQRYDVIIDAN
QAVGNYWFNVTFGGNGLCGACNNPKPAAIFKYQGAPNGLPTNQGVVPPDHQCLDLVDLKPVL
QRSLNTDSIALNPGNTIP
ITLDGFVWRVNGTAMNVDWNKPVLEYVLTGNTNYSQSDNIVQIDGVNQWKYWLIENDPDGGF
SLPHPIHLHGHDFLVLGR
SPDVAAISQTRYVFDPAVDMPRLKGNNPTRRDVAMLPAKGWLLIAFRTDNPGAWLMHCHIAWH
VSGGLANQFLERAQDLK
NGISNADKKAFNDNCNAWRAYFPDNAPYPKDDSGLR
>XP_009853179.1:1-607 uncharacterized protein NEUTE1DRAFT_85559 [Neurospora tetrasperma FGSC 2508]
MKHFGIAALVAGLLAPSLILGAPAPGNEGVNLLTPVNKRQDFQAEGYGGGGGGGCNSPTNRQC
WSPGFNINTDYELGTPN
TGKTRRYKLTLTETDNWTGPDGVKKDKVMMVNGKIIGPTLQADWGDYLEITVINKLKSNGTSIH
WHGMHQRNSNIQDGVN
GVTECPIPPNGGSKVYRWRATQYGTSWYHSHFSAQYGNGVVGPIVINGPASANYDVDLGPFPLT
DYYYDTADRLVLLTQR
AGPPPSNNVLFNGFAKHPTTGAGQYATVSLTKGKKHRLRLINTSVENHFQLSLVNHSMTIISADLV
PVQPYKVDSLLLGV
GQRYDVIIDANQAVGNYWFNVTFGGSKLCGDSDNKYPAAIFRYQGAPKALPTNKGVAPPDHQC
LDLNDLKPVLQRSLNTN
SIALNTGNTIPITLDGFVWRVNGTAININWNKPVLEYVLTGNTNYSQSDNIVQVDGVNQWKYWL
IENDPDGAFSLPHPIH
LHGHDFLILGRSPDVTAISQTRYVFDPAVDMARLNGNNPTRRDTAMLPAKGWLLIAFRTDNPGS
WLMHCHIAWHVSGGLS
NQFLERAQDLRNSISPADKKAFNDNCEAWRAYFPDNAPFPKDDSGLR
>KAK1776634.1:1-609 multicopper oxidase-domain-containing protein [Copromyces sp. CBS 386.78]
MKFFGIAAALVAGLLAPSLVLGAPAPGNENRNLLTPVDKRQDFTTEGFGGGGGGGCNSPNNR
QCWSGGFNINTDYELGT
PNTGNTRRYTLTLTEADNWIGPDGVKKDKVMMVNGKIIGPTLQADWGDYFEITVINRLQSNGTS
IHWHGMHQRNSNIQDG
ANGVTECPIPPNGGSKVYRFRATQYGTSWYHSHFSAQYGNGIVGAVVINGPASANYDVDLGPFP
LMDYYYETTDRIVLLT
QHAGPPPSDNVLFNGFAKHPTTGAGQYATVSLTKGKKHRLRLINTSVENHFQLSLVNHSMTIIAA
DLVPVQPYKVDSLLL
GVGQRYDVIIDANQAVGNYWFNVTFGGNGLCGACNNPKPAAIFKYHGAPNGLPTNQGVNPPD
HQCLDLVDLKPVLQRSLN
TDSIALNPGNTIPITLDGFVWRVNGTAINVDWNKPVLEYVLTGNTNYSQSDNIVQIDGVNQWKY
WLIENDPDGGFSLPHP
IHLHGHDFLVLGRSPDVAAISQTRYVFDPAVDMPRLKGNNPTRRDVAMLPAKGWLLIAFRTDNP
GAWLMHCHIAWHLSGG
LSNQFLERAQDLKNGISDADKKAFNDNCNAWRAYYPDNAPYPKDDSGLR
>AIW52362.1:2-607 laccase, partial [Sordaria fimicola]
KFFGIAALVAGLLAPSLVLGAPALGNDRVNLLTPVDKRQDFVAPGFGGGFGGNCNSPTNRQCWS
LGFNINTDFELGTPTT
GNTRRYTLTLTETDNWIGPDGVKKDKVMMVNNKIIGPTLQANWGDYLEITVINRLKANGTSIH
WHGMHQRNTNLHDGVNG
VTECPIPPNGGSKVYRFRATQYGTSWYHSHFSAQYGNGIVGSIVVNGPASANYDVDLGPFPLMDY
YYETADRLVLLTQRA
GPPPSDNVLFNGFAKHPTTGAGQYATVSLTKGKKHRLRLINTSVENHFQLSLVNHSMTIIAADLVP
VQPYKVDSLLLGVG

QRYDVIIDANQAIGNYWFNVTFGGSGLCGLSNNPAPAAIFKYQGAPNGLPTNKGVAPPDHQCL
DLVDLKPVLQRSLNTNS
IALNTGNTIPITLDGFVWRVNGTAIDVNWNKPVLEYVMTGLTNYSESDNIVKVDGVNQWKYWLI
ENDPDGPFSLPHPIHL
HGHDFLILGRSPDVPAISQTRYVFNPAVDMPRLKGNNPTRRDVAMLPAKGWLLIAFRTDNPGAW
LMHCHIAWHVSGGLSN
QFLERAQDLKNGISNADKQAFNNNCNAWRAYFPDNAPFPKDDSGLR
>KAK3485539.1:2-607 Cupredoxin [Neurospora hispaniola]
KFFGIAALVAGLLAPSLVLSAPAPGNEGVALLTPVDKRQDFQAEGYGGGGGGGCNSPTNRQCWS
SGFSINTDYELGTPNT
GKTRRYKLTLTETDNWTGPDGVKKDKVMMVNNKIIGPTLEADWGDYLEITVINKLKSNGTSIHW
HGMHQRNSNIQDGVNG
VTECPIPPNGGSKVYRWRATQYGTSWYHSHFSAQYGNGIVGPIVINGPASANYDVDLGPFPLTDY
YYDTADRLVLLTQRA
GPPPSNNVLFNGFAKHPTTGAGQYATVSLTKGKKHRLRLINTSVENHFQLSLVNHSMTIISADLVP
VQPYKVDSLLLGIG
QRYDVIIDANQAVGNYWFNVTFGGSGLCGTSDNKYPAAIFRYQGAPKALPTNKGVAPPDHQCL
DLNDLKPVLQRSLNTNS
IALNTGNTIPITLDGFVWRVNGTAININWNKPVLEYVMTGNTNYSQSDNIVQVDGVNQWKYWL
IENDPDGAFSLPHPIHL
HGHDFLILGRSPDVTAISQTRYVFDPAVDMARLKGNNPTRRDTAMLPAKGWLLIAFRTDNPGSW
LMHCHIAWHVSGGLSN
QFLERAQDLRNSISPADKKAFNDNCDAWRAYFPDNAPFPKDDSGLR
>KAK3496331.1:2-607 laccase [Neurospora crassa]
KFLGIAALVAGLLAPSLVLGAPAPGTEGVNLLTPADKRQDSQAERYGGGGGGGCNSPTNRQCWS
PGFNINTDYELGTPNT
GKTRRYKLTLTETDNWTGPDGVKKDKVMMVNGKIIGPTLEADWGDYLEITVINKLKLNGTSIHW
HGMHQRNSNIQDGVNG
VTECPIPPNGGSKVYRWRATQYGTSWYHSHFSAQYGNGVVGPIVINGPASANYDVDLGPFPLTD
YYYDTADRLVLLTQHA
GPPPSNNVLFNGFAKHPTTGAGQYATVSLTKGKKHRLRLINTSVENHFQLSLVNHSMTIISADLVP
VQPYKVDSLLLGIG
QRYDVIIDANQAVGNYWFNVTFGGSGLCGTSDNKYPAAIFRYQGAPKALPTNQGVAPPDHQCL
DLNDLKPVLQRSLNTNS
IALNTGNTIPITLDGFVWRVNGTAININWNKPVLEYVLTGNTNYSQSDNIVQVDGVNQWKYWLI
ENDPDGAFSLPHPIHL
HGHDFLILGRSPDVTAISQTRYVFDPAVDMARLNGNNPTRRDTAMLPAKGWLLIAFRTDNPGSW
LMHCHIAWHVSGGLSN
QFLERAQDLRNSISAADKKAFNDNCDAWRAYFPDNAPFPKDDSGLR
>KAK3347833.1:2-612 multicopper oxidase-domain-containing protein [Neurospora tetraspora]
KFFGIAAIVAGLLAPSLVLGAPAPGNEGRNLLTPVDKRQDFQAEGYGGGGGGGCNSPSNRQCWS
QGYNINTDYELGTPNT
GNTRRYTLDLTETDNWIGPDGVKKDKVMMVNGKIIGPTLQADWGDYLEITVINRLKTNGTSIHW
HGMHQRNSNIQDGVNG
VTECPIPPNGGSKVYRFRATQYGTSWYHSHFSAQYGNGIVGSIVINGPASANYDIDLGPFPLMDYY
YETADRIALLTQHA
GPPPSDNVLFNGFAKHPTTGAGQYATVSLTPGKKHRLRLINTSVENHFQLSLVNHSMTIISADLVP
VIPRKVDSLLLGVG
QRYDVIIDANQNPKQAGYGNYWFNVTFGGSGFCGTSHNPKPAAIFRYQGVPNALPTIQGVAPPD
HQCLDLNDLKPVVQRS
FNTNSIALNPGNEIPITLDGFVWRVNGTAINVDWNKPVLEYVLTGDTNYPQSENIVKIDGVNQW
KYWLIENDPDGTISLP

HPIHLHGHDFLILGRSPDANVSSQTRYVFDPVVDIPHLNGTNPTRRDVAMLPAKGWLLIAFRTDN
PGAWLMHCHIAWHVS
GGLSNQFLERAQDLKNGISNADKRAFNDNCNAWRGYFPNNDPNPKNDSGLR
>AIW52365.1:2-607 laccase, partial [Sordaria fimicola]
KFFGIAALVAGLLAPSLVLGAPALGNDRVNLLTPVDKRQDFVAPGFGGGFGGNCNSPTNRQCWS
LGFNINTDFELGTPTT
GNTRRYTLTLTETDNWIGPDGVKKDKVRMVNNKIIGPTLQANWGDYLEITVINRLKANGTSIHW
HGMHQRNTNLHDGVNG
VTECPIPPNGGSKVYRFRATQYGTSWYHSHFSAQYGNGIVGSIVVNGPASANYDVDLGPFPLMDY
YYETADRLVLLTQRA
GPPPSDNVLFNGFAKHPTTGAGQYATVSLTKGKKHRLRLINTSVENHFQLSLVNHSMTIIAADLVP
VQPYKVDSLLLGVG
QRYDVIIDANQAIGNYWFNVTFGGSGLCGLSNNPAPAAIFKYQGAPNGLPTNKGVAPPDHQCL
DLVDLKPVLQRSLNTNS
IALNTGNTIPITLDGFVWRVNGTAIDVNWNKPVLEYVMTGLTNYSESDNIVKVDGVNQWKYWLI
ENDPDGPFSLPHPIHL
HGHDFLILGRSPDVPAISQTRYVFNPAVDMPRLKGNNPTRRDVAMLPAKGWLLIAFRTDNPGAW
LMHCHIAWHVSGGLSN
QFLERAQDLKNGISNADKQAFNNNCNAWRAYFPDNAPFPKDDSGLR
>XP_060280685.1:1-588 laccase [Phialemonium atrogriseum]
MRSLFTAFVGLTGLFSSVHGAPSAELVKRAPTCNTPSNRACWTTGFDINTDYLEKTPLTGKTVTYT
LVVTEIDNWKGPDG
VVKKKAMLINGTLPGPTIVADWGDRLNITVINNLRDNGTSFHWHGLRQMGSNFHDGVNGVTE
CPIPPKGGKRLYSFLATQ
YGTGWYHSHFSAQYANGVVGNMVINGPASLPYDIDLGVFPISDYYYETADNLVEFTMNNGPPPS
DNVLFNGTNIHPVTGA
GKYARVKLTPGKRHRLRLINPSVENHFTVSLVDHQMTVIGTDYVPSNAFTTDSLFIGVGQRYDVTI
DASKAVGNYWFNVT
FGGNGFCGLSNNPFPAAIFSYDGAPNANPTKAGTPPADSLCLDNLNLTPVVPRSAPPSGFTPSASN
KLDVHLDTTGTPLF
VWRINGNSIKVDWQKPIGQYVMDGNNSFPANANIVKVDAVNQWTYWLVENDPDGAFSLPHPF
HLHGHDFLVLGRSPDTPP
AAQTRYVFNPATDMGRLNGGNPTRRDVTMLPAKGWVLIAFKTDNPGAWLMHCHIAWHVSGG
LSVDFLERVSEFKAGITAA
DKAIFDNNCNAWRTYAPTNQWSQIDSGI
>KAH8892660.1:10-603 laccase [Thozetella sp. PMI_491]
LSLGTLAPRFAYGAPAPSEELPLLAPIMPRASTCNTPSNRACWTTGFDINTDYETSTPVTNVVRTY
NLEITEHDNWTGPD
GVVRAKAMLINNTFPGPTIVADWGDTIQITVKNSLQTNGTSIHWHGIRQLNTNLADGANGATEC
PIAPGTSKVYTFRALQ
YGSSWYHSHFSAQYANGVVGTIQINGPASLPYDVDLGVFPITDYYYDTADRLVEFTKNNGAPQSD
NVLFNGTNVHPTTGA
GKYANVTLTPGKRHRLRIINTSAENHFQLSLVGHTMTIIGTDFVPVNSYTVNSLFVAIGQRYDVTID
ASQTPGNYWFNVT
FGGSGLCGSSVNPTPAAIFHYAGAAGGLPTNPGVVPPDHQCLDLFNLTPVVQRTVPVSTFSPNITD
TIPIHLDTGGTTPP
TFTWLVNGSAIRVDWNKPVDQYVLEGNTSYPQNENVIQANQVNQWAFWLIENDPDGGFALPH
PIHLHGHDFVLVGRSPDS
GALAQPVFKYNAATDGPGLKSSNPVRRDVTMLPPHGWIVLAFRTDNPGSWLMHCHIAWHISGG
LGVDFVERAVDFKNGVS
ATDKAAFDQNCNSWRAYYPSRDPFQQQDSGLKVR
>KAH8883756.1:1-592 laccase-1 [Thozetella sp. PMI_491]

MFPVVAILVQFLAFIAPGFVQGAPASAELLDISARAPTCNTPSNRACWTTGFSITTDYETSTPFTGV
TRTYNLEITEHDN
WKGPDGVVKEKVMLINSPVITADWGDRITIVVKNSLRANGTSIHWHGIRQLNTNLQDGANGVT
ECPIPPNGGVKTYSFLA
LQYGTSWYHSHFTAQYGNGVVGTIQINGPASLPYDIDLGTFPISDYYYRTADNIVIDTKNNGPPLS
DNVLFNGTNVHPVT
GAGKYANVTLTPGKRHRLRIINTSVENHIQVSLVGHTMTVIAADFVPVNAETVNSLFLGIGQRYD
VTIDASQAVSNYWFN
VTFGGQGFCGGSVNPTPAAIFHYAGASGGNPTNPGTAPPDSNCLDLDNLSPVVTRTVPVANFNP
TPADTLPVHLTSVPGQ
TFLWQVNGSSIVVDWDKPVDEYVLTGNTSYPRSENLIIENSVNQWGFWLISNDPNGAFGLPHPIH
LHGHDFVVVAKSPPV
TPLSQTPFIYNPSTDSGSLRSSNPPRRDVTMLPPKGWLIVAFKTDNPGAWLMHCHIAWHVSGGLS
VDFLERPNDLRSGIS
AADAAAFEANCNAWRSYYPQLDPFKQDDSGLK
>KAB5513366.1:1-584 benzenediol:oxygen oxidoreductase [Coniochaeta sp. 2T2.1]
MKFFSASVVTLFTFWSSAVVEAAPPSFKELDTRASTCNTPSNRACWTNGFNINTDYDTNTPTTGV
TRTYSLTITEVDNWM
GPDGVVKPKVMLVNGKFPGPTIFADWGDRINITVTNNLRTNGTSIHWHGLRQFGSNLQDGANG
VTECPIPPKGGSKVYSF
IAAQYGTSWYHSHFSAQYGNGVVGSIQIEGPASLPYDIDLGVFPITDYYYRTADDLVDDNVLINGT
NVHPVTGAGKYANV
TLTPGKRHRLRLINPSVENHFQVSLVNHSMTVIAADLVPVNAFTTNTLFLGVGQRYDVTIDASKPI
GNYWFNVTLPPNGF
CGTSNHPAPAAIFHYAGAPGGLPTNPGTPPVDTLCYDNTDLVPVVSRTAAPGNFNPNPNNSLPV
HLDTSGSPLFVWQING
SSINVDWNKPVLQYVIEGNTSYPPSENIISVPSNNQYSYWLIENDLDAAFSLPHPFHLHGHDFLILG
RSPVASPVPPQTR
YKFNPATDLALLRGNNPTRRDVTMLPAKGWLLIAFRTDNPGAWLMHCHIAWHVSGGLSVDFLE
GVSTLRSNLPAADKAAF
ADNCNKWRAYFPSMDPFPKSDSGI
>ROV93188.1:24-586 hypothetical protein VMCG_08728 [Valsa malicola]
PSSTNLLEPVLARQVESCNTASNRACWTDGFDINTDYEVDTPITGVLRNYTLTLTEEDNWTGPDG
FVKEKVMLVNGPTIY
GQWGDSISVRVINNLKTNGTSVHWHGMRQMGSNLQDGVNGITECPLAPGSERTYNFIARQYGT
SWYHSHFSAQYGNGVVG
PIQIDGPASLSYDIDLGPFLLTDYYHKTADELVIYTETNGAPPSDNVLFNGTTVDPETGAGEYAKITL
TPGKRHRLRLIN
TSVENHFQVSIVSHSMTIISSDFVPVDSFTTDSLFLGVGQRYDVTIDASQDVGNYWFNVTFGGGGF
CGSSNNSYPAAIVQ
YDGAPDGNPTDEGKAPIDHMCLDTLDIVPVVSRTVPTAFTASSDNTMDVHLDLTAPTFVWYINN
SSEKVDWNKPVAQYVA
NNETDFPSDLNIWKVDDEDQWVYWLIENDPNALFTLPHPIHLHGHDFVVLGRSPDANPVTQTT
YTFDASLVKGNNPVRRD
VTMLPAAGWLLLAFKTDNPGAWLMHCHIAWHVAGGLSVTFLERLSDYRAGMNQTDIDVMNS
QCTDWDNYFPSEDPYPQLD
SGI
>KAH8780311.1:8-585 laccase [Diaporthaceae sp. PMI_573]
FLTGLLSLQSVRAAPSSLLQPIEVRQDAGCNTPSDRACWSDGFDINTDYETTTPLTGVVRPYTLTLT
EETNWKGPDGVIK
DLVMLVNGDIIGPTIHAQWGDTISVRVINNLETNGTSIHWHGFRQVGTNLQDGVNGITECPIPPK
GGERQYTFIARQYGS

AWYHSHFSAQYGNGIIGAVQVDGPASTPYDIDLGPMILTDYYHDSADNIVHYTETNGAPPSDNVL
FNGHTVNPTTGDGEY
EKMTLTPGKRHRLRLINTSVDNHFQVSIVGHDMTVIASDFVPVNAFTTDSLFIGVGQRYDVTIEAK
EDIANYWFNVTFGG
GNLCGASKTPYPAAILHYDGAPDSLPTNEGTRPKDHNCLDLINLTPVVERKVSTAFTPSASNTME
VQLPTGPKFVWKING
QAIRTEWENPVAQYIAANRTDYPETNSIWEVEAVDQWVYWLIENDPEGAFSLPHPIHLHGHDFL
VVGRSPDAAPATQTKY
AFDASMLNGNNPVRRDVAMLPAAGWLLLAFKTDNPGAWLMHCHIAWHISGGLGMTFLERLG
DYQAGLKQSDIDVLNTQCE
AWNEYSNTMPWPQADSGL
>KAK2614816.1:3-586 hypothetical protein N8I77_001615 [Diaporthe amygdali]
LFTSLFFTGLLSLQPARAAPSSLLQAIEVRQDASCNTPSDRACWTDGFDINTDFETKTPLTGVVRPY
TLTLTEETNWKGP
DGVTKDIVMLVNGDIIGPTIYAHWGDTISVKVINNLESNGTSIHWHGVRQVGSNLHDGVNGITEC
PIPPKGGERVYTFIA
RQYGSAWYHSHFSAQYGNGIIGPIQIDGPASLPYDVDLGPMLLTDYYHESADNLVLYTETNGPPPS
DNVLFNGHAVNPST
GDGEYGKLTLTPGKRHRLRLINTSVDNHFQVSIVNHDMTVIASDFVPVNAFTTDSLFIGVGQRYD
VTIEANQDIGNYWLN
VTFGGGNLCGMSNNPYPAAILHYDGAPDGLPTKKGTRPRDHNCLDLLDLTPVVERKISTEFTPSA
DNTMEIQLPAGPKFV
WKINNKSIRTVWQNPVAQYVAANRTDYPETNSIWSVNAVDQWVYWLIENDPEGAFSLPHPIHL
HGHDFLVVGRSPEAAPA
TQTKFAFDASRLNGNNPVRRDVAMLPAAGWLLLAFKTDNPGAWLMHCHIAWHVSGGLGMTF
LERVSDYRAGLKQSDVDVL
NEQCKAWNDYFPHNAPWPQVDSGL
>XP_052995192.1:6-586 uncharacterized protein J7T55_012843 [Diaporthe amygdali]
SLFFTGLLSLQPARAAPSSLLQAIEVRQDASCNTVSDRACWTDGFDINTDFEAKTPLTGVVRPYTL
TLTEETNWKGPDGV
TKDIVMLVNGDIIGPTIYAHWGDTISVKVINNLESNGTSIHWHGVRQVGSNLHDGVNGITECPIPP
KGGERVYTFIARQY
GSAWYHSHFSAQYGNGIIGPIQIDGPASLPYDVDLGPMLLTDYYHESADNLVLYTETNGPPPSDN
VLFNGHAVNPSTGDG
EYGKLTLTPGKRHRLRLINTSVDNHFQVSIVNHDMTVIASDFVPVNAFTTDSLFIGVGQRYDVTIE
ANQDIGNYWLNVTF
GGGNLCGMSNNPYPAAILHYDGAPDGLPTKKGTRPRDHNCLDLLDLTPVVERKVSTEFTPSADN
TMEIQLPAGPKFVWKI
NNKSIRTVWQNPVAQYVAANRTDYPETNSIWSVNAVDQWVYWLIENDPEGAFSLPHPIHLHGH
DFLVVGRSPEAAPATQT
KFAFDASRLNGNNPVRRDVAMLPAAGWLLLAFKTDNPGAWLMHCHIAWHVSGGLGMTFLER
VSDYRAGLKQSDVDVLNEQ
CKAWNDYFPHNAPWPQVDSGL
>KUI54552.1:21-588 Laccase [Valsa mali var. pyri (nom. inval.)]
VPSSANRLEPIFARQQESCNTATDRSCWTDGFDINTDYEISTPLTGVVRNYTLTLTEVDNWTGPDG
YVKEKVMLINDDII
GPTIYAQWGDTISVQVINNLKTNGTSVHWHGIRQLDTNLQDGVNGITECPLPPNSERVYTYIAHQ
YGTSWYHSHFSAQYG
NGVSGPIHIDGPASLPYDIDLGPFVLSDYYHKTADELVIYTKTNGAPPSDNVLFNGTTVDPTTGVG
DYAKITLTPGKRHR
LRLINTSVENHFQLSIVNHNMTIIASDFVPVDAYTTDSLFIGVGQRFDVTIDADQDVDNYWFNVT
YGGGGLCGSSNNPYP

AAIVQYDGAADGNPTDQGTTPKDHYCVDTMDIVPVVSRTVPTSFTASTDNTMDVHLDLTAPTFV
WYINNSSEKVDWNKPL
SQYVANNETDYPSDLNIWKVDGESQWVYWLIENDPDSIFSLPHPIHLHGHDFVVLGRSPDANPIT
QTSYSFDPSLVKGDN
PVRRDVTMLPAAGWLLLAFKTDNPGAWLMHCHIAWHVAGGLSVTFLERLSDYRAGMNATDID
VLNSQCTAWDNYFPADDP
YPQDDSGI
>KAI7774707.1:6-590 hypothetical protein LA080_007977 [Diaporthe eres]
SLFVTGLLSLQPARAAPSSLLQPIELGQDVGCNTPTDRACWSDGFDITTDYETKSPLTGVVRPYTLT
LTEETNWKGPDGV
VKDFVMLVNGDIIGPTIHAQWGDTISVRVINNLETNAYTSQGTSIHWHGFRQFGTNLHDGVNGIT
ECPIPPKGGERQYTF
IARQYGSAWYHSHFSAQYGNGIIGAVQIDGPASTPYDIDLGPMLLTDYYHDTADNLVHYTETNGA
PPSDNVLFNGHAVNP
STGEGQYEKVTLTPGKRHRLRLINTSVDNNFQVSIVGHDMTVIASDFVPVNAFTTDSLFIAVGQRY
DVTIEAKEDIGNYW
FNVTFGGGKLCGASNTPYPAAILHYDGAPDSLPTNEGTRPRDHNCLDLSNLTPVVERKVSTAFTP
SAENTMEVQLPTGPK
FVWKINGKAIRTEWENPVAQYIASNRTDYPETNSIWQVDVVGQWVYWLIENDPEGAFSLPHPIH
LHGHDFLVVGRSPESA
PSSQTHYAFDASMLNGNNPVRRDVTMLPAAGWLLLAFKTDNPGVWLMHCHIAWHISGGLGMT
FLERLSDYQAGLPQGDVD
VLNKQCEAWNEYANNAPWPQADSGL
>KUI63476.1:19-588 Laccase [Valsa mali]
LAVPSLANRLEPILARQQESCNTATDRSCWTDGFDINTDYEISTPLTGVVRNYTLTLTEVDNWTGP
DGYVKEKVMMINDD
IIGPTIYAQWGDTISVRVINNLKTNGTSVHWHGIRQLETNLQDGVNGITECPLPPNSERVYTYIAH
QYGTSWYHSHFSAQ
YGNGVSGPIHIDGPASLPYDIDLGPFVLSDYYHKTADELVIYTETNGAPPSDNVLFNGTTVDPTTG
VGDYAKITLTPGKK
HRLRLINTSVENHFQLSIVNHNMTIIGSDFVPVEAYDTDSLFIGVGQRFDVIIDADQDVDNYWFNV
TYGGGGLCGSSNNP
YPAAIVQYDGAADGNPSDRGTTPKDHYCVDTMDIVPVISRTVPTSFTASSDNTLDFHLDLTAPTFV
WYINNSSEKVDWNK
PLSQYIANNQTDYPSNLNIWKVDGESQWVYWLIENDPDAIFSLPHPIHLHGHDFVVLGRSPDAN
PVTQTSYSFDPSLVNG
DNPVRRDVTMLPAAGWLLLAFKTDNPGAWLMHCHIAWHVAGGLSVTFLERLGDYRAGMNAT
DIDVLNSQCTAWDNYFPAD
DPYPQDDSGI
>XP_030991813.1:11-589 uncharacterized protein E0L32_001299 [Thyridium curvatum]
LLGMMLFRSVHAAPPAPVDVELVKRAGRNCNTASNRRTWCDGFSLDTDYEVPGNTPTGTVVSY
TFVLTEVNNWVGPDGVV
KNKFMLVNGQYPGPTIVADWGDTIQVTVINNLETNGTSIHWHGVRQLNSNIQDGANGLTECPIP
PKGGSKVYRFRATQYG
TSWYHSHFSAQYDNGIVGTILINGPASLPYEVDLGVFPITDYYYASADELLAITSRNAPPPADNILF
NGTNKHPSTGQGR
YAEVTLTPGKRHRLRIINTSADASFTVSLVDHDMTVIASDFVPVQSVTVNKLFVAIGQRFDVTIDAS
KVVGNYWFNVTFE
ASGACGASVNPHPAAVFRYQGAPKTDPTNPGTAVVPEPCVDKLDLVPVVTRTVSTSDFQPTIDNT
LNVSLDLAPTFVWKI
NSSAINVDWNRPVLEYVMEGNTSYPQSENIISVNGKDRWAYWLIENEVGFNIAHPLHLHGHDFV
VLGRSAVGAIGPFNAA

ADTRTLNGNNPARRDVTMLPAQGWVVLAFRTDNPGAWLFHCHIAWHVSAGLAVDFLENVGD
LRQNIAPGDKADFDRNCNA
WRAYFPEFDPFPKTDSGLR
>XP_040617398.1:1-597 laccase precursor [Sporothrix brasiliensis 5110]
MRSFISALAIVPSLLGSVLLARAAPSGLLEDASDLVARASTCNTPSNRACWTNGFDINTDYDASTP
VTNFTRHYTLVITE
ETNWTGPDGEVKELVMLINGTLPGPVISADWGDTLSITVINNLKSNGTSMHWHGIRQLNTNLQD
GVNGVTECPIPPGHSR
VYTFLATQYGSSWYHSHFSAQYGNGVVGTIQINGPASLPYDVDLGVFPVSDYYYHTSDYLVDYTK
NNGPPNSNNVLFNGT
NVHPVTGNGKYANVTLTPGKRHRLRIINISVENHFVFSLANHTMTIIAADLVPVNAMTVNELFVG
VGQRYDVTIDASQTP
GNYWFNATFVTNAQCGDSDNLTPAAIFHYAGSAGGLPTNPGSVATRTTCADLTNLTPVVKRTVP
VSGFVADSSNELNVTL
DLAEPGQLFTWKVNGTQEIISWEKPVDQYLLNSQTNWPNSDNVVVVDKKDQWVFWVVENDPV
IGIAHPMHLHGHDFMVVG
RADFNNPGPFKASDVSSFNGNNPVRRDVTMLPPLGWVAIAYKTDNPGTWLFHCHIAWHVSGGL
AVTFAERPTDFKAGVAA
ADKSALDTQCTNWNAYFPSQDPFPQSDSGLRKRRVNS
>KAH8896476.1:36-602 laccase [Thozetella sp. PMI_491]
RAATCHTPSNRACWTPGFNLYTDYESETPFTGVTRKYSIELTEHNNWVGPDGVTKEKVMLIDGK
VMGPTIYADWGDTVEV
TVKNSLECNGTSIHWHGIRQLYTNLADGANGITECPIAPGSTKVYTFVARQYGTGWYHSHFSAQY
GNGVVGAIVIHGPAS
LPYDIDLGAFPITDYYYNTADNLVAYTVNNGPPLSDNVLFNGTNVHPVTGAGTYANVTLTPGKR
HRLRIINTSVENHMQV
SLVGHSMTVIATDYVPVNAYTTDTLSVAIGQRYDVTIDATNHPGNYWFNVTYSDSGFCGGSKNPH
PAAVFHYKGAPGGLP
TDVGVKPKDSECLDQLNFTPVVQRFVPVNSFNPVVDNTLPVTVDLGSLNGSTPYPTFVWKVNGS
AIRVDWGKPIDQAILA
GNTTFAGSQNVIPTGTRANQWGYWLITNDPDGLFAVPHPIHLHGHDMVVVGRSPDGSAKTPIPY
VYNATVDKGRLTGINP
LRRDVTMLPPRGWIVVAFRTDNPGSWLMHCHIAWHASGGLSVDFVERPEDFRKGVSPADRAAL
DRNCNAWRGYYPTSDQY
QQQDSGI
>XP_016584179.1:1-597 laccase precursor [Sporothrix schenckii 1099-18]
MRSFISALAVVPSLLGSVFLAHAAPSGRLDDASDLVARASTCNTPSNRACWTNGFNISTDYEVSTP
VTNFIRHYTLVITE
ETNWTGPDGEVKELVMLINGTLPGPVISADWGDTLSITVINNLKTNGTSMHWHGIRQLNTNLQD
GVNGVTECPIPPGHSR
VYTFLATQYGSSWYHSHFSAQYGNGVVGTIQINGPASLPYDIDLGVFPVSDYYYHTSDYLVDYTK
NNGPPNSNNVLFNGT
NVHPVTGNGKYANVTLTPGKRHLLRIINISVENHFVFSLANHTMTIVAADLVPVNSMTVNELFVG
VGQRYDVTIDASQTP
GNYWFNATFVTNAQCGDSDNLTPAAVFHYAGSAGGLPTNPGSVATRTTCADLTNLTPVVKRTVP
VSGFVADSSNELNVTL
DLAEPGQLFTWKVNGTQEIISWEKPVDQYLLNSQTDWPNSDNVVVVDKKDQWVFWVVENDPV
IGIAHPMHLHGHDFMVVG
RADFNNPAPFKASDVASFKGDNPVRRDVTMLPPLGWVAIAYKTDNPGTWLFHCHIAWHVSGGL
AVTFAERPTDFKAGVAA
ADKSALDTQCTNWNNYFPSQDPFPQSDSGLRKRGINS
>XP_040778185.1:1-591 uncharacterized protein M406DRAFT_69386 [Cryphonectria parasitica EP155]

MPSFFRALFSGLIASQLSWAAPSLLHPLEPRQQPNCNTASNRACWISGSYDITTDYEVKTPLTGVV
RQYDLTLTQAENWL
GPDGVVKEDVMLVNGNILGPVIHAQWGDTISVTVTNNLKYNGTTIHWHGIRQLNTNLQDGVN
GITECPIPPNGGSKTYTF
IAHQYGTSWYHSHFSAQYGNGIVGAIQIDGPASLPYDIDLGPLVLSDYYYKTADELVVYTQSNAPP
ASDNVLFNGTNINP
ANTTQGQYKTITLTPGKRHRLRIINTSVENNFQVSIVGHSMTVIESDFVPVDSFTTDSLFVGIGQRY
DVTIDASQATDNY
WMNVTFGGGGFCGKSNNPYPAAIIHYNGASNSHPTNKGVAPADHECLDLLNLVPVVPRSIPTSG
FVAASDNTLDVQLSTT
TRKWTINGSTLDVDWGHPITQYVINKSTAWPSTDNVWLVEEANQWAYWLIENDPTATGNALPH
PIHLHGHDFVVLGRSPN
VSPTAQTPYTFTSSDVSSLNGNNPIRRDVVMLPPKGWLLIAFQTTNPGAWLMHCHIAWHVSAGL
GNTFLEQPSAFVAGLN
TNDVNQLNSQCKSWNAYYPSKDIFKQDDSGV
>ROW00470.1:29-589 hypothetical protein VPNG_07965 [Cytospora leucostoma]
ETIVARQEESCNTASNRACWTDGFDIHTDYEIKTPLTGVVRDYTLTLTESDNWTGPDGVIKKKVM
LVNGPTIYAQWGDTI
SVRVINNLETNGTSMHWHGIRQLNTNLQDGVNGITECPIPPGGERVYTYIAHQYGTSWYHSHFS
AQYGNGISGPIQIDGP
ASLNYDIDLGPFVLSDYYHRAADELVFDDAYNGPPTSQNVLFNGKTVNPDTGVGEYAKIKLTRGK
RHRLRLINTSVQHNL
VVSIVKHDMTVIGTDFVPVNSFTTNSLFIGVGQRYDVTIDASQAVDNYWFNVSLGTSQGSGFCGTS
ENLFPAAIVQYDGA
ADDIPTKEGTAPSDHNCLDSLDLVPVVSRTVPTSFTPSSGNTLDLTLSIISKAFVWNINGSPMKVDW
NKPLAQYVNKNET
DYASSENILKVDGDDDQWVYWLIENDLDGIYGSIPHPIHLHGHDFVVIGRSPDSAPASQTKYEFD
ASLVRGSNRVRRDVT
MIPAKGWLLLAFKTDNPGAWLMHCHIAWHVAGGLSNTFLERLDDYRASLKQADVDTLNEQCT
AWNAYFPAKDPYPQTDSG
I
>KAK0610108.1:1-484 laccase [Bombardia bombarda]
MLVNGPTLFADWGDFIQITVINKLRTNGTSIHWHGMQQRGTNLQDGANGVTECPIPPKGGTKV
YKFRATQYGTSWYHSHF
SAQYGNGVVGAIQINGPASLPYDIDLGTFPITDYYYNNNVLFNGTNVHPTTGNGKYANVTLTPG
KRHRLRIINTSVENHF
QLSLAGHQFTIIAADLIPVQAQTVSSLFLSVGQRLDVTIDASKTPGNYWFNATFGGSGFCGTSDNK
GAAAIFHYAGAAGG
LPKDPGAAPADHNCLDLTNLTPVVTRSLPVNNFIARPNNTLPVTVDVVSDAPLITWKVNGSAIN
VNWNKPVIDYVLTDNT
SYPREENVVQISQADQWVYWLIENDPDGPFSIPHPIHLHGHDFLIVGRSPDATPLSQTRYVFDPAV
DLPRLKGTNPARRD
TAMLPARGWLLLAFKTDNPGAWLMHCHIAWHVSAGLSIQFLERPKDLKNSISLVDKLAFNANCL
AWNLYFPALDPFPKID
SGLR
>EPE07365.1:77-650 laccase [Ophiostoma piceae UAMH 11346]
SGRTPSCNSANNRACWTSGYGGYSINTDYEAQTPTTGVTRQYTLLVTEEHNWTGPDGVVKELVM
LINGSLPGPNLVADWG
DQLEITVINGLEINGTSMHWHGIRQLNTNLQDGVNGVTECPIPPGSSRVYKFLAQQYGTSWYHS
HFSAQYGNGVVGTIQI
NGPASLNYDIDLGAFPVTDYYYDTSDNLVEYTENNGPPPSDNVLFNGTNVHPTSGAGKYASVTLT
PGKRHRLRIINTSVE

NHFVFSLVGHSMTIIAADFVPVQPQTVDQLFVGVGQRYDVTIDASMAVGNYWFNATFVTGAQC
GESYNPYPAAIFRYKGA
STTALPTTPGTTAAMDTCTDLSNLQPVLQRHISTKSFQSSKDNTLPVTLDLTQTGQLFSWRVNGTK
VAINWEKPVDQYVM
QGSTAYPPDDNVVTVTKKDQWIFWVIENDPTIGIAHPIHLHGHDFLLLGRSDPNNPTAFTSADM
AKLSGNNPVRRDVVML
PSLGWIVIGYKTDNPGTWLMHCHIAWHVSGGLAVNFLERPADLKARISAADKTAFDNQCAAWN
EYYPSKDPYRQSDSGLR
RREPLESGDHSVSA
>KAI3395873.1:3-564 hypothetical protein diail_810 [Diaporthe ilicicola]
LPTSLFLTGLLSLQQARAAPSSLLQPIEVRQGAGCNTASNRACWTDGFDINTDYEVKTPLTGVVRS
YTLTLTEQTNWKGP
DGVVKDFVMLVNGDIIGPTINAQWGDTISVKVINNLKSNVTECPIPPNGGERQYSFLATQYGNSW
YHSHFSAQLGNGIVG
PIQIDGPASLPYDIDLGPILLTDYYHDSADNLVLFTETNGPPPSDNVLFNGHAVNPSTGDGKYHTV
TITAGKRHRLRLIN
TSAENNFQVSIVGHNMTIIQSDFVPVNAFTTDSLFIGIGQRYDVTIEANQDVGNYWLNVTFGGGN
LCGTSENPHPAAILH
YDGAPDGLPTEDGTQPRDHNCLDLLDLKPVVERQLPTAFTPSAANTLDVQLPAGPKFVWKINN
KSIRTDWQNPIAQYVAA
NRTDYPETNSIFPVEAVNQWVYWLIENDPQGAFSLPHPIHLHGHDFLVVGRSPDSAPASQTRFAF
DASRLNGNNPVRRDV
TMLPAAGWLLLAFKTDNPGAWLMHCHIAWHIAGGLGLTFLERLSEYQAGLKQSDIDVLNKQCK
AWNDYFPKNAPWPQLDS
GL
>PSS05173.1:1-589 laccase [Coniella lustricola]
MPSFIGALFSGLIVSQLSWAAPSLLHSLDARQQQNCNTASNRACWIQGQYDINTDYEIDTPLTGVI
REYELTLTQAENWV
GPDGVVKDSVMLVNGDIIGPLIYAQWGDTISVKVTNNLEFNGTSIHWHGIRQLNTNLQDGVNGA
TECPIPPNGGTRTYTY
IAHQYGTSWYHSHFSAQYGNGVIGPIQIDGPASLPYDIDLGPLILSDYYYQTADYLVVYTQSNAPP
ASDNVLFNGTNINP
ANPSEGEYKTITLTPGKRHRLRLINTSVENHFQVSIVGHDMTVIGTDFVPVDSFTTSSLFVGIGQRY
DVTIDASQATDNY
WMNVTFGGGGGCGKSNNPYPAAIIHYDGAATGNPTNLGTVPTDHECLDLINFTPVVSRSVPTSSF
TATGDNTLDIESVAR
KWTIDGSSLVVDWGHPLAQYAIENSTAWPSSDNVYQIDAENQWVYWLIQNDPAETGPALPHPIH
LHGHDFVVLGRSPDEA
PQATTYYNFTSSDVFSLNGNNPLRRDVTMLPAKGWLLLAFKTDNPGAWLMHCHIAWHASGGL
GLSFLENMAGFSEGVSQS
DVDVLNSQCDAWNAYYPSNDAFLQDDSGV
>OAA56354.1:3-594 laccase [Niveomyces insectorum RCEF 264]
FLSSALALVPGLFGLVQAAPGLLSAHEGAELLSRESPSCNTPTNRACWSGNYNINTDYETTTPPGT
TRRYTLTVTEKENW
VGPDGEVKSLIMLINNQLPGPVLTADWGDTLVVTVVNNLKTNGTSMHWHGVRQLNTNLQDGV
NGVTECPIPPGGSRVYTF
RATQYGTTWYHSHFSAQYGNGVWGTIVINGPASLPYDIDLGPFPISDYYYDTADNLVEYTKNNPP
PASNNVLFNGTNVSP
LTGKGKYANVTLTHGKRHRLRIINTSVENHFVLSLANHQFTIIAADLVPVNAMTVDTLFVAIGQR
YDITIDASQAVDNYW
FNVTFVSGVQCGSSVNQFPAAIFHYAGASGDLPKHPGASVSAATCTDLINLTPVVSRTVPTTGVTA
NAGNELPVTLVLNE

NGELFTWKVNNTQMIINWNKPVDQYVMASNTNYPPSDNVVTVDKKNEWVYWVIENDPVIGIP
HPIHLHGHDFVLLGRSDP
NNAAPPPFNAATDLKNLSGSNPVRRDVVMLPSLGWIVIAFRTDNPGTWLMHCHIAWHVSGGLA
VNFLERPGDLRAGISAS
DKSVFDTQCANWNTYYPSEDPDHQDDSGLRIR
