# Supplementary material for: Precision Thermostability Predictions: Leveraging Machine Learning for Examining Laccases and Their Associated Genes
Source: Int J Mol Sci. 2024 Dec 4;25(23):13035. doi: 10.3390/ijms252313035 (PMC11641568; doi:10.3390/ijms252313035)
Supplement: Supplementary file 1 [file ijms-25-13035-s001.zip › ijms-3300419-supplementary.pdf]

Precision Thermostability Predictions: Leveraging Machine Learning for Examining Laccases and Their Associated Genes

Ashutosh Tiwari [1], Dyah Ika Krisnawati [2], Widodo [3], Tsai-Mu Cheng [4,5,6,*] and Tsung-Rong Kuo [1,7,*]

[1] International Ph.D. Program in Biomedical Engineering, College of Biomedical Engineering, Taipei Medical University, Taipei 11031, Taiwan.; d845112008@tmu.edu.tw
[2] Department of Nursing, Faculty of Nursing and Midwifery, Universitas Nahdlatul Ulama Surabaya, Surabaya 60237, East Java, Indonesia; dyahkrisna77@gmail.com
[3] Sekolah Tinggi Teknologi Pomosda, Nganjuk 64483, East Java, Indonesia; Widodoido7@gmail.com
[4] Graduate Institute for Translational Medicine, College of Medical Science and Technology, Taipei Medical University, Taipei 11031, Taiwan
[5] Taipei Heart Institute, Taipei Medical University, Taipei 11031, Taiwan
[6] Cardiovascular Research Center, Taipei Medical University Hospital, Taipei Medical University, Taipei 11031, Taiwan
[7] Graduate Institute of Nanomedicine and Medical Engineering, College of Biomedical Engineering, Taipei Medical University, Taipei 11031, Taiwan
* Correspondence: tmcheng@tmu.edu.tw (T.-M.C.); trkuo@tmu.edu.tw (T.-R.K.)

Supplementary materials

1. File S1− Codes and Related data: https://github.com/Ashu-design/Laccase-Precision-Thermostability-Predictions

2. File S2−Phylogenetic Tree:

   laccase.fas   Thermothelomyces thermophilus ATCC

3. Genus included for the study

No.	Genus
1	*Thermus*
2	*Parachaetomium*
3	*Chaetomium*
4	*Chaetomium*
5	*Chaetomium*
6	*Parathielavia*
7	*Chaetomium*
8	*Thermothielavioides*
9	*Staphylotrichum*
10	*Achaetomium*
11	*Melanocarpus*

12	*Dichotomopilus*
13	*Corynascus*
14	*Chaetomidium*
15	*Staphylotrichum*
16	*Chaetomium*
17	*Parathielavia*
18	*Canariomyces*
19	*Podospora*
20	*Podospora*
21	*Podospora*
22	*Podospora*
23	*Podospora*
24	*Apiosordaria*
25	*Lasiosphaeris*
26	*Cercophora*
27	*Podospora*
28	*Cladorrhinum*
29	*Cladorrhinum*
30	*Cladorrhinum*
31	*Podospora*
32	*Triangularia*
33	*Podospora*
